# Benzene- and pyridine-incorporated octaphyrins with different coordination modes toward two Pd^II^ centers

**DOI:** 10.1038/s41467-020-20072-9

**Published:** 2020-12-04

**Authors:** Le Liu, Zhiwen Hu, Fenni Zhang, Yang Liu, Ling Xu, Mingbo Zhou, Takayuki Tanaka, Atsuhiro Osuka, Jianxin Song

**Affiliations:** 1 College of Chemistry and Chemical Engineering, Key Laboratory of Chemical Biology and Traditional Chinese Medicine Research (Ministry of Education of China), Key Laboratory of the Assembly and Application of Organic Functional Molecules of Hunan Province, Hunan Normal University, 410081 Changsha, China; 2 Department of Chemistry, Graduate School of Science, Kyoto University, Sakyo-ku, Kyoto 606-8502 Japan

**Keywords:** Ligands, Chemical bonding, Synthetic chemistry methodology

## Abstract

Expanded porphyrins have received considerable attention due to their unique optical, electrochemical and coordination properties. Here, we report benzene- and pyridine-incorporated octaphyrins(1.1.0.0.1.1.0.0), which are synthesized through Suzuki-Miyaura coupling of α,α′-diboryltripyrrane with *m-*dibromobenzene and 2,6-dibromopyridine, respectively, and subsequent oxidation with 2,3-dicyano-5,6-dichlorobenzoquinone. Both octaphyrins are nonaromatic and take on dumbbell structures. Upon treatment with Pd(OOCCH_3_)_2_, the benzene-incorporated one gives a C_i_ symmetric NNNC coordinated bis-Pd^II^ complex but the pyridine incorporated one gives C_i_ and C_s_ symmetric NNNC coordinated bis-Pd^II^ complexes along with an NNNN coordinated bis-Pd^II^ complex bearing a transannular C–C bond between the pyrrole α-positions. In addition, these two pyridine-containing NNNC Pd^II^ complexes undergo trifluoroacetic acid-induced clean interconversion.

## Abstract (web summary)

The reactivity of expanded porphyrins is highly influenced by their scaffold architecture. Here, the authors construct two nonaromatic dumbbell-shaped benzene- and pyridine-incorporating octaphyrins via Suzuki-Miyaura cross-coupling; depending on the framework, different coordination modes are obtained upon metallation with Pd(II).

## Introduction

**Fig. 1:**
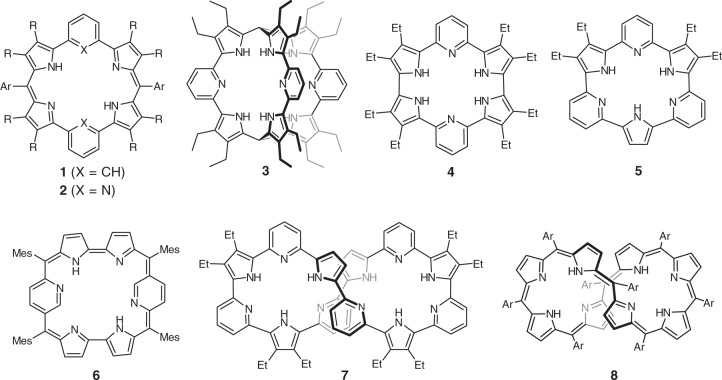
Representative structures of benzene-incorporated or pyridine-incorporated expanded porphyrinoids **1**–**8**. **1**, benzene-incorporated amethyrin analog; **2**, pyridine-incorporated amethyrin analog; **3**, cryptand-like molecule; **4**, cyclo[2]pyridine[4]pyrrole; **5**, cyclo[3]pyridine[3]pyrrole; **6**, pyridine-modified rubyrin; **7**, cyclo[6]pyridine[6]pyrrole; **8**, octaphyrin.

In recent years, considerable attention has been focused on expanded porphyrins in light of their attractive optical, electrochemical, and coordination properties^1–12^. Among these, pyridine-incorporated expanded porphyrins possess a unique position, since they showed interesting chemical behaviors arising from the basic pyridine subunits. More than two decades ago Corriu et al. reported the synthesis of benzene-incorporated and pyridine-incorporated amethyrin analogs **1** and **2** via a rational route^13^. Later, several analogous molecules possessing unique functions have been reported. As representative examples, cryptand-like molecule **3** exhibited unique properties such as binding three ethanol molecules and positive cooperativity in binding carboxylic acids^14^. Cyclo[2]pyridine[4]pyrrole **4** and cyclo[3]pyridine[3]pyrrole **5** showed protonation-induced realization of global conjugated networks^15^ and supramolecular assembling with dicarboxylic acids^16^. Pyridine-modified rubyrin **6** underwent protonation-induced flipping of the pyridine subunits^17^, and cyclo[6]pyridine[6]pyrrole **7** was synthesized as the largest pyridine-incorporating expanded porphyrin that showed characteristic conformational flexibility (Fig. 1)^14–23^.

Recently we explored various porphyrinoids by using Suzuki–Miyaura coupling. Reported examples include cyclic porphyrin rings, BODIPY-porphyrin hybrids, and earring porphyrins^24–28^. Despite of these studies, we thought that this coupling strategy could be applied to the synthesis of pyridine-incorporated expanded porphyrins. [36]Octaphyrins(1.1.1.1.1.1.1.1) **8** have been known to bind two metal ions within two semi-porphyrin-like pockets and their bis-Cu^II^ and bis-Co^II^ complexes were demonstrated to undergo quantitative splitting reactions to give the corresponding two metaloporphyrins almost quantitatively^29,30^.

Here, we report the synthesis of phenylene-incorporated and pyridine-incorporated octaphyrins that have remained largely unexplored. The presence of pyridine units into the octaphyrin scaffold leads to unexpected metalation modes and reactivity of the resultant metal complexes. Since the pyridine is quite basic it enables preferential protonation and metal coordination. The pyridine-incorporated octaphyrin coordinates Pd^II^ to form C_i_ and C_s_ symmetric NNNC bis-Pd^II^ complexes, and an NNNN coordinated bis-Pd^II^ complex bearing a transannular C–C bond between the pyrrole α-positions. The former two complexes can interconvert upon addition of trifluoroacetic acid (TFA).

## Results

### Synthesis and structural characterization of **12** and **14**

**Fig. 2:**
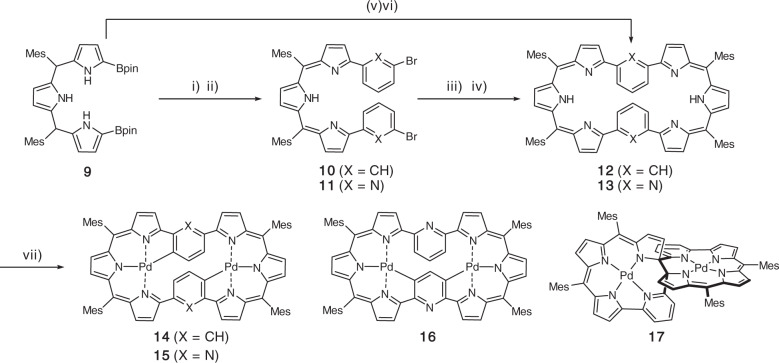
Syntheses of **12-17**. Reaction conditions: i) excess *m-*dibromobenzene or 2,6-dibromopyridine, Pd_2_(dba)_3_, PPh_3_, Cs_2_CO_3_, CsF, *p-*xylene or toluene/DMF, reflux, 48 h. ii) DDQ, CH_2_Cl_2_, r.t. iii) α,α′-diboryltripyrrane Pd_2_(dba)_3_, PPh_3_, Cs_2_CO_3_, CsF, *p-*xylene or toluene/DMF, reflux, 48 h. iv) DDQ, CH_2_Cl_2_, r.t. v) *m-*dibromobenzene or 2,6-dibromopyridine, Pd_2_(dba)_3_, PPh_3_ or XPhos, Cs_2_CO_3_, CsF, toluene/DMF, reflux, 48 h. vi) DDQ, CH_2_Cl_2_, r.t. vii) Pd(OAc)_2_, NaOAc, CHCl_3_/CH_3_OH, reflux. dba = dibenzylideneacetone, Mes = 2,4,6-trimethylphenyl, Bpin = pinacolatoboryl.

**Fig. 3:**
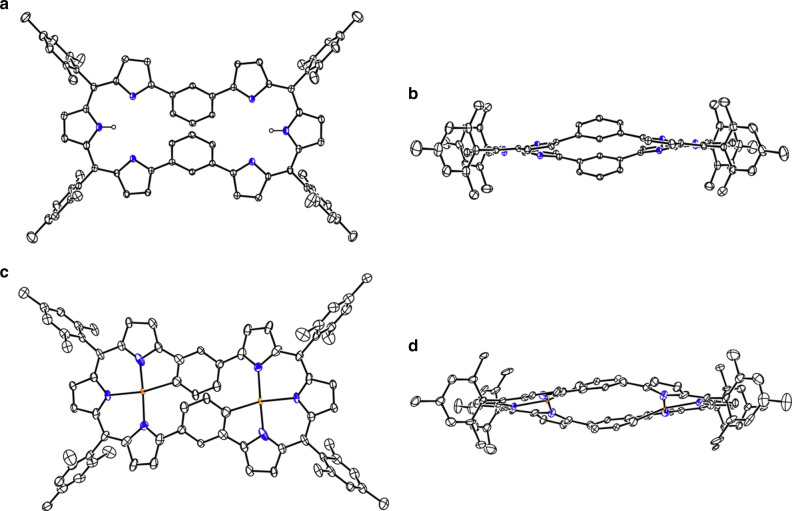
X-ray structures of **12** and **14**. **a** Top view and **b** side view of **12**. **c** top view, and **d** side view of **14**. The thermal ellipsoids are on 30% probability level. Hydrogen atoms except those connected to N atoms are omitted for clarity. Carbon atom, black ellipsoid; nitrogen atom, blue; palladium atom, orange; hydrogen atom small black ball.

1,3-Phenylene-incorporated and 2,6-pyridylene-incorporated octaphyrins(1.1.0.0.1.1.0.0) **12** and **13** are the target molecules in the present study. The key precursor α,α′-Diboryltripyrrane **9** was prepared by regioselective Ir-catalyzed borylation of 5,10-dimesityltripyrrane (Fig. 2)^28^. Firstly, we tried the synthesis of 1,3-phenylene-incorporating octaphyrin **12** by Suzuki-Miyaura coupling of **9** with *m-*dibromobenzene and subsequent oxidation with 2,3-dichloro-5,6-dicyano-1,4-benzoquinone (DDQ) but this reaction sequence gave linear precursor **10** in 53% yield. We then examined the cyclization of **10** with **9** under similar coupling and oxidation conditions. The equimolecular reaction of **9** and **10** yielded **12** in 3.8% yield but the yield of **12** could be improved to 8.0% when 1.2 equivalent of **9** was applied. The parent ion peak of **12** was detected at *m*/*z* = 1062.5479 (calcd for [C_76_H_66_N_6_]^+^ = 1062.5343 [M]^+^). The ^1^H nuclear magnetic resonance (NMR) spectrum shows a symmetric feature displaying a broad singlet at 13.00 ppm due to the pyrrolic NH protons, two doublets at 7.39 and 7.03 ppm and a singlet at 6.33 ppm due to the pyrrolic β-protons, and only three singlet signals due to the methyl protons of the mesityl groups. Collectively, these chemical shifts indicate a nonaromatic character for **12** (see Supplementary Fig. 4 and Supplementary Table 8). Fortunately, we obtained single crystals of **12** by slow diffusion of isopropanol vapor into its chlorobenzene solution. As shown in Fig. 3a, b, **12** takes a planar dumbbell structure with C_i_ symmetry, in which the two 1,3-phenylene units are pointing inward, being close to overlapping, and the two tripyrrin units are also pointing inward, forming two hemiporphyrin-like pockets. There is no particular bond-length alteration in the 1,3-phenylene units.

Encouraged by the hemiporphyrin-like pockets, we examined Pd^II^ metalation of **12**. A solution containing **12**, Pd(OAc)_2_, and NaOAc in a mixture of chloroform and methanol was refluxed for 24 h, which yielded **14** in 74% yield as a single product. The parent ion peak was detected at *m*/*z* = 1270.3018 (calcd for [C_76_H_62_N_6_Pd_2_]^+^ = 1270.3134 [M]^+^). The structure of **14** was also determined by single crystal X-ray diffraction analysis (Fig. 3c,d). Complex **14** shows a C_i_ structure, in which the two Pd^II^ ions take square planar coordination bound with the three pyrrolic nitrogen atoms and one carbon atom in the 1,3-phenylene unit.

### Synthesis and structural characterization of **13**, **15**, **16,** and **17**

**Fig. 4:**
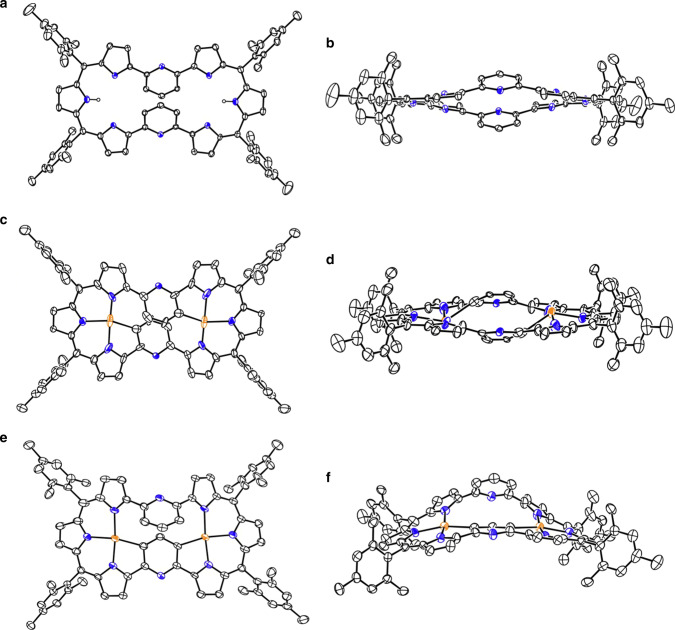
X-ray structures of **13**, **15,** and **16**. **a** Top view. **b** Side view of **13**. **c** Top view and d) side view of **15**. **e** Top view and **f** side view of **16**. The thermal ellipsoids are on 30% probability level. Hydrogen atoms are omitted for clarity. Carbon atom, black ellipsoid; nitrogen atom, blue; palladium atom, orange; hydrogen atom small black ball.

By following the stepwise synthetic protocol for **10**, α,α′-di(6-bromopyrid-2-yl)tripyrrin **11** was prepared by the reaction of **9** with 2,6-dibromopyridine in 48% yield, and octaphyrin **13** was synthesized by the reaction of **9** with **11** in 9.0% yield. The direct one-pot method gave **13** in 8.1% yield. The parent ion peak of **13** was observed at *m*/*z* = 1064.5239 (calcd for [C_74_H_64_N_8_]^+^ 1064.5248 [M]^+^) and the ^1^H NMR spectrum of **13** is similar to that of **12**, indicating its symmetric structure and a nonaromatic character. Octaphyrin **13** shows a planar dumbbell structure quite similar to that of **12**, in which the two hemiporphyrin-like cavities are secured (Fig. 4a, b).

Considering the high coordination ability of pyridine, it was thought that metalation behaviors of **13** might be different from those of **12**. Pd^II^ metalation of **13** under the same conditions afforded three Pd^II^ complexes **15**, **16**, and **17** in 20%, 60%, and 12% yield, respectively. The structures of **15** and **16** are analogous to that of **14** but that of **17** is substantially different. The parent ion peaks of **15** and **16** were observed at *m*/*z* = 1272.2944 (calcd for [C_74_H_60_N_8_Pd_2_]^+^ 1272.3038 [M]^+^) and *m*/*z* = 1272.3063 (calcd for [C_74_H_60_N_8_Pd_2_]^+^ 1272.3038 [M]^+^), respectively.

Structures of **15** and **16** were unambiguously determined by X-ray analysis as shown in Fig. 4c–f. Both complexes show the similar planar dumbbell structures but differ in Pd^II^ coordination modes. Namely, in **15** the Pd^II^ metals are respectively bound to different pyridine units, while the two Pd^II^ metals in **16** are bound to the same pyridine unit in a C_s_ symmetric manner. The Pd–N bond lengths are 1.969(5), 1.978(6), 2.053(6), 2.069(6), 2.071(5), and 2.085(6) Å, and Pd–C bond lengths are 2.077(7) and 2.102(7) Å in **16**. The sum of the bond angles around the Pd ions are 361.4(3)° and 361.1(3)° for **16**, being closer to the ideal square-planar coordination geometry. In line with these structures, the ^1^H NMR spectrum of **15** shows a set of doublets at 5.46 and 4.07 ppm due to the 2,6-pyridinylene units and that of **16** displays a singlet at 3.69 ppm and a set of mutually coupled doublet at 7.67 ppm and a triplet at 7.03 ppm. These chemical shifts can be explained by the local aromaticity of 1,3-phenylene and 2,6-pyridylene, which meet well with the NICS calculation. DFT calculation reveals that either in **15** or **16** *d* orbitals of Pd atoms are clearly involved in the HOMO and LUMO orbitals, which indicates a strong electron interaction between Pd atoms and octaphyrin scaffolds.

**Fig. 5:**
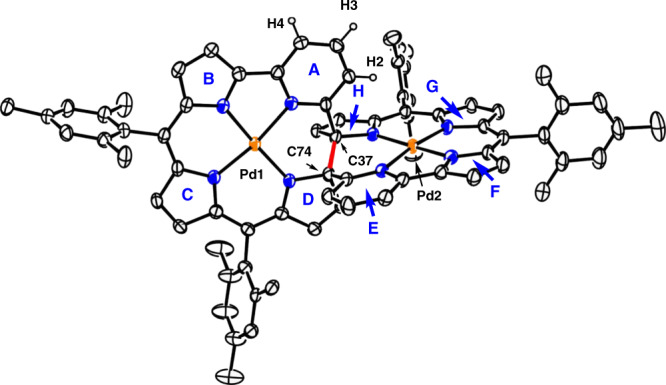
X-ray structure: **17**. The thermal ellipsoids are on 30% probability level. Hydrogen atoms except those in 2,6-pyridylene are omitted for clarity. Carbon atom, black ellipsoid; nitrogen atom, blue; palladium atom, orange; hydrogen atom small black ball.

**Fig. 6:**
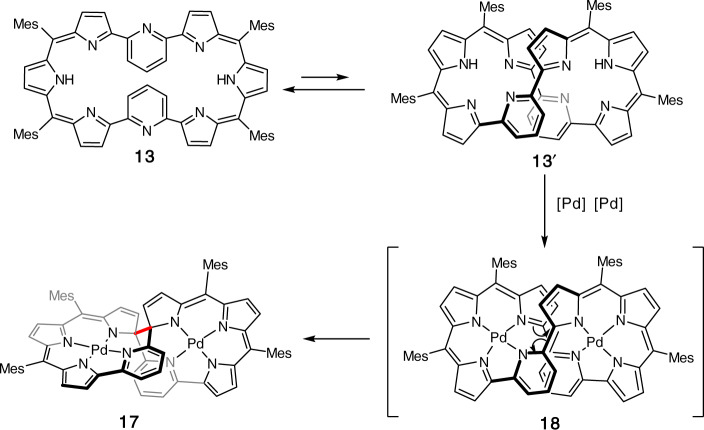
Plausible reaction mechanism from **13** to **17**. **13** to **13′**, conformation variation via σ bond rotation; **13′** to **18**, metal coordination; **18** to **17**, C–C bond (red) forming.

**Fig. 7:**
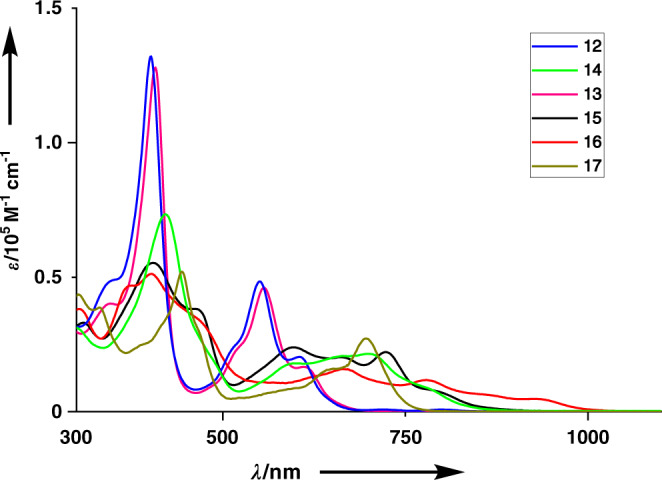
Absorption spectra of **12–17** in CH_2_Cl_2_. UV–Vis–NIR absorption spectra of **12** (blue), 14 (green), **13** (purple), **15** (black), **16** (red), and **17** (khaki). *λ*, wavelength; ε molar extinction coefficient.

The structure of **17** is shown in Fig. 5. The Pd1 atom is coordinated with three pyrroles B, C, D and pyridine A in a square planar manner with bond lengths of 1.922(3), 1.962(6), 1.942(4), and 2.024(5) Å. The Pd2 atom is bounded to the three nitrogen atoms of pyrrole rings F, G, H and pyridine E with distances of 1.926(4), 1.971(5), 1.947(4), and 2.023(4) Å. This hemi-porphyrin-like unit is relatively planar with a small mean plane deviation of 0.037(6) Å. Surprisingly, a new C–C bond is formed between the α-positions of the pyrroles D and H, causing a disruption of the macrocyclic conjugated network. This transannular bond length is rather large, being 1.568(8) Å. As a consequence, complex **17** exhibits a roughly perpendicular arrangement of two hemiporphyrin-like units. The parent ion peak was detected at *m*/*z* = 1274.3141 (calcd for [C_74_H_62_N_8_Pd_2_]^+^ 1274.3195 [M]^+^), which is two units larger than those of **15** and **16**. The ^1^H NMR and ^13^C NMR spectra are consistent with the structure. Characteristically, the ^13^C NMR spectrum shows a signal at 91.44 ppm due to the sp^3^-hybridized quaternary carbon atoms. The detailed reaction mechanism is not clear but may involve the bis-Pd^II^ complex **18**, which may be highly distorted and undergo a transannular C–C bond forming reaction to give **17** (Fig. 6). This process differs from the previous transannular reactions of expanded porphyrins (Fig. 7)^30–35^.

### Electrochemical and optical properties of 12, 13, 14, 15, 16, and 17

Cyclic voltammetry (CV) and differential-pulse voltammetry (DPV) experiments were conducted and the redox potentials are summarized in Table 1.

**Table 1:** Electrochemical properties of **12**, **14**, **13**, **15**, **16**, and **17** measured in PhCN.

Compound	*E*_ox.2_	*E*_ox.1_	*E*_red.1_	*E*_red.2_	*E*_red.3_	*E*_red.4_	Δ*E*_HL_
**12**	0.71^a^	0.63^a^	−1.31	−1.48	−1.80^a^		1.94
**14**	0.74^a^	0.48^a^	−1.07	−1.29	–1.84	−2.03	1.55
**13**	0.78^a^	0.70^a^	−1.18	−1.37	−1.69^a^		1.88
**15**	0.84^a^	0.62^a^	−0.92	−1.18	−1.74	−1.94	1.54
**16**	0.94^a^	0.47^a^	−0.97	−1.20	−1.81	−2.02	1.44
**17**	0.10^a^	−0.02^a^	−1.73				1.71

Potentials [V] vs. ferrocene/ferrocenium ion. Scan rate 0.05 Vs^–1^; working electrode, glassy carbon; counter electrode, Pt wire; supporting electrolyte, 0.1 M n-Bu_4_NPF_6_; reference electrode, Ag/AgNO_3_. Electrochemical HOMO-LUMO gaps (Δ*E*_HL_ = *E*_ox.1_ – *E*_red.1_ [eV]).

^a^Irreversible peaks.

In all cases, the oxidation waves are irreversible, indicating certain instabilities under oxidative conditions. On the other hands, the first and second reduction waves are reversible, showing a stepwise 1e^–^ reduction at –1.31 and –1.48 V for **12**, at –1.07 and –1.29 V for **14**. The Pd^II^ complexation result in a definitive anodic shift by about 0.2 V. It is also the case for **13**, the reduction peaks were observed at –0.92 and –1.18 V for **15** and at –0.97 and –1.20 V for **16**, both of which were anodically shifted from those of **13**. Interestingly, the first oxidation potential of **16** (0.47 V) is more negatively shifted than that of **15** (0.62 V), resulting in the smallest gap (Δ*E*_HL_) of 1.44 eV. The Δ*E*_HL_ values of **14** and **15** are almost identical, which is consistent with the orders of absorption red-shifts. The first oxidation potential of **17** was observed at –0.02 V, indicating its electron rich nature. This may be accounted for the presence of two 2-hydropyrrole units in **17**.

The UV-Vis absorption spectra of **12** and **13** are quite similar, exhibiting bands at 402, 551, and 598 nm and at 406, 555, and 610 nm, respectively. The absorption spectrum of **14** is broader and red-shifted, showing bands at 422, 599, and 700 nm. The absorption spectra of **15** and **16** are even broader and red-shifted. The spectral profiles of **15** and **16** are similar in the range of 350–500 nm but are different in the low energy region. Namely, **15** shows a tail up to 850 nm but **16** shows a tail reaching around 1000 nm. The absorption spectrum of **17** is also broader and red-shifted, exhibiting absorption bands at 444, 651, and 697 nm. Upon addition of acid, the absorption spectrum of **13** was largely changed probably due to the protonation of the pyridine moieties, since the addition of acid to CH_2_Cl_2_ solution of **12** did not lead to any changes in its UV–Vis spectrum (Supplementary Fig. 27).

### Mutual interconversion between 15 and 16

In the meanwhile, we found TFA-triggered clean mutual interconversion between **15** and **16**. In neutral or basic solutions, the mutual interconversion was not detected even at high temperature. Small amounts of TFA (0.05 or 0.2 equiv) accelerated the interconversion with molar ratios of **15**/**16** at the saturation point: 3.2 or 1.1 (see Supplementary Figs. 40 and 41). On the other hand, in the presence of 1.0 or 2.0 equiv TFA, the interconversion was rapid but gave **16** predominantly (see Supplementary Figs. 42 and 43). After certain reaction time, the interconversion showed the same saturation feature starting from either **15** or **16**, suggesting thermodynamic control. We thought that the relative stability of neutral **15** to **16** determined the equilibrium at low concentration of TFA and that of monoprotonated **15** to **16** determined the equilibrium at high concentration of TFA. TFA-d was then applied to explore the process of proton transferring process. Spectra of the product **16-d** indicated that C–H activation and the following C–Pd bonds deuteration underwent in the isomerization process. To better understand this process DFT calculations of the relative energies of **15** and **16** in neutral and monoprotonated forms were conducted (see Supplementary Figs. 56 and 57). In the monoprotonated forms, the most stable form was **16** protonated at the Pd-coordinated pyridine site, which is 9.0 kcal/mol more stable than **15**, in line with the experimental results. In contrast, the Gibbs free energy difference between neutral **15** and **16** is subtle (~2.7 kcal/mol), so that the equilibrium may be influenced by solvent effects or other experimental conditions.

## Discussion

In summary, the benzene and pyridine incorporating octaphyrins **12** and **13** were synthesized through Suzuki–Miyaura coupling reaction of the simple starting materials and subsequent oxidation with DDQ. These octaphyrins are nonaromatic and show dumbbell structures. The incorporation of pyridine units into the octaphyrin framework has strong influences such as the formation of the isomeric bis-Pd^II^ complexes **15** and **16** along with the rearranged complex **17**. Further, the complexes **15** and **16** underwent the TFA-induced mutual interconversion depending upon the concentration of TFA. Further exploration of core-modified expanded porphyrins is underway in our laboratories.

## Methods

### Materials, characterizations, and theoretical calculations

^1^H NMR (500 MHz) spectra were measured by a Bruker AVANCE-500 spectrometer, and chemical shifts were reported on the delta scale in ppm relative to CHCl_3_ as an internal reference (*δ* = 7.260 ppm). Assignments of ^1^H NMR were based on HH COSY spectra and D_2_O exchange experiments. UV/Vis absorption spectra were recorded on a Shimadzu UV-3600 spectrometer. X-ray crystallographic data were taken on an Agilent SuperNova X-ray diffractometer equipped with a large area CCD detector. Using Olex2, structures of compounds **12**–**17** were solved with the ShelXS structure solution program using Direct Methods and refined with the ShelXL refinement package using Least Squares minimization. Disordered solvent molecules were treated by SQUEEZE program of Platon. Redox potentials were measured by the cyclic voltammetry and differential pulse voltammetry method on an ALS660 electrochemical analyzed model (Solvent: PhCN, electrolyte: 0.1 M n-Bu_4_NPF_6_, working electrode: glassy carbon, reference electrode: Ag/AgNO_3_, Counter electrode: Pt wire, scan rate: 0.05 V/s, external reference: ferrocene/ferrocenium cation). Benzonitrile passed through alumina column was used for electrochemical analysis. Unless otherwise noted, materials obtained from commercial suppliers were used without further purification. All calculations were carried out using the Gaussian 09 program^36^. Initial geometries for **12–17** were obtained from X-ray structures. The structures were fully optimized without any symmetry restriction. Geometry optimizations in the ground state (*S*_0_) were performed by the density functional theory (DFT) method with restricted B3LYP (Becke’s three-parameter hybrid exchange functionals and the Lee–Yang–Parr correlation functional)^37,38^ level employing basis sets and pseudopotentials; 6–311G(d,p) for C, H, N^39^, and SDD for Pd^40^. NICS(0) values were calculated with GIAO method at the B3LYP level employing the same basis sets and pseudopotentials for geometry optimizations. Calculated chemical shifts were estimated relative to the magnetic shielding of a proton of chloroform (24.95 ppm) calculated at the same level.

### Synthesis of **10**

A toluene-DMF solution (3/1.5 mL) of **9** (178.4 mg, 0.25 mmol), 1,3-dibromobenzene (301.7 μL, 589.8 mg, 2.5 mmol), Pd_2_(dba)_3_ (22.9 mg, 0.025 mmol), PPh_3_ (26.2 mg, 0.1 mmol), Cs_2_CO_3_ (172.7 mg, 0.53 mmol), and CsF (77.5 mg, 0.51 mmol) was degassed through three freeze-pump*-*thaw cycles, and the reaction Schlenk tube was purged with argon. The resulting mixture was stirred at reflux for 48 h. The reaction mixture was diluted with CHCl_3_, washed with water, and dried over anhydrous sodium sulfate. After the solvent was removed, 2,3-dichloro-5,6-dicyano-1,4-benzoquinone (DDQ) (136.2 mg, 0.6 mmol) was added to the resulting mixture in CH_2_Cl_2_ and this reaction mixture was stirred for another 4 h. Evaporation of the solvent followed by silica-gel column chromatography (CH_2_Cl_2_/n-hexane as an eluent) and recrystallization with CH_3_OH to afford **10** as dark green solids (101.7 mg, 0.13 mmol, 53% yield). ^1^H NMR (500 MHz) (CDCl_3_) *δ* = 13.60 (br, 1H), 8.00 (s, 2H), 7.68 (d, 2H, *J* = 7.7 Hz), 7.20 (d, 2H, *J* = 8.0 Hz), 7.14 (d, 2H, *J* = 4.5 Hz), 6.97 (s, 4H), 6.88 (d, 2H, *J* = 4.5 Hz), 6.75 (t, 2H, *J* = 7.6 Hz), 6.19 (s, 2H), 2.38 (s, 6H), 2.18 (s, 12H) ppm; ^13^C NMR (126 MHz) (CDCl_3_): *δ* = 168.2, 152.7, 139.0, 137.7, 137.3, 137.0, 135.7, 133.6, 132.1, 131.0, 128.8, 127.9, 126.8, 124.1, 122.1, 121.0, 21.1, and 20.5 ppm; HRMS (*m*/*z*): [M]^+^ calcd. for C_44_H_37_Br_2_N_3_, 765.1349; found 765.1291.

### Synthesis of **11**

A toluene-DMF solution (3/1.5 mL) of **9** (178.4 mg, 0.25 mmol), 2,6-dibromopyridine (592.2 mg, 2.5 mmol), Pd_2_(dba)_3_ (22.9 mg, 0.025 mmol), PPh_3_ (26.2 mg, 0.1 mmol), Cs_2_CO_3_ (172.7 mg, 0.53 mmol), and CsF (77.5 mg, 0.51 mmol) was degassed through three freeze-pump-thaw cycles, and the reaction Schlenk tube was purged with argon. The resulting mixture was stirred at reflux for 48 h. The reaction mixture was diluted with CHCl_3_, washed with water, and dried over anhydrous sodium sulfate. After the solvent was removed, DDQ (136.2 mg, 0.6 mmol) was added to the resulting mixture in CH_2_Cl_2_ and this reaction mixture was stirred for another 4 h. The reaction mixture was passed through a short alumina column (CH_2_Cl_2_ as an eluent) and recrystallization with CH_3_OH to afford **11** as dark green solids (91.9 mg, 0.119 mmol, 47.8% yield). ^1^H NMR (500 MHz) (CDCl_3_): *δ* = 13.31 (br, 1H), 7.47 (m, 4H), 7.27 (d, 2H), 6.97 (s, 4H), 6.89 (d, 2H, *J* = 4.5 Hz), 6.66 (t, 2H, *J* = 7.6 Hz), 6.28 (s, 2H), 2.38 (s, 6H), 2.16 (s, 12H) ppm. ^13^C NMR (126 MHz) (CDCl_3_): *δ* = 168.4, 153.2, 153.0, 140.8, 139.4, 139.0, 138.0, 137.5, 137.1, 135.5, 133.4, 128.0, 127.5, 125.9, 121.9, 121.8, 21.1, 20.5 ppm; HRMS (*m*/*z*): [M+H]^+^ calcd. for C_42_H_36_Br_2_N_5_, 768.1332; found 768.1354.

### Synthesis of **12**

Route 1: A *p-*xylene-DMF solution (3/1.5 mL) of **9** (84 mg, 0.12 mmol), **10** (77.7 mg, 0.1 mmol), Pd_2_(dba)_3_ (18.5 mg, 0.02 mmol), PPh_3_ (21.2 mg, 0.08 mmol), Cs_2_CO_3_ (71.0 mg, 0.2 mmol), and CsF (33.4 mg, 0.2 mmol) was degassed through three freeze-pump-thaw cycles, and the reaction Schlenk tube was purged with argon. The resulting mixture was stirred at reflux for 48 h. The reaction mixture was diluted with CHCl_3_, washed with water, and dried over anhydrous sodium sulfate. After the solvent was removed, DDQ (65.8 mg, 0.29 mmol) was added to the resulting mixture in CH_2_Cl_2_, and this reaction mixture was stirred for another 8 h. The reaction mixture was passed through a short alumina column (CH_2_Cl_2_ as an eluent). Evaporation of the solvent followed by silica-gel column chromatography (CH_2_Cl_2_/n-hexane as an eluent) and recrystallization with CH_2_Cl_2_/MeOH afforded **12** as violet solids (8.4 mg, 0.008 mmol, 8.0% yield).

Route 2: A toluene-DMF solution (3/1.5 mL) of **9** (180 mg, 0.25 mmol), 1,3-dibromobenzene (25.6 μL, 49.5 mg, 0.21 mmol), Pd_2_(dba)_3_ (23 mg, 0.025 mmol), X-Phos (48 mg, 0.10 mmol), Cs_2_CO_3_ (136.0 mg, 0.42 mmol), and CsF (67.0 mg, 0.44 mmol) was degassed through three freeze-pump-thaw cycles, and the reaction flask was purged with argon. The resulting mixture was stirred for 48 h at reflux. The reaction mixture was diluted with CHCl_3_, washed with water, and dried over anhydrous sodium sulfate. After the solvent was removed, DDQ (136.2 mg, 0.6 mmol) was added to the resulting mixture in CH_2_Cl_2_ and this reaction mixture was stirred for another 8 h. The reaction mixture was passed through a short alumina column (CH_2_Cl_2_ as an eluent). Evaporation of the solvent followed by column chromatography on silica gel (CH_2_Cl_2_/n-hexane as an eluent) and recrystallization from n-hexane gave **12** as violet solids (7.9 mg, 0.007 mmol, 7.0% yield) ^1^H NMR (500 MHz) (CDCl_3_): *δ* = 13.0 (br, 2H, N–H), 8.34 (br, 2H, *m-*phenylene-H), 7.39 (d, 4H, *J* = 4.5 Hz, pyrrole-H), 7.25 (dd, 4H, *J* = 7.8, 1.5 Hz, *m-*phenylene-H), 7.03–7.00 (m, 12H, pyrrole-H and Ar-*m-*H), 6.33 (s, 4H, pyrrole-H), 5.63 (t, 2H, *J* = 7.7 Hz, *m-*phenylene-H), 2.40 (s, 12H, Me–H), 2.25 (s, 12H, Me–H), 2.18 (s, 12H, Me–H) ppm; ^13^C NMR (126 MHz) (CDCl_3_): *δ* = 169.2, 152.8, 138.9, 137.7, 137.7, 137.5, 135.8, 134.2, 132.9, 129.5, 128.1, 128.0, 127.3, 126.9, 124.7, 121.0, 21.3, 20.8, and 20.7 ppm; UV/Vis (CH_2_Cl_2_): *λ*_max_ (*ε*[M^–1^ cm^–1^]) = 402 (132,023), 551 (48,426), 598 (20,366) nm; HRMS (*m*/*z*): [M]^+^ calcd. for C_42_H_36_Br_2_N_5_, 1062.53; found 1062.49.

### Synthesis of **14**

**12** (20 mg, 0.019 mmol) was added to a round-bottomed 100 mL flask containing a magnetic bar and dissolved in CHCl_3_/MeOH (15/6 mL). Pd(OAc)_2_ (42.6 mg, 0.19 mmol) and NaOAc (18.7 mg, 0.22 mmol) was added, after being refluxed for 24 h, the solvent was evaporated in vacuo. The product was purified by column chromatography on silica-gel (CH_2_Cl_2_/n-hexane as an eluent). Recrystallization with n-hexane gave **14** (17.8 mg, 0.014 mmol, 73.6% yield) as green solids. ^1^H NMR (500 MHz) (CDCl_3_): *δ* = 8.67 (d, 2H, *J* = 1.8 Hz, C_6_H_3_-H), 7.59 (d, 2H, *J* = 4.8 Hz, pyrrole-H), 7.31 (d, 2H, *J* = 4.7 Hz, pyrrole-H), 7.25 (s, 2H, Ar-*m-*H), 7.03–7.01 (m, 6H, pyrrole-H and Ar-*m-*H), 6.98 (s, 2H, Ar-*m-*H), 6.94 (d, 2H, *J* = 4.3 Hz, pyrrole-H), 6.67 (d, 2H, *J* = 4.3 Hz, pyrrole-H), 6.59 (d, 2H, *J* = 4.3 Hz, pyrrole-H), 5.32 (dd, 2H, *J* = 7.6, 1.8 Hz, C_6_H_3_-H), 3.95 (d, 2H, *J* = 7.7 Hz, C_6_H_3_-H), 2.44 (s, 6H, Me–H), 2.41 (s, 6H, Me–H), 2.31 (s, 6H, Me–H), 2.17 (s, 6H, Me–H), 2.16 (s, 6H, Me–H), 2.07 (s, 6H, Me–H); ^13^C NMR (126 MHz) (CDCl_3_): *δ* = 172.4, 170.7, 170.6, 145.0, 144.2, 144.1, 140.5, 139.4, 139.2, 138.9, 137.8, 137.6, 137.6, 137.5, 137.4, 137.2, 136.7, 135.7, 133.9, 133.2, 132.6, 129.5, 128.0, 127.8, 127.7, 126.3, 126.2, 124.9, 123.7, 119.0, 21.2, 20.8, 20.7, 20.7, and 20.6 ppm; UV/Vis (CH_2_Cl_2_): *λ*_max_ (*ε*[M^–1^ cm^–1^]) = 422 (73,384), 599 (17,949), 700 (21,503) nm; HRMS (*m*/*z*): [M]^+^ calcd. for C_76_H_62_N_6_Pd_2_, 1270.3134; found 1270.3018.

### Synthesis of **13**

Route 1: A *p*-xylene-DMF solution (3/1.5 mL) of **9** (84.0 mg, 0.12 mmol), **11** (77.7 mg, 0.1 mmol), Pd_2_(dba)_3_ (18.5 mg, 0.02 mmol), PPh_3_ (21.2 mg, 0.08 mmol), Cs_2_CO_3_ (71.0 mg, 0.2 mmol), and CsF (33.4 mg, 0.2 mmol) was degassed through three freeze-pump-thaw cycles, and the reaction Schlenk tube was purged with argon. The resulting mixture was stirred at reflux for 48 h. The reaction mixture was diluted with CHCl_3_, washed with water, and dried over anhydrous sodium sulfate. After the solvent was removed, DDQ (65.8 mg, 0.29 mmol) was added to the resulting mixture in CH_2_Cl_2_ stirring for another 8 h. The reaction mixture was passed through a short alumina column (CH_2_Cl_2_ as an eluent). Evaporation of the solvent followed by silica-gel column chromatography (CH_2_Cl_2_/n-hexane as an eluent) and recrystallization with CH_2_Cl_2_/MeOH afforded **13** as violet solids (9.6 mg, 0.009 mmol, 9.0% yield).

Route 2: A toluene-DMF solution (3/1.5 mL) of **9** (180 mg, 0.25 mmol), 2,6-dibromopyridine (50.0 mg, 0.21 mmol), Pd_2_(dba)_3_ (38.8 mg, 0.042 mmol), PPh_3_ (44.2 mg, 0.17 mmol), Cs_2_CO_3_ (138.0 mg, 0.42 mmol), and CsF (69.5 mg, 0.45 mmol) was degassed through three freeze-pump-thaw cycles, and the reaction Schlenk tube was purged with argon. The resulting mixture was stirred at reflux for 48 h. The reaction mixture was diluted with CHCl_3_, washed with water, and dried over anhydrous sodium sulfate. After the solvent was removed, DDQ (136.2 mg, 0.6 mmol) was added to the resulting mixture in CH_2_Cl_2_ and this reaction mixture was stirred for another 8 h. The reaction mixture was passed through a short alumina column (CH_2_Cl_2_ as an eluent). Evaporation of the solvent followed by silica-gel column chromatography (CH_2_Cl_2_/n-hexane as an eluent) and recrystallization with CH_2_Cl_2_/MeOH afforded **13** as violet solids (9.1 mg, 0.009 mmol, 8.1% yield). ^1^H NMR (500 MHz) (CDCl_3_): *δ* = 13.43 (br, 2H, N–H), 7.63 (d, 4H, *J* = 4.5 Hz, pyrrole-H), 7.41 (d, 4H, J = 8.0 Hz, pyridine-H), 7.01-6.98 (m, 12H, pyrrole-H and Ar-*m-*H), 6.35 (s, 4H, pyrrole-H), 6.05 (t, 2H, *J* = 8.0 Hz, pyridine-H), 2.40 (s, 12H, Me–H), 2.26 (s, 12H, Me–H), 2.18 (s, 12H, Me–H) ppm; ^13^C NMR (126 MHz) (CDCl_3_): *δ* = 169.2, 153.1, 150.8, 139.3, 137.9, 137.6, 137.5, 137.4, 135.2, 134.2, 133.8, 128.2, 128.1, 125.8, 123.5, 121.3, 21.3, 20.8, and 20.7 ppm; UV/Vis (CH_2_Cl_2_): *λ*_max_ (ε[M^–1^ cm^–1^]) = 344 (40,195), 406 (128,444), 555 (46,048), 610 (16,662) nm; HRMS (*m*/*z*): [M]^+^ calcd. for C_74_H_64_N_8_, 1064.5248; found 1064.5239.

### Synthesis of **15**, **16**, and **17**

**13** (19.1 mg, 0.018 mmol) was added to a round-bottomed 100 mL flask containing a magnetic bar, and dissolved in CHCl_3_/MeOH (15/6 mL). Pd(OAc)_2_ (40.5 mg, 0.18 mmol) and NaOAc (17.8 mg, 0.21 mmol) was added, after being refluxed for 24 h, the solvent was evaporated in vacuo. The residue was purified by column chromatography on silica-gel (CH_2_Cl_2_/n-hexane as an eluent), three fractions were obtained. The first fraction was recrystallized with CH_2_Cl_2_/CH_3_OH to afford **17** (2.8 mg, 2.2 μmol, 12.0% yield) as green solids. The second fraction was recrystallized with CH_2_Cl_2_/n-hexane to afford **15** (4.58 mg, 3.6 μmol, 20.0% yield) as dark green solids. The third fraction was recrystallized with CH_2_Cl_2_/CH_3_OH to afford **16** (13.7 mg, 10.8 μmol, 60.0% yield) as navy blue solids.

**15:** ^1^H NMR (500 MHz) (CDCl_3_): *δ* = 8.00 (d, 2H, *J* = 4.7 Hz, pyrrole-H), 7.43 (d, 2H, *J* = 4.7 Hz, pyrrole-H), 7.34 (d, 2H, *J* = 4.7 Hz, pyrrole-H), 7.06–7.02 (m, 6H, Ar-*m-*H), 6.98 (s, 2H, Ar-*m-*H), 6.94 (d, 2H, *J* = 4.7 Hz, pyrrole-H), 6.74 (d, 2H, *J* = 4.5 Hz, pyrrole-H), 6.65 (d, 2H, *J* = 4.3 Hz, pyrrole-H), 5.46 (d, 2H, *J* = 7.6 Hz, pyridine-H), 4.07 (d, 2H, *J* = 7.6 Hz, pyridine-H), 2.44 (s, 6H, Me–H), 2.42 (s, 6H, Me–H), 2.31 (s, 6H, Me–H), 2.18 (s, 6H, Me–H), 2.13 (s, 6H, Me–H), 2.09 (s, 6H, Me–H) ppm; ^13^C NMR (126 MHz) (CDCl_3_): *δ* = 171.8, 170.7, 162.4, 157.4, 146.2, 144.9, 144.7, 144.1, 141.7, 140.6, 139.6, 138.4, 138.1, 137.7, 137.6, 137.6, 137.3, 136.8, 136.4, 135.5, 134.0, 133.4, 129.2, 128.2, 128.2, 127.0, 125.6, 121.9, 120.5, 21.5, 21.1, 21.0, and 21.0 ppm; UV/Vis (CH_2_Cl_2_): *λ*_max_ (ε[M^–1^ cm^–1^]) = 309 (33,094), 405 (55,309), 461 (38,258), 597 (23,944), 660 (20,273), 723 (22,148) nm; HRMS (*m*/*z*): [M]^+^ calcd. for C_74_H_60_N_8_Pd_2_, 1272.3038; found 1272.2944.

**16:** ^1^H NMR (500 MHz) (CDCl_3_): *δ* = 7.67 (d, 2H, *J* = 8.0 Hz, pyridine-H), 7.14 (d, 2H, *J* = 4.5 Hz, pyrrole-H), 7.03 (t, 1H, *J* = 7.7 Hz, pyridine-H), 6.95–6.92 (m, 12H, Ar-*m-*H and pyrrole-H), 6.52 (d, 2H, *J* = 4.9 Hz, pyrrole-H), 6.25 (d, 2H, *J* = 4.3 Hz, pyrrole-H), 6.22 (d, 2H, *J* = 4.5 Hz, pyrrole-H), 3.69 (s, 1H, pyridine-H), 2.37 (s, 6H, Me–H), 2.35 (s, 6H, Me–H), 2.21 (s, 6H, Me–H), 2.15 (s, 6H, Me–H), and 2.12 (s, 12H, Me–H) ppm; ^13^C NMR (126 MHz) (CDCl_3_): *δ* = 175.9, 169.9, 160.9, 155.2, 149.3, 147.4, 146.6, 144.7, 143.0, 140.5, 139.9, 138.0, 137.8, 137.3, 137.2, 137.2, 137.1, 136.8, 135.0, 134.4, 134.2, 133.4, 128.1, 128.0, 127.9, 127.4, 127.0 126.9, 123.2, 121.7, 21.3, 20.8, 20.6, 20.6, and 20.5 ppm; UV/Vis (CH_2_Cl_2_): *λ*_max_ (ε[M^–1^ cm^–1^]) = 403 (51,227), 556 (10,987), 664 (15,863), 777 (11,779), 925 (4759) nm; HRMS (*m*/*z*): [M]^+^ calcd. for C_74_H_60_N_8_Pd_2_, 1272.3038; found 1272.3063.

**17:** ^1^H NMR (500 MHz) (CDCl_3_): *δ* = 7.74 (d, 2H, *J* = 5.3 Hz, pyrrole-H), 7.44 (t, 2H, *J* = 7.9 Hz, pyridine-H), 7.31–7.29 (m, 4H, pyridine-H), 6.98–6.93 (m, 10H, Ar-*m-*H and pyrrole-H), 6.71 (d, 2H, *J* = 5.0 Hz, pyrrole-H), 6.26–6.24 (m, 4H, pyrrole-H), 6.01 (d, 2H, *J* = 5.3 Hz, pyrrole-H), 2.38 (s, 6H, Me–H), 2.31 (s, 6H, Me–H), 2.15 (s, 6H, Me–H), 2.09 (s, 6H, Me–H), 2.07 (s, 6H, Me–H), 2.03 (s, 6H, Me–H); ^13^C NMR (126 MHz) (CDCl_3_): *δ* = 161.2, 157.1, 156.2, 154.7, 147.0, 140.4, 139.9, 139.4, 138.9, 138.0, 137.8, 136.8, 136.7, 134.8, 134.6, 134.5, 133.5, 131.8, 128.1, 128.0, 127.8, 127.7, 125.2, 124.6, 117.0, 116.9, 113.2, 112.1, 100.3, 90.4, 21.2, 21.1, 21.1, 21.0, 20.7, and 20.6 ppm; UV/Vis (CH_2_Cl_2_): *λ*_max_ (ε[M^–1^ cm^–1^]) = 332 (38,615), 444 (51,873), 651 (15,961), 697 (27,095) nm; HR-MS (MALDI-TOF-MS): HRMS (*m*/*z*): [M]^+^ calcd. for C_74_H_62_N_8_Pd_2_, 1274.3195; found 1274.3141.

## Supplementary information

Supplementary Information

Peer Review File

## Data Availability

The X-ray crystallographic coordinates for structures reported in this study have been deposited at the Cambridge Crystallographic Data Centre (CCDC), under deposition numbers 1959823, 1959827, 1959825, 1959828, 1959830, and 1975890 (**12**, **13**, **14**, **15**, **16**, and **17**). These data can be obtained free of charge from The Cambridge Crystallographic Data Centre via www.ccdc.cam.ac.uk/data_request/cif. The authors declare that all other data supporting the findings of this study are available within the paper and its supplementary information files.
