# Supplementary material for: Benzene- and pyridine-incorporated octaphyrins with different coordination modes toward two PdII centers
Source: Nat Commun. 2020 Dec 4;11:6206. doi: 10.1038/s41467-020-20072-9 (PMC7718233; doi:10.1038/s41467-020-20072-9)
Supplement: Supplementary file 1 — Supplementary Information [file 41467_2020_20072_MOESM1_ESM.pdf]

Supplementary Information for

Benzene- and pyridine-incorporated octaphyrins with different coordination modes toward two Pd^{II} centers

Liu et al.

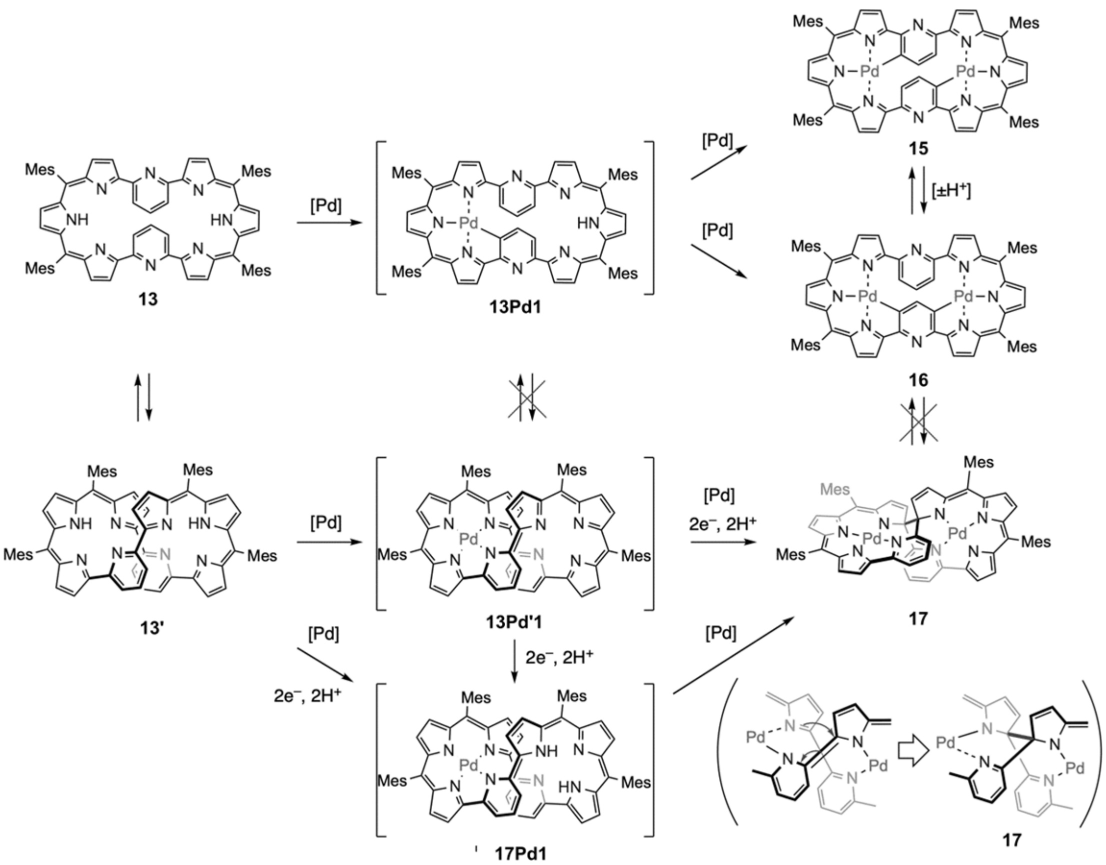

Supplementary Figure 1. Plausible reaction paths.

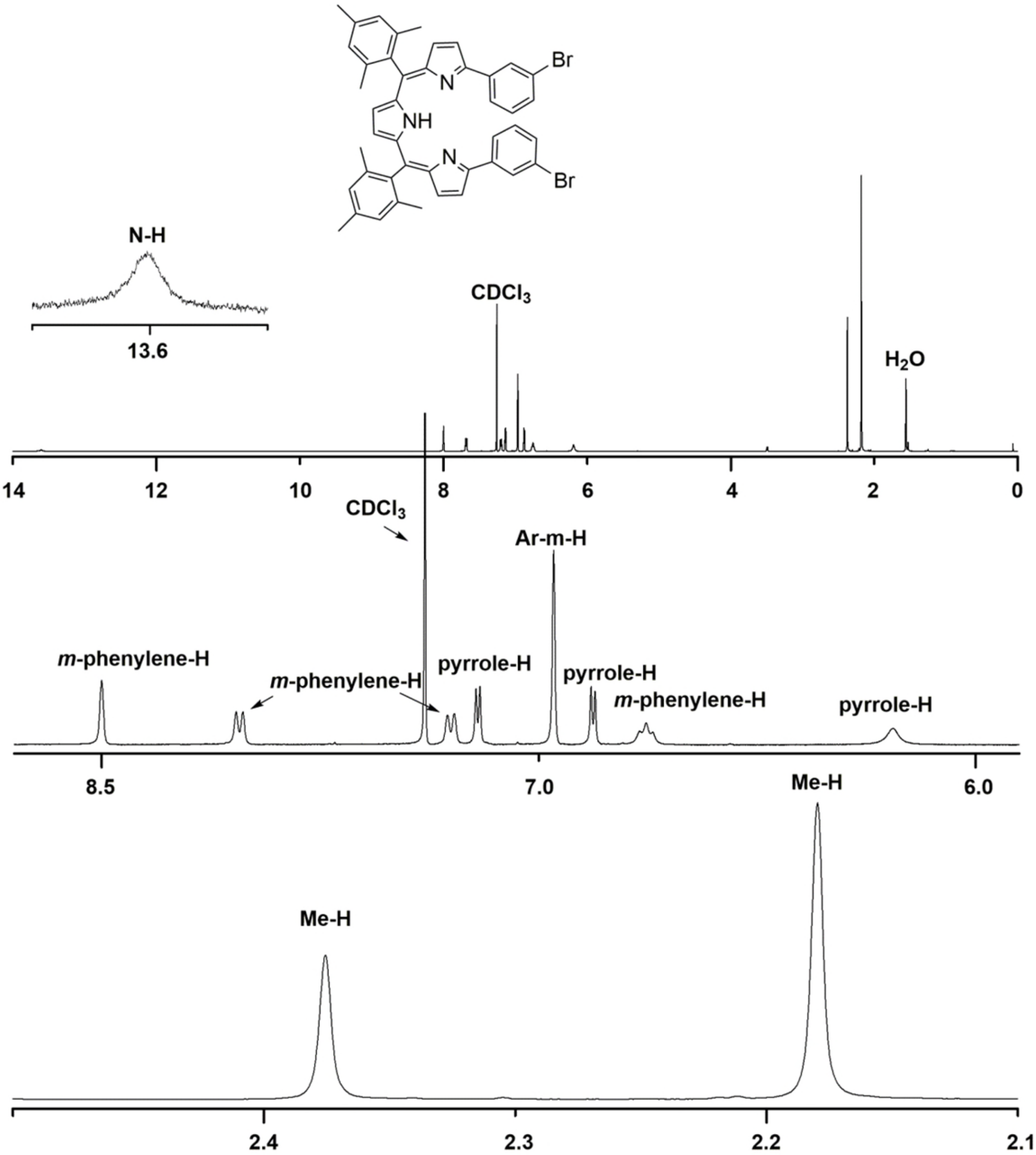

Supplementary Figure 2. ^{1}H NMR spectrum of **10** in $CDCl_3$

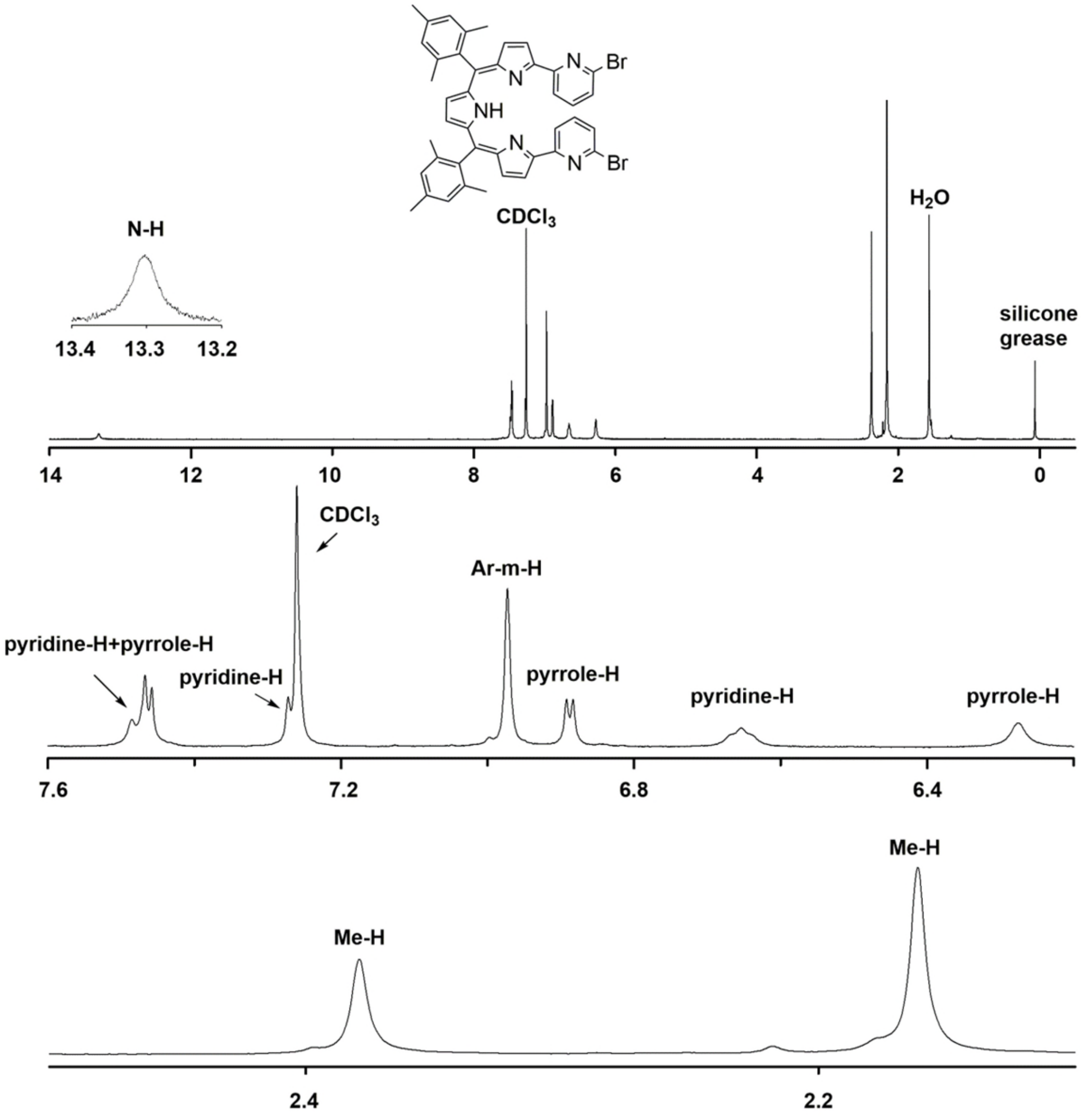

Supplementary Figure 3. ^{1}H NMR spectrum of **11** in $CDCl_3$

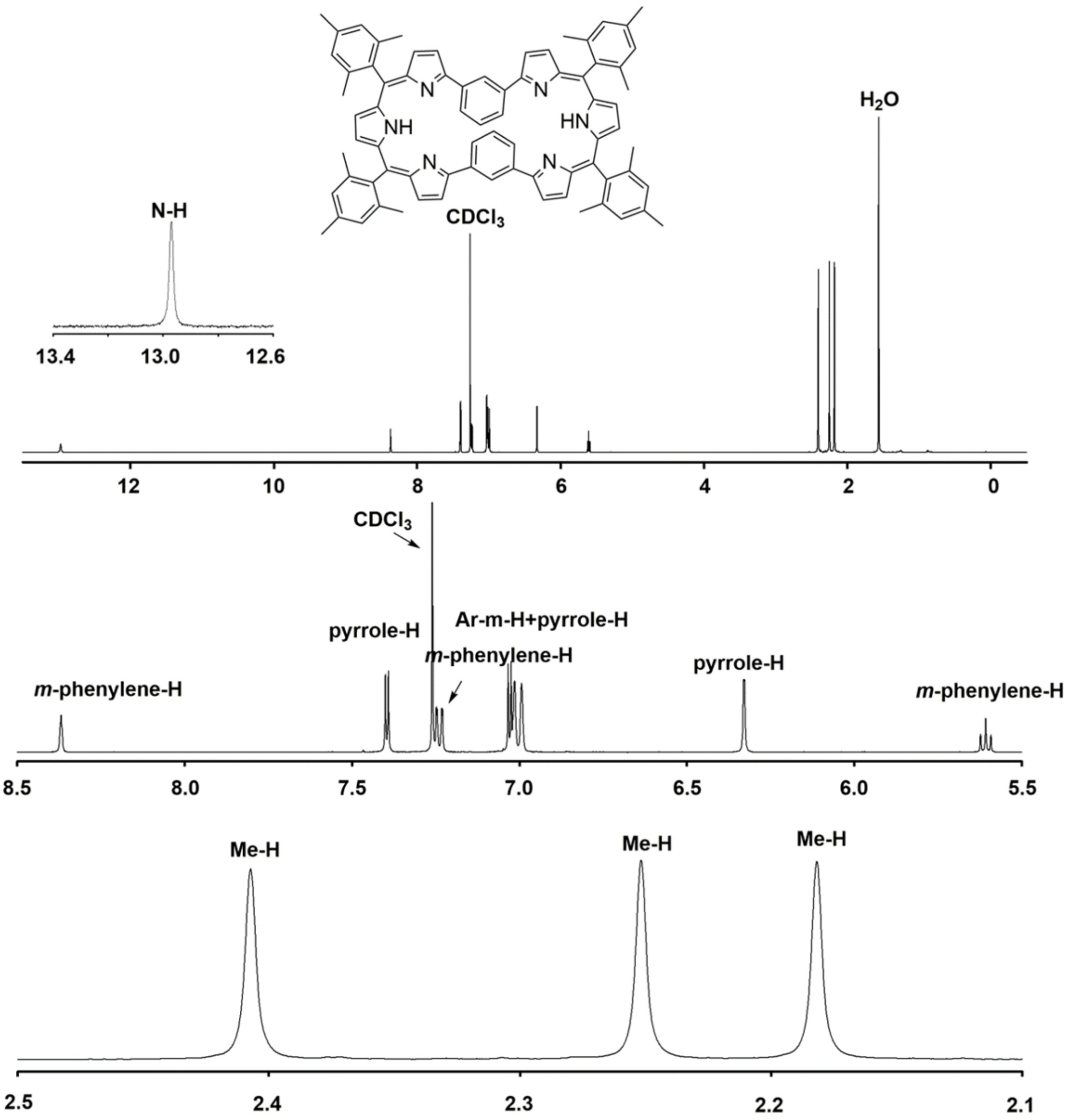

Supplementary Figure 4. ^{1}H NMR spectrum of **12** in $CDCl_3$

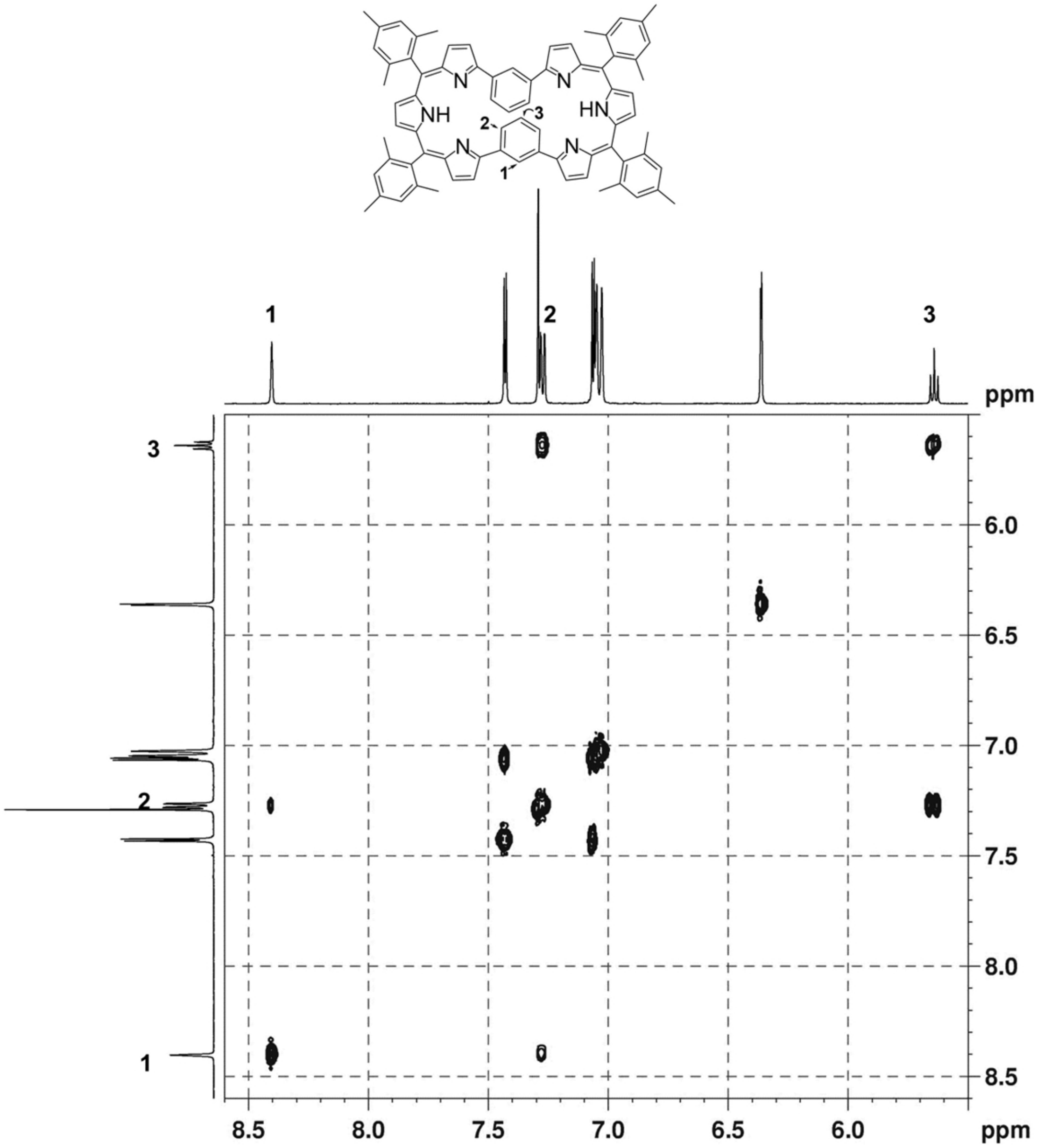

Supplementary Figure 5. COSY spectrum of **12** in $CDCl_3$

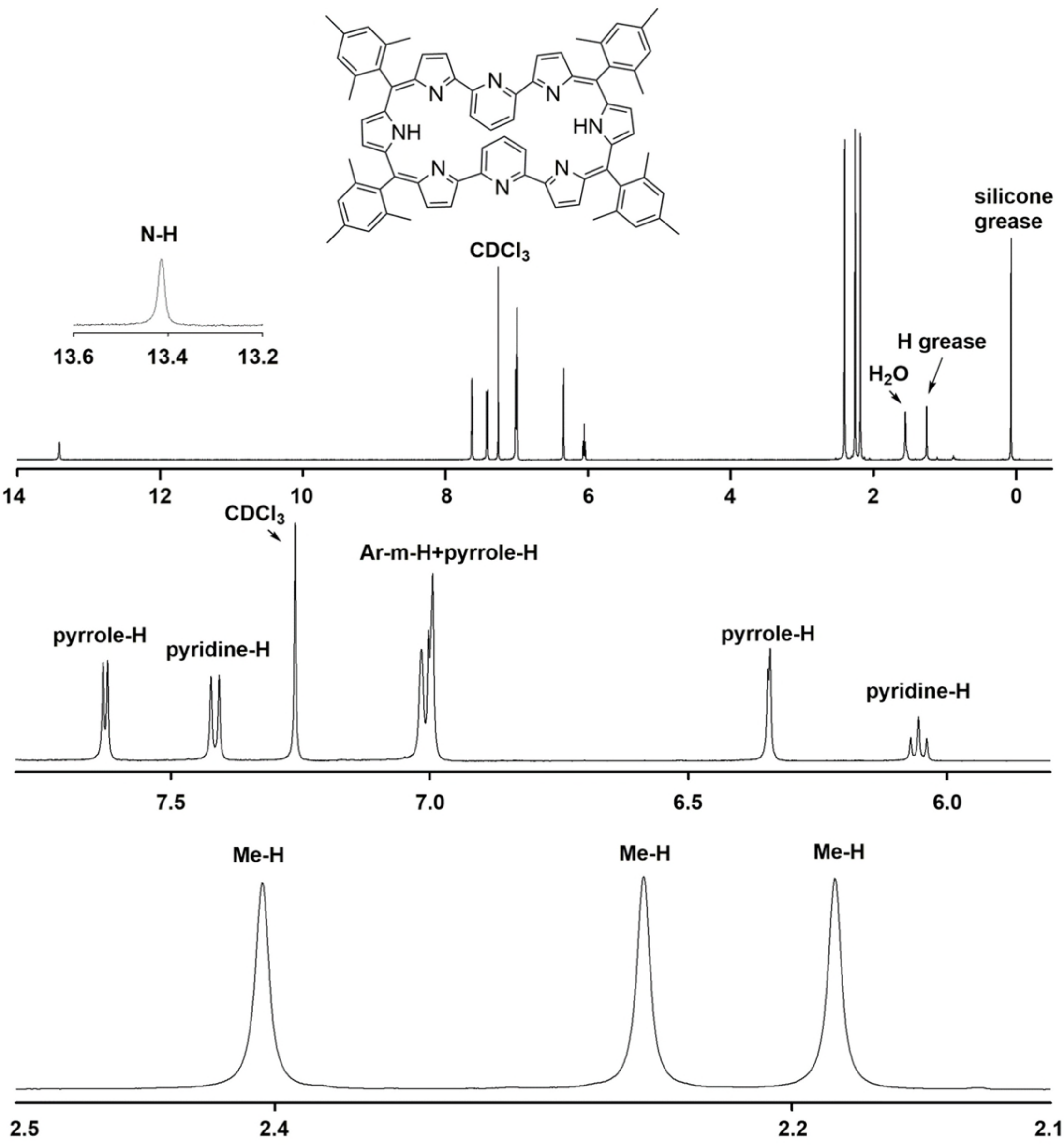

Supplementary Figure 6. ^{1}H NMR spectrum of **13** in $CDCl_3$

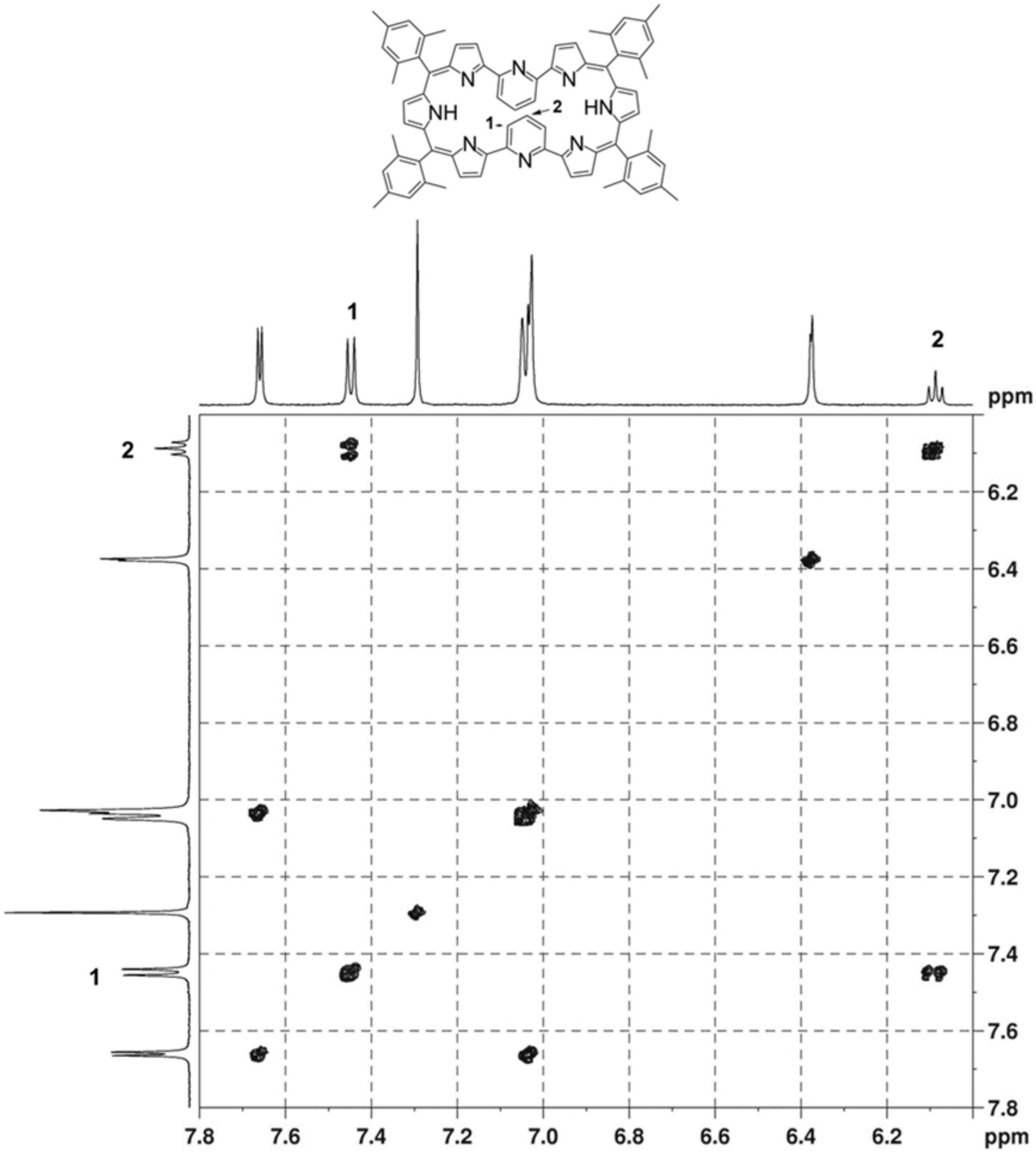

Supplementary Figure 7. COSY spectrum of **13** in $CDCl_3$

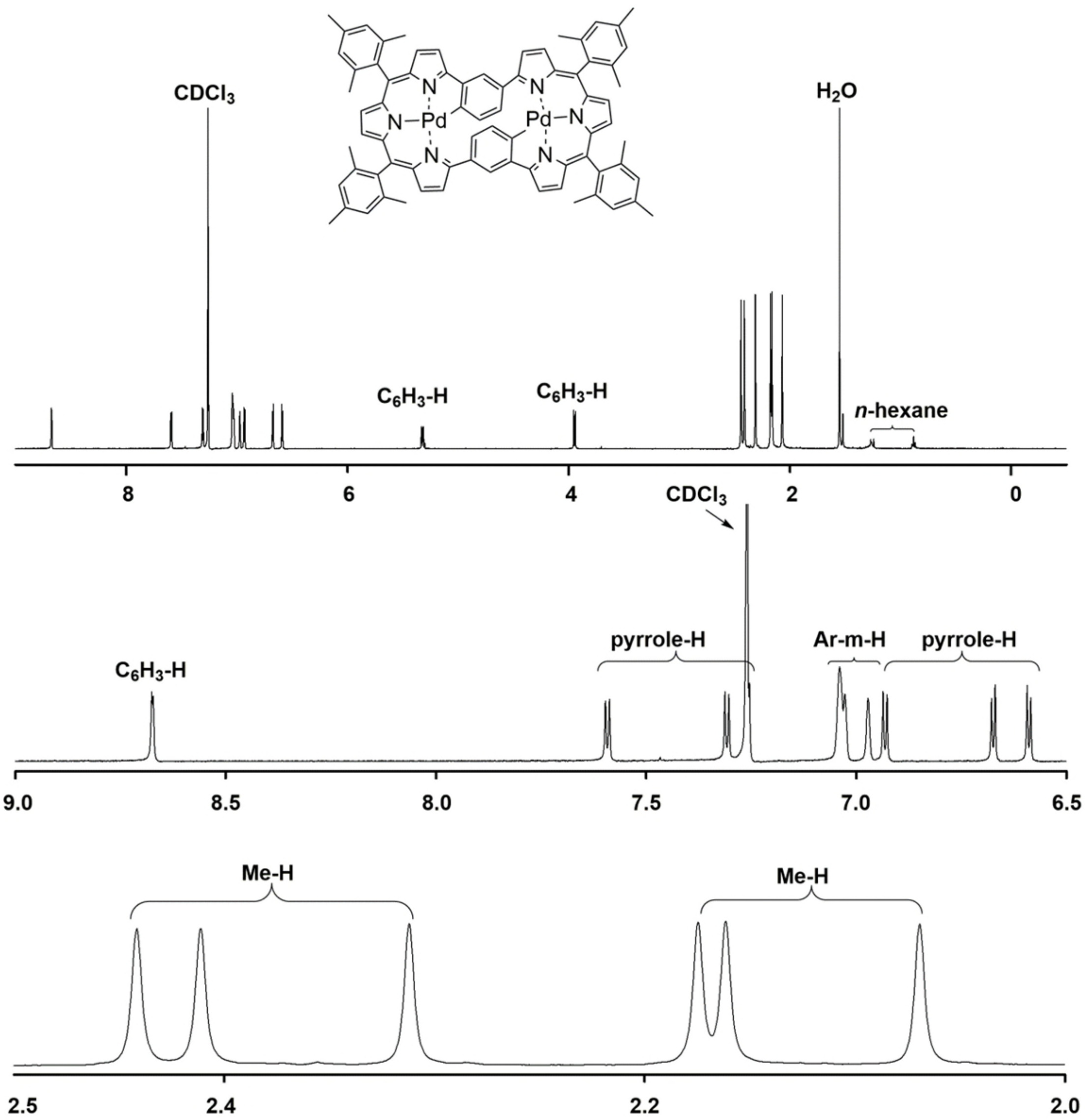

Supplementary Figure 8. ^{1}H NMR spectrum of **14** in $CDCl_3$

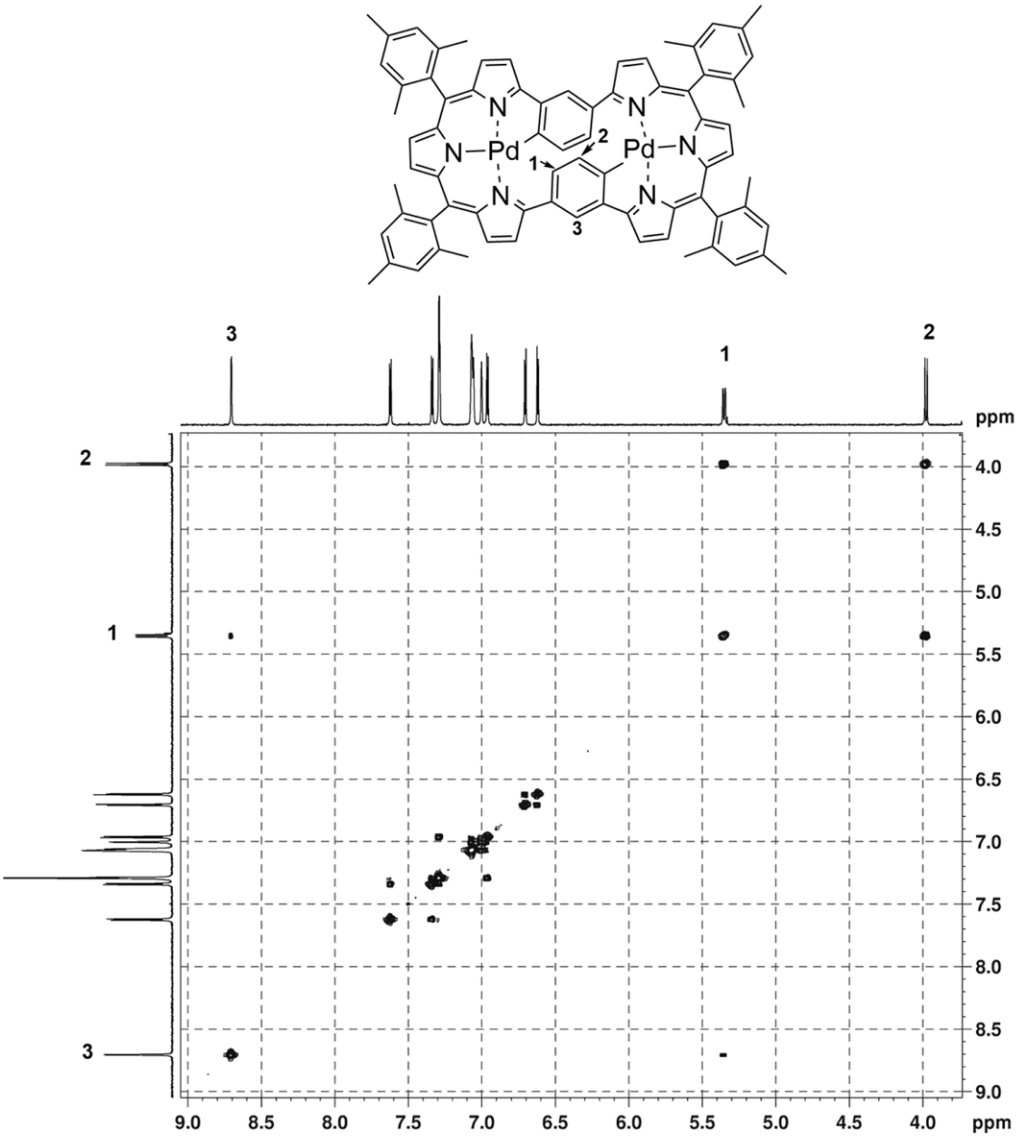

Supplementary Figure 9. COSY spectrum of **14** in $CDCl_3$

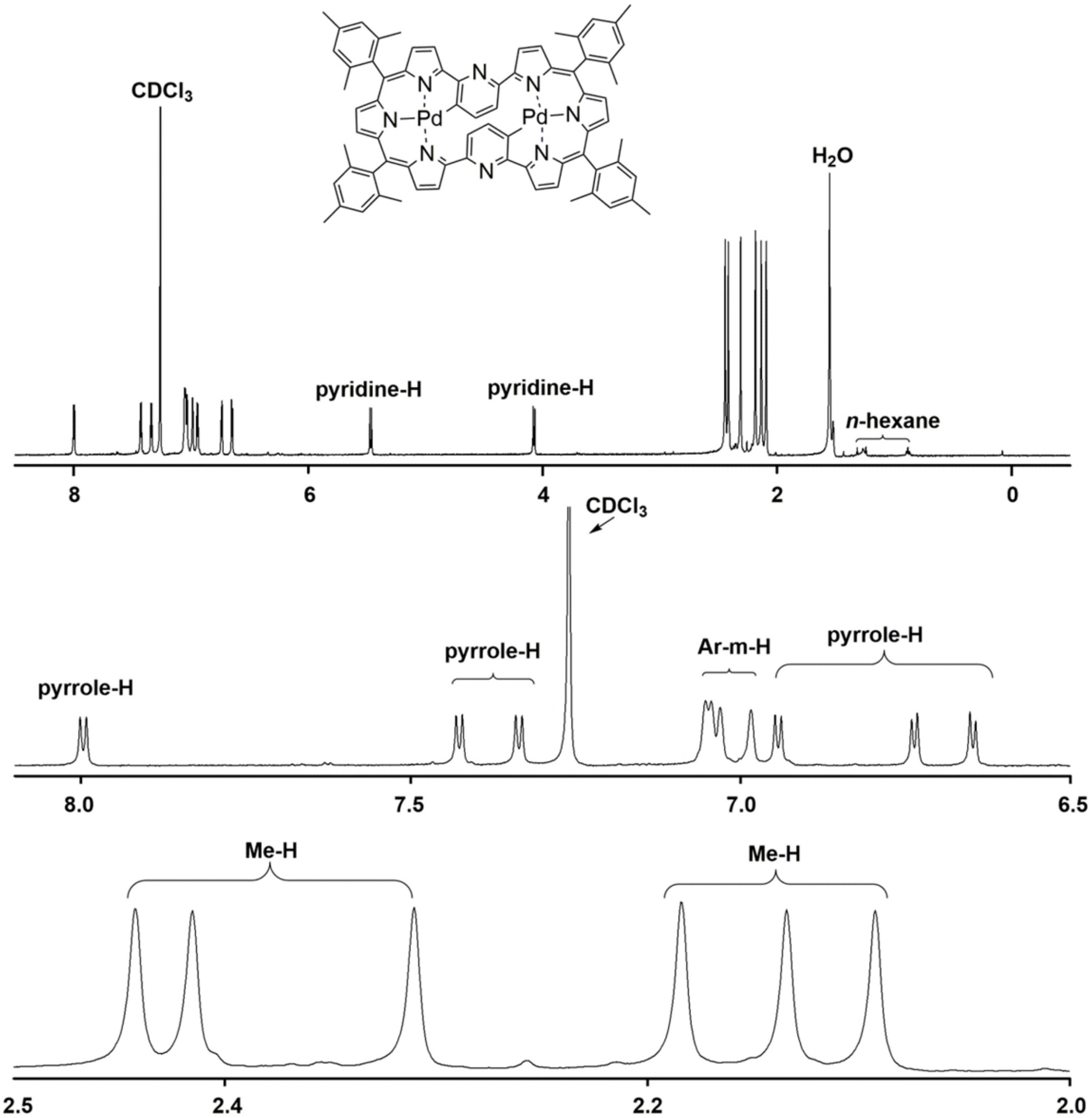

Supplementary Figure 10. ^{1}H NMR spectrum of **15** in $CDCl_3$

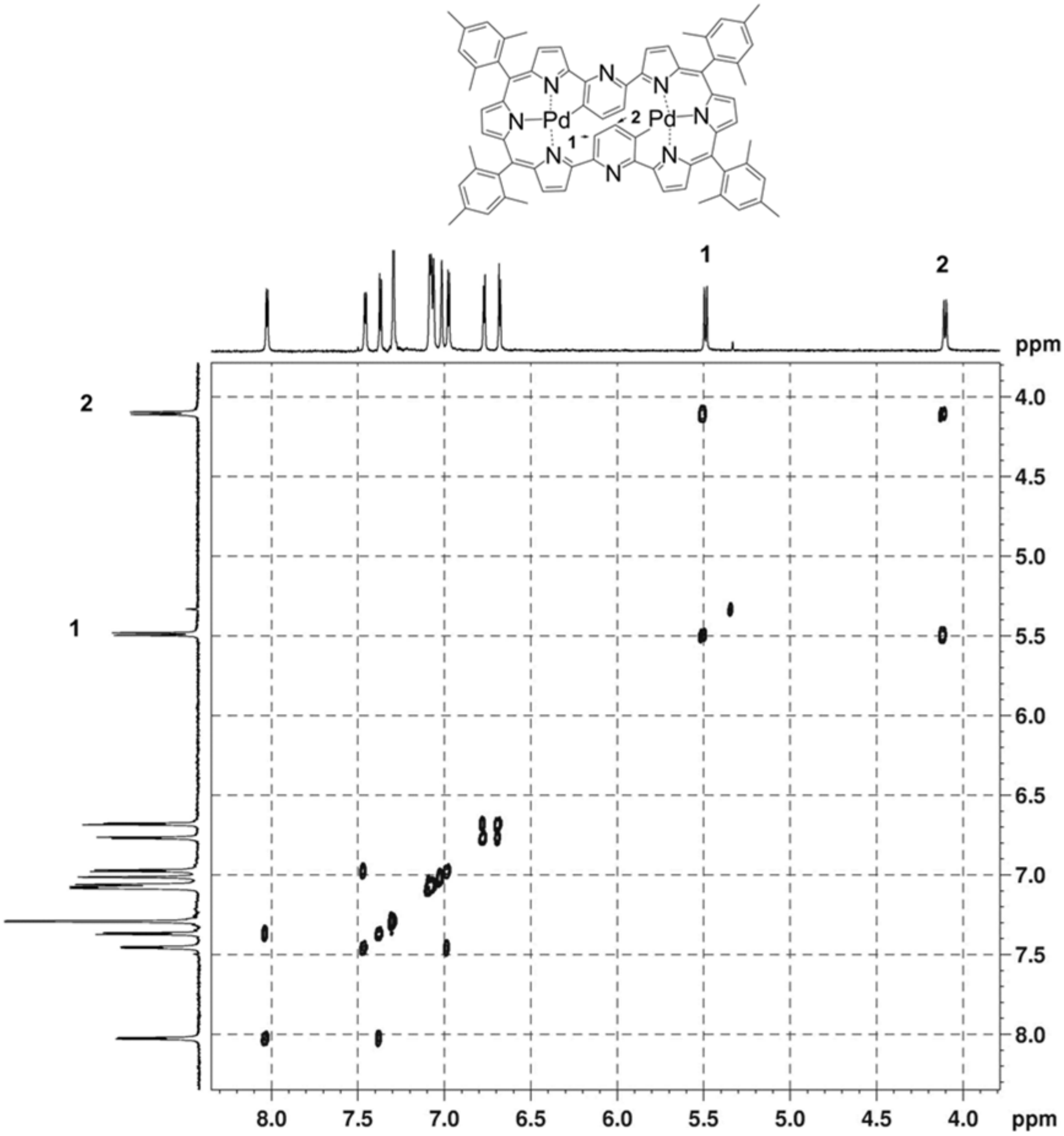

Supplementary Figure 11. COSY spectrum of **15** in $CDCl_3$

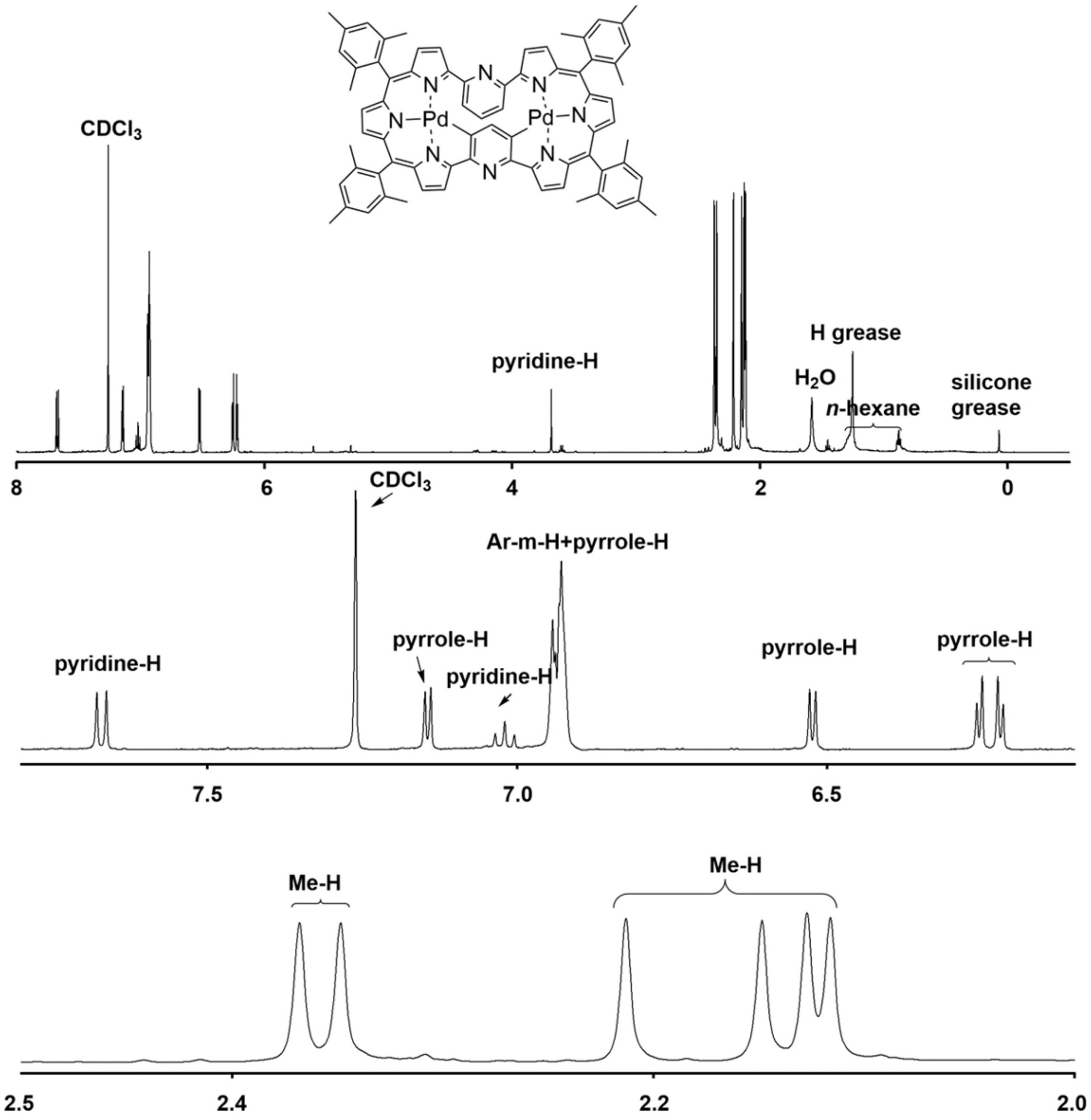

Supplementary Figure 12. ^{1}H NMR spectrum of **16** in $CDCl_3$

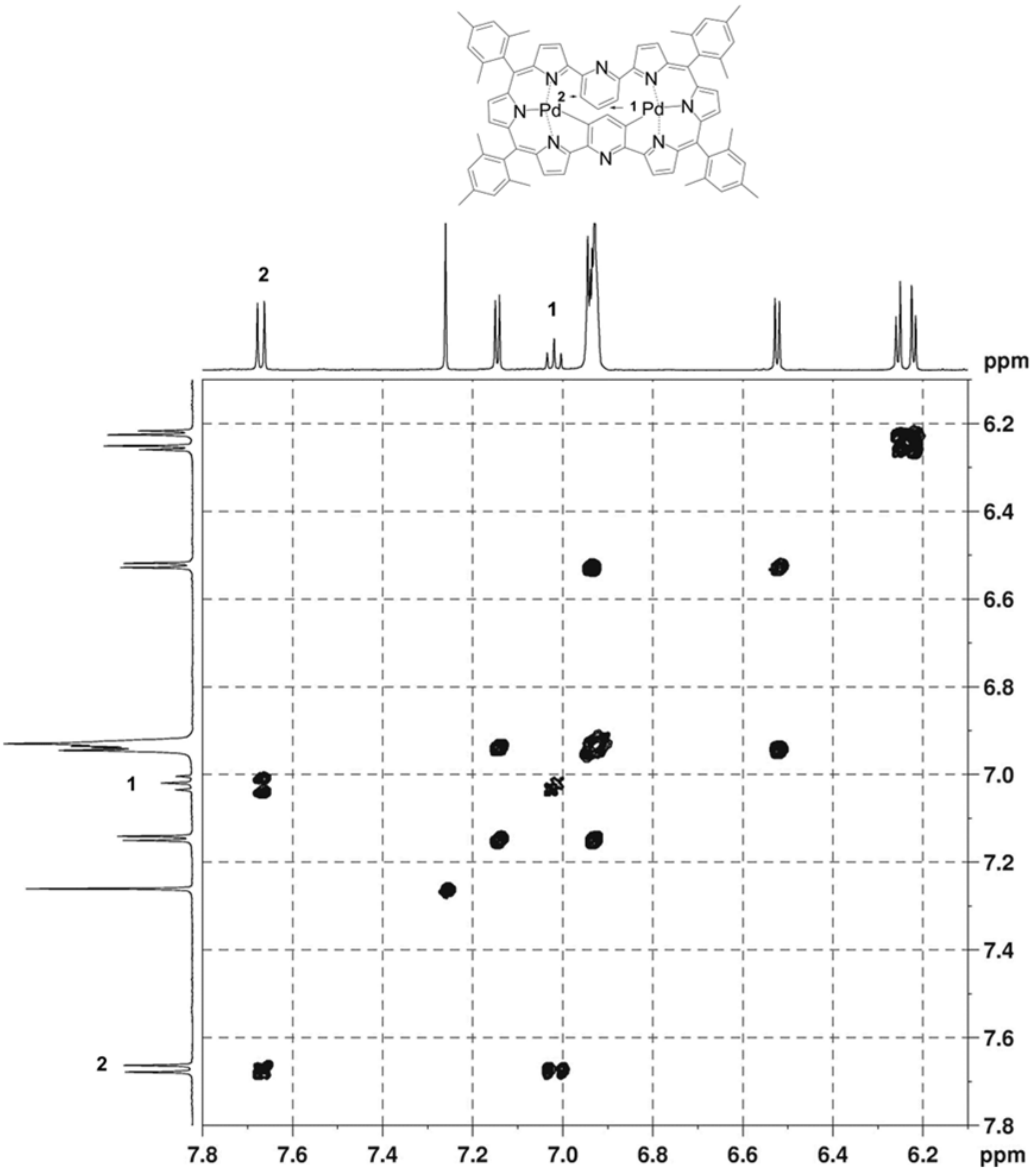

Supplementary Figure 13. COSY spectrum of **16** in $CDCl_3$

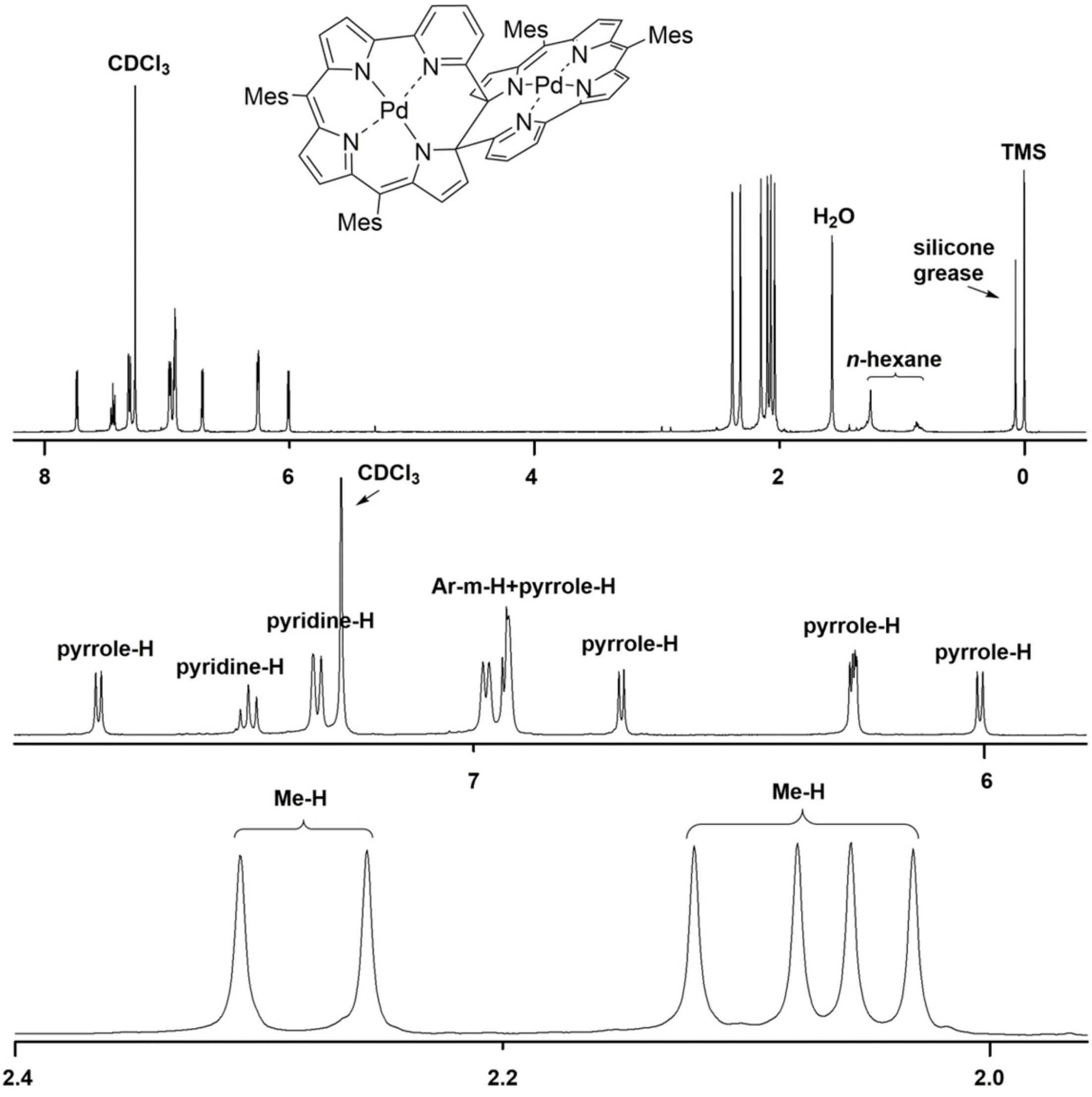

Supplementary Figure 14. ^{1}H NMR spectrum of **17** in $CDCl_3$

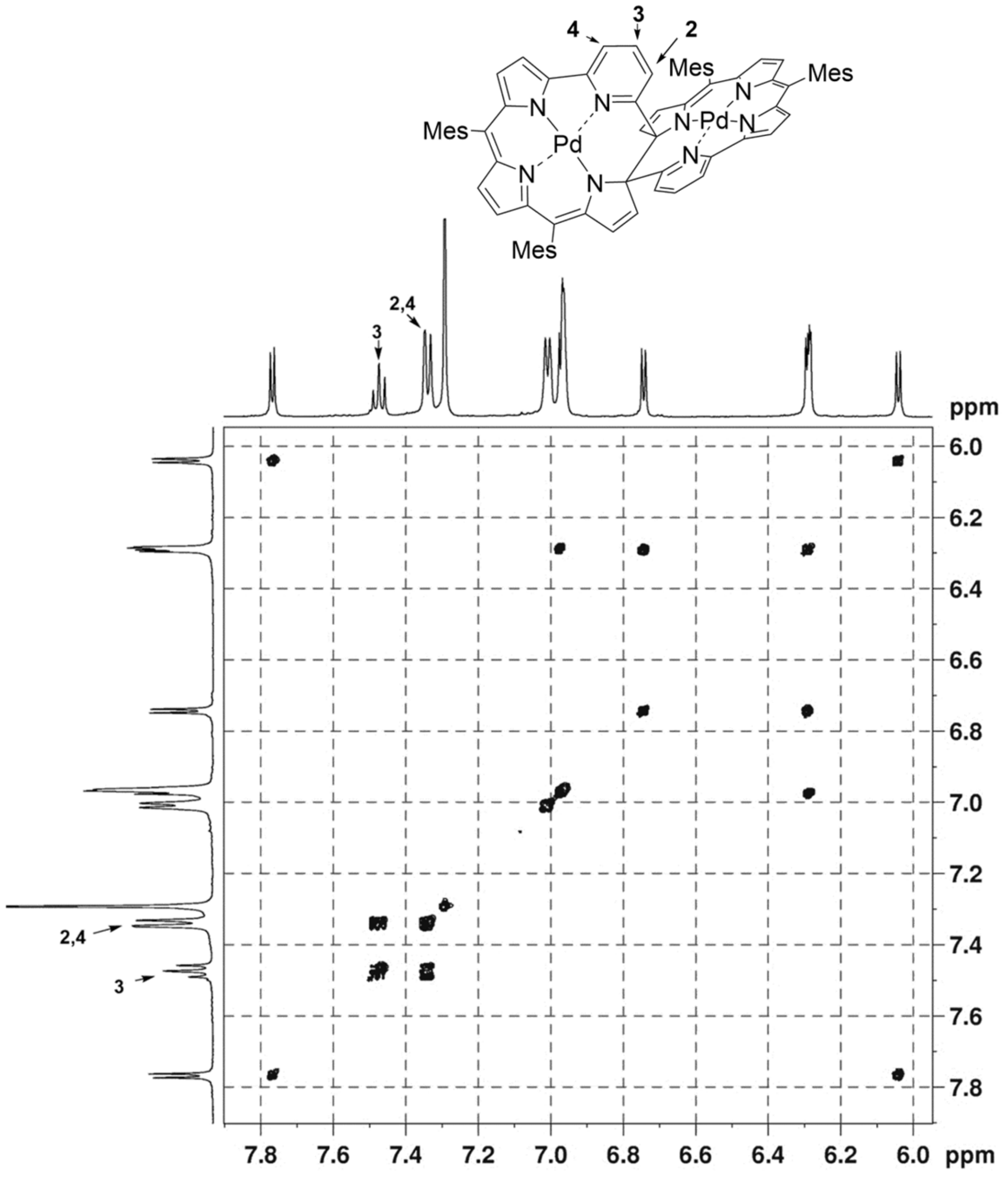

Supplementary Figure 15. COSY spectrum of **17** in $CDCl_3$

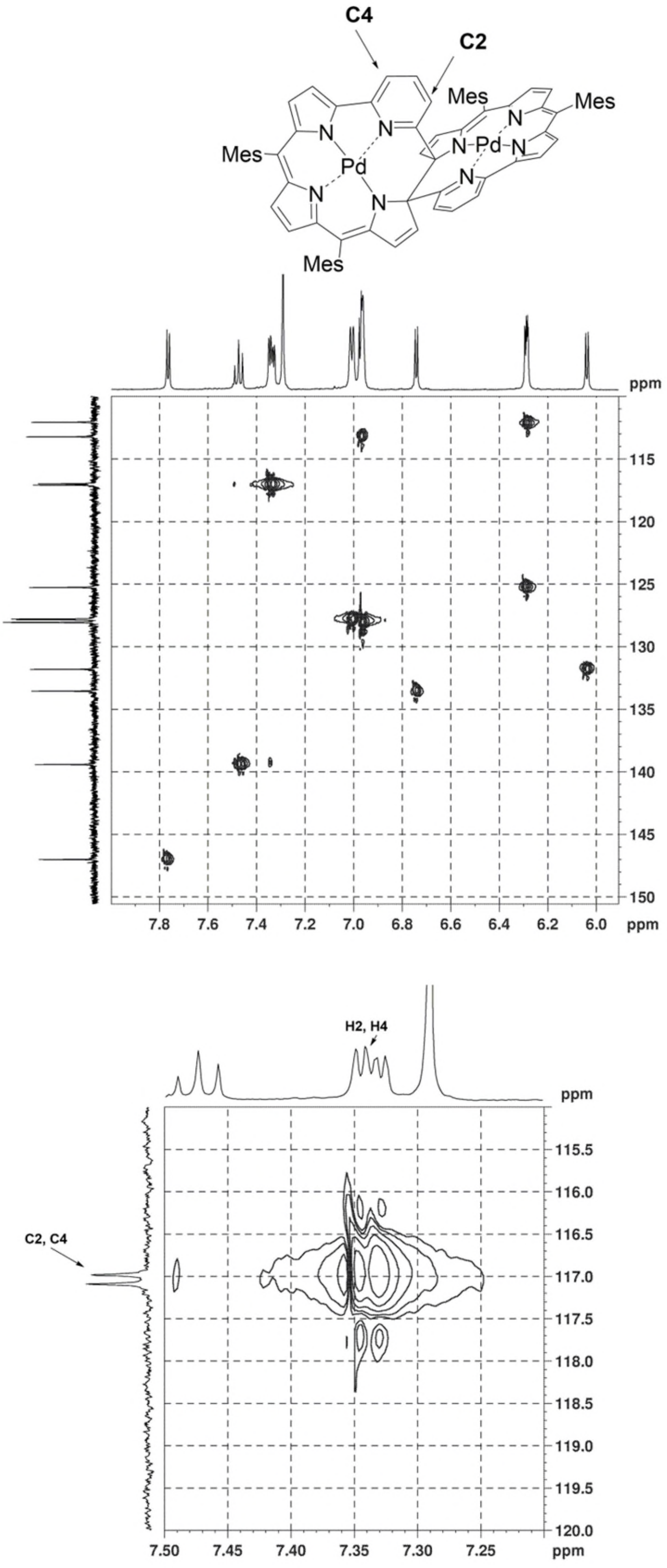

Supplementary Figure 16. HSQC spectrum of **17** in $CDCl_3$

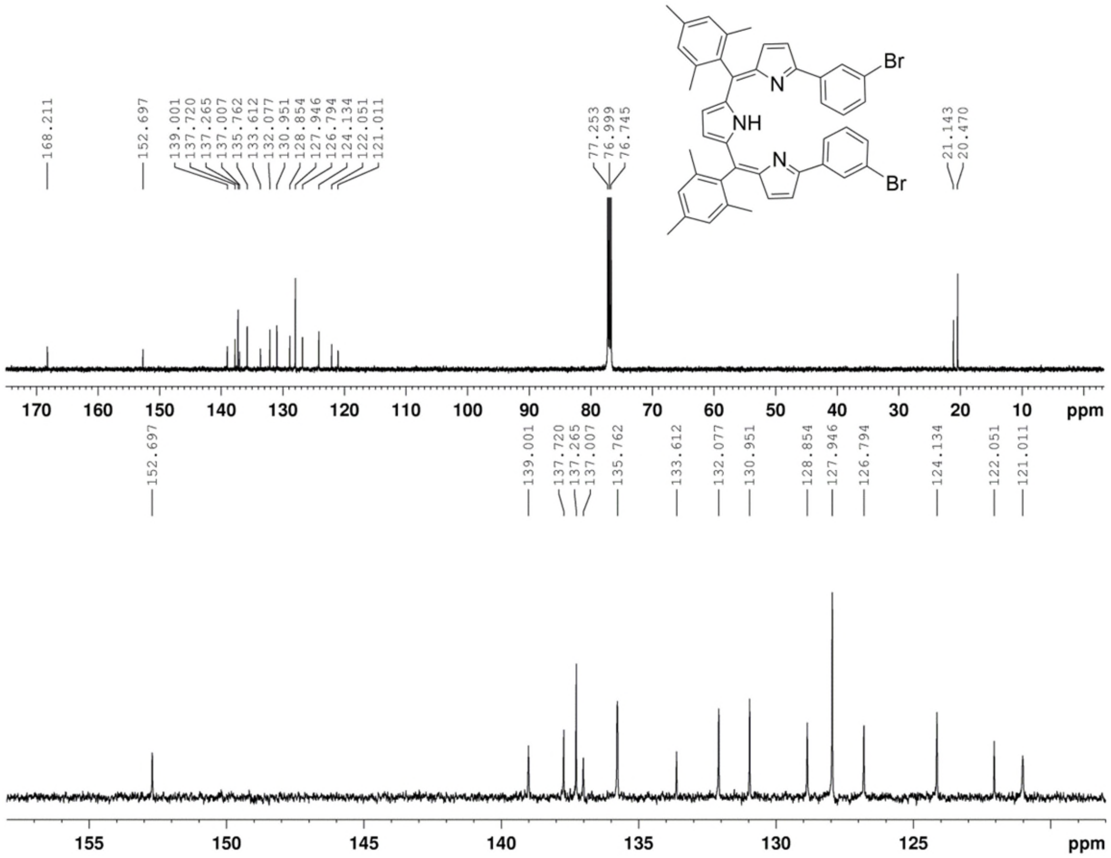

Supplementary Figure 17.^{13}C NMR spectrum of **10** in $CDCl_3$

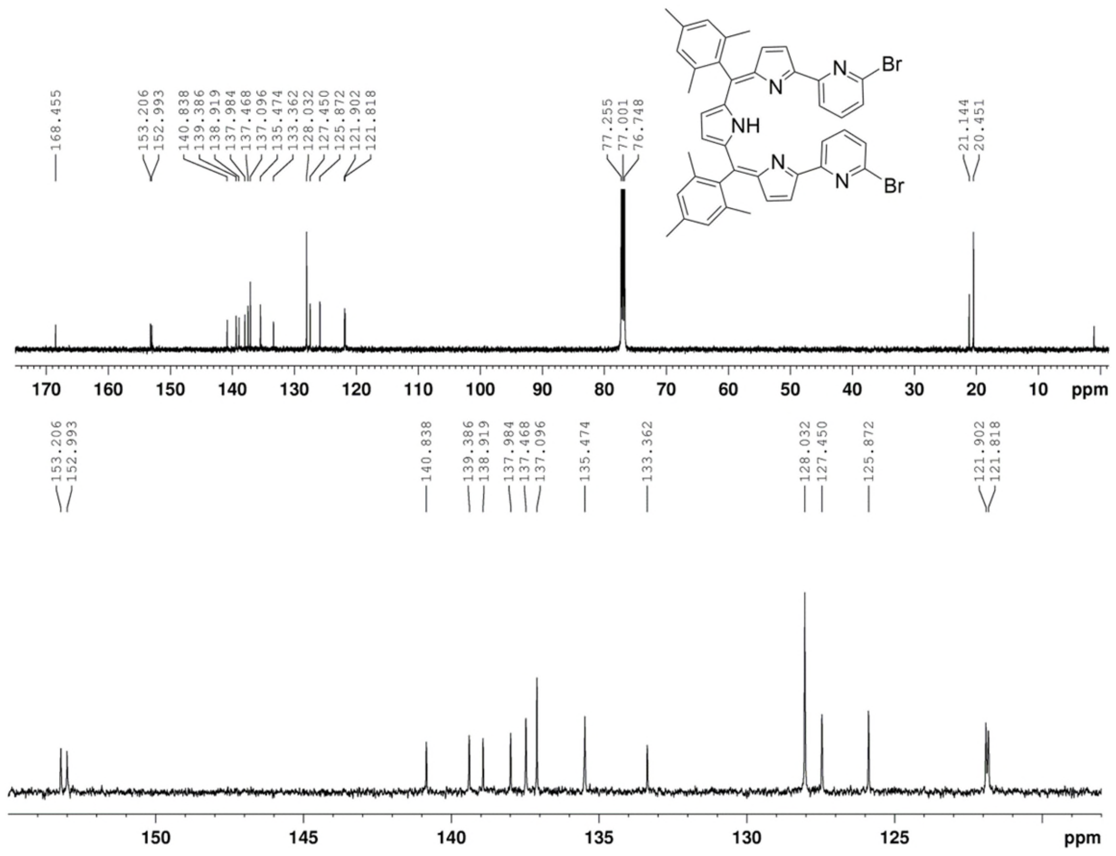

Supplementary Figure 18. ^{13}C NMR spectrum of **11** in $CDCl_3$

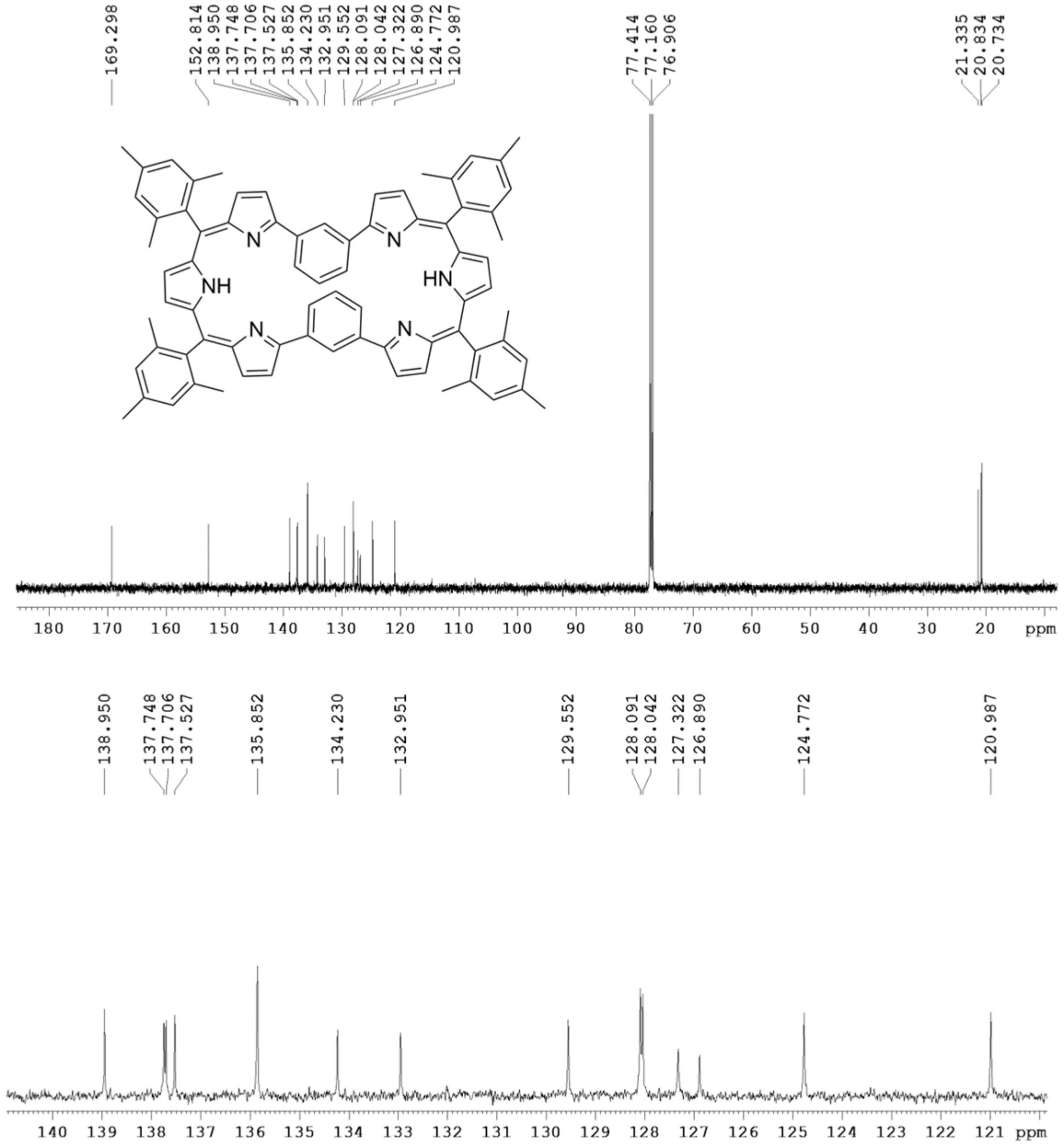

Supplementary Figure 19. ^{13}C NMR spectrum of **12** in $CDCl_3$

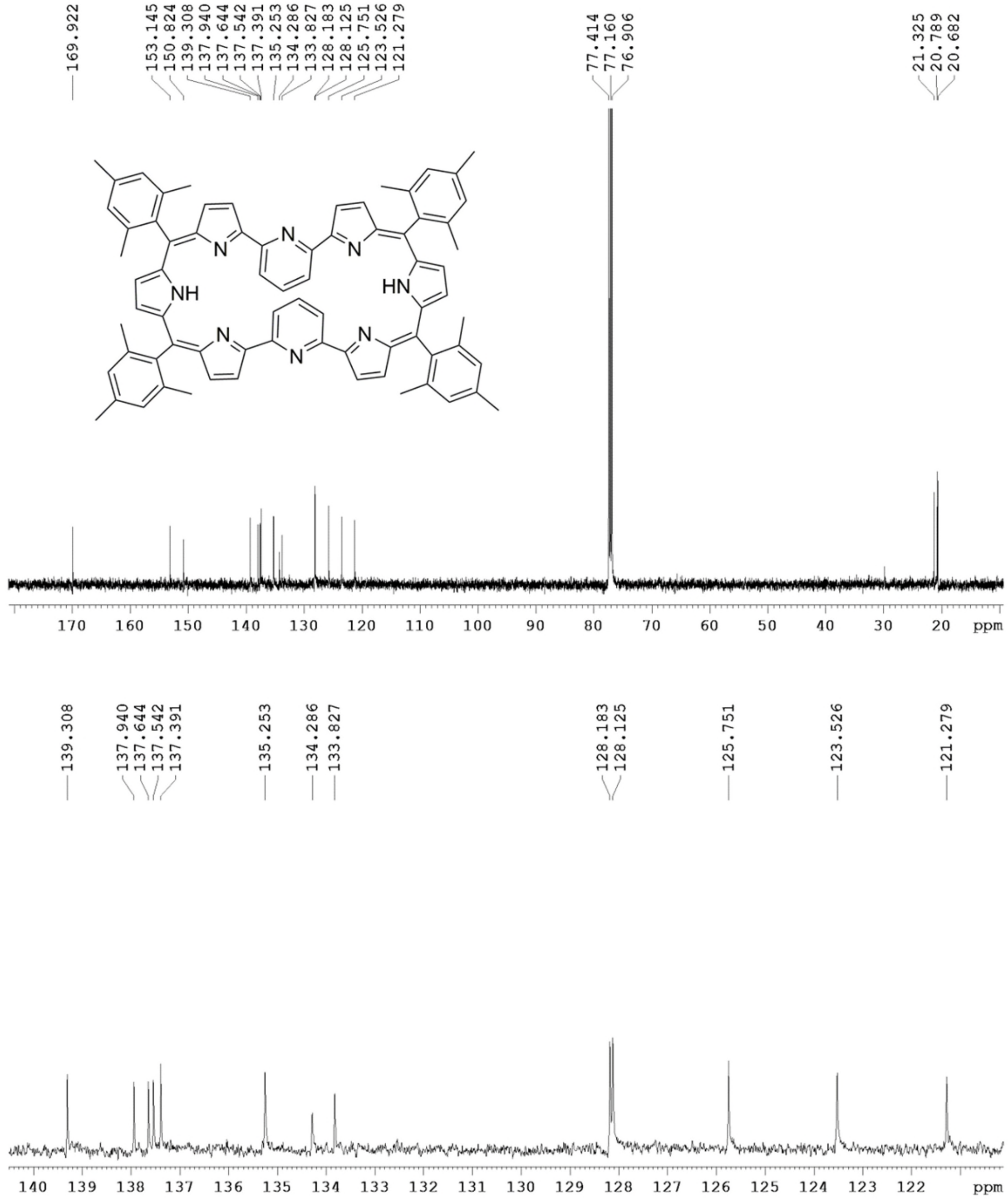

Supplementary Figure 20. ^{13}C NMR spectrum of **13** in $CDCl_3$

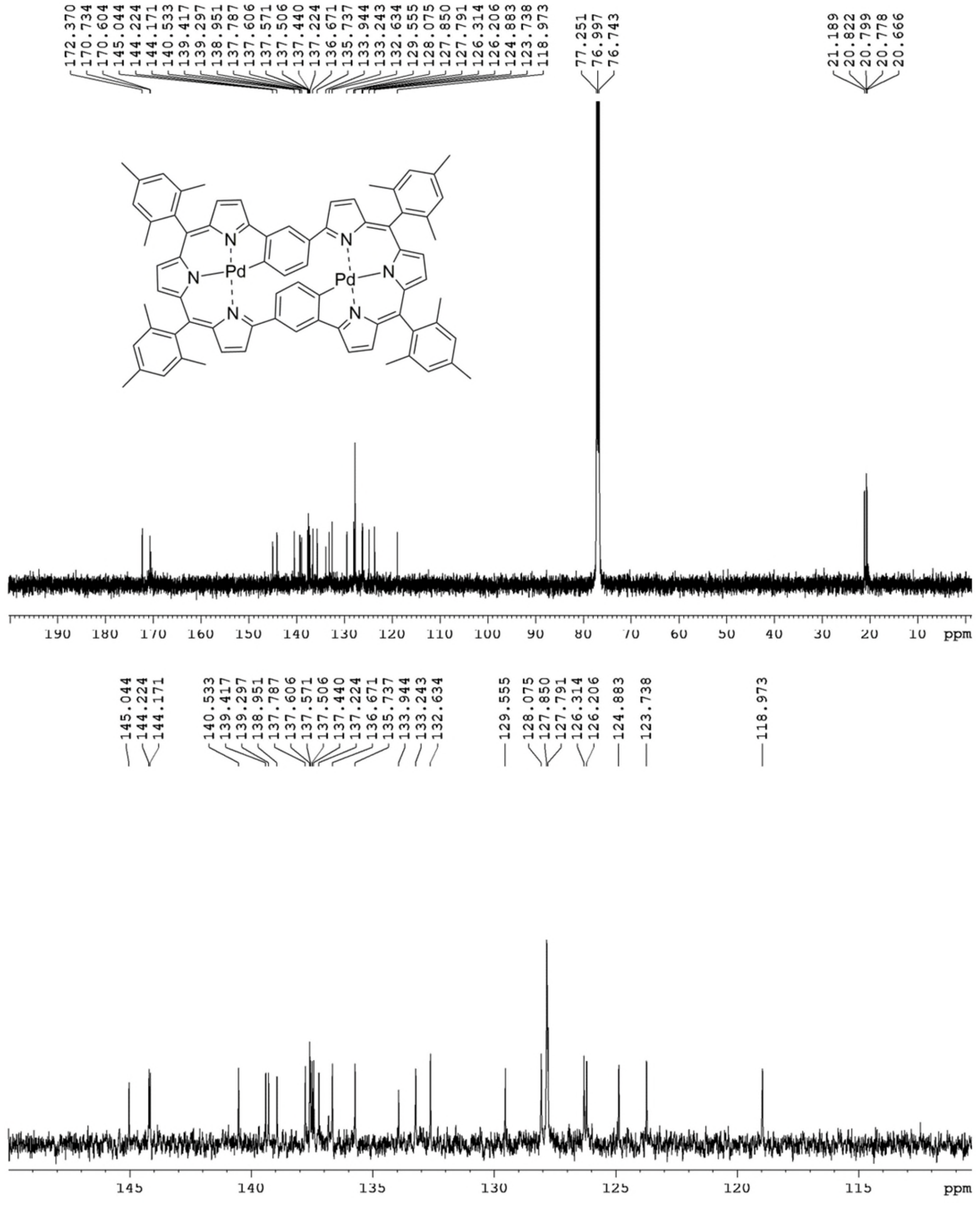

Supplementary Figure 21.^{13}C NMR spectrum of **14** in $CDCl_3$

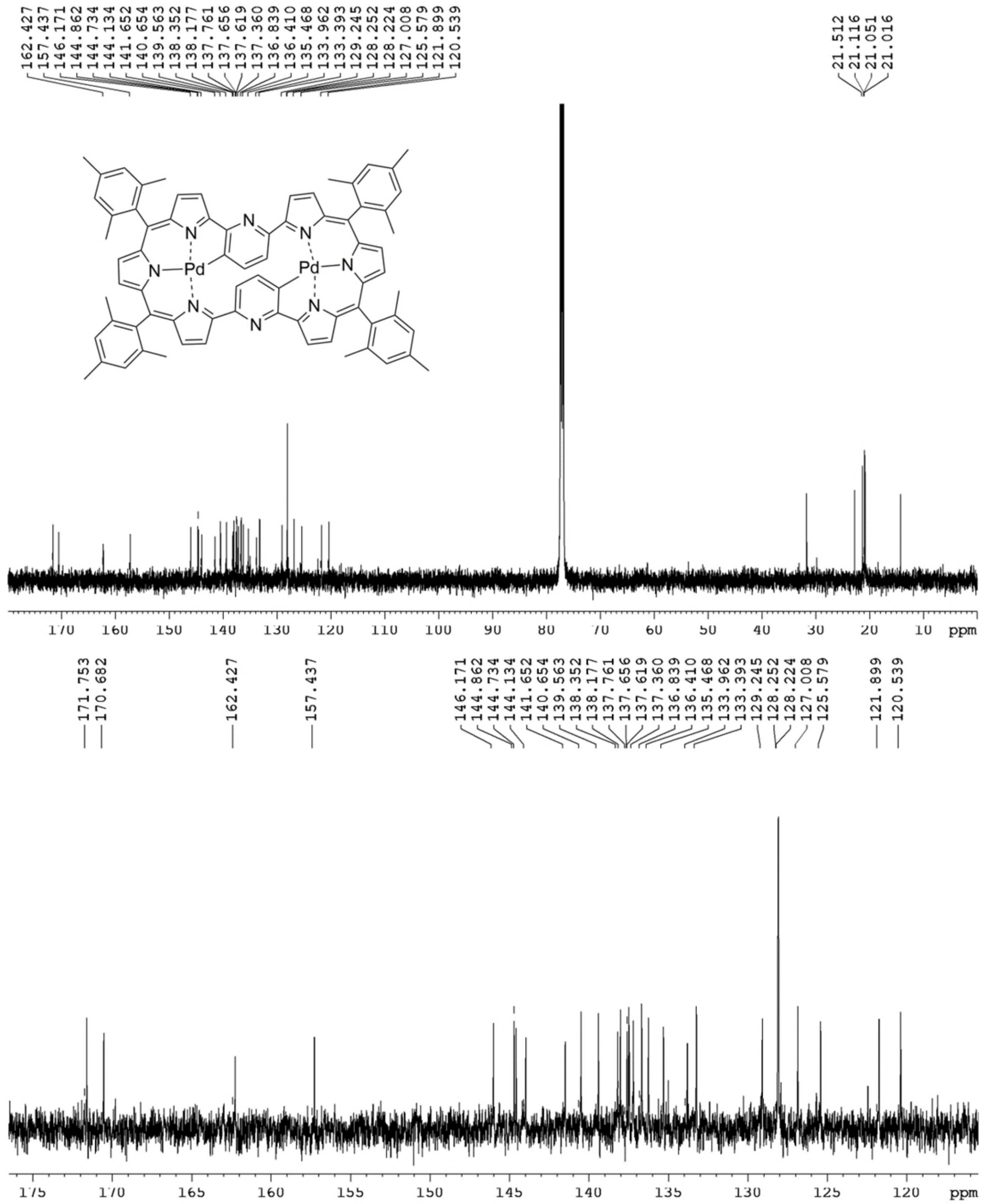

Supplementary Figure 22. ^{13}C NMR spectrum of **15** in $CDCl_3$

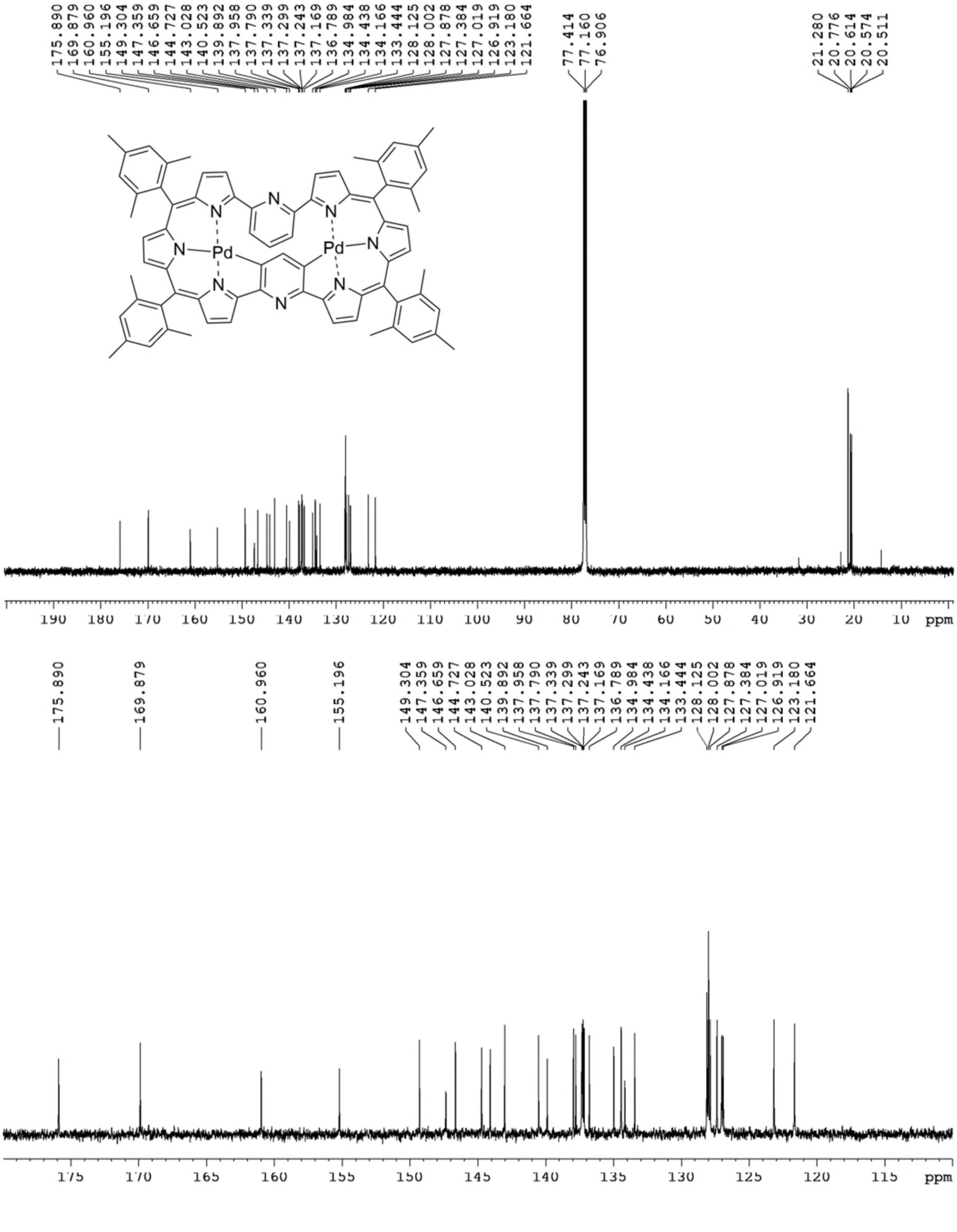

Supplementary Figure 23. ^{13}C NMR spectrum of **16** in $CDCl_3$

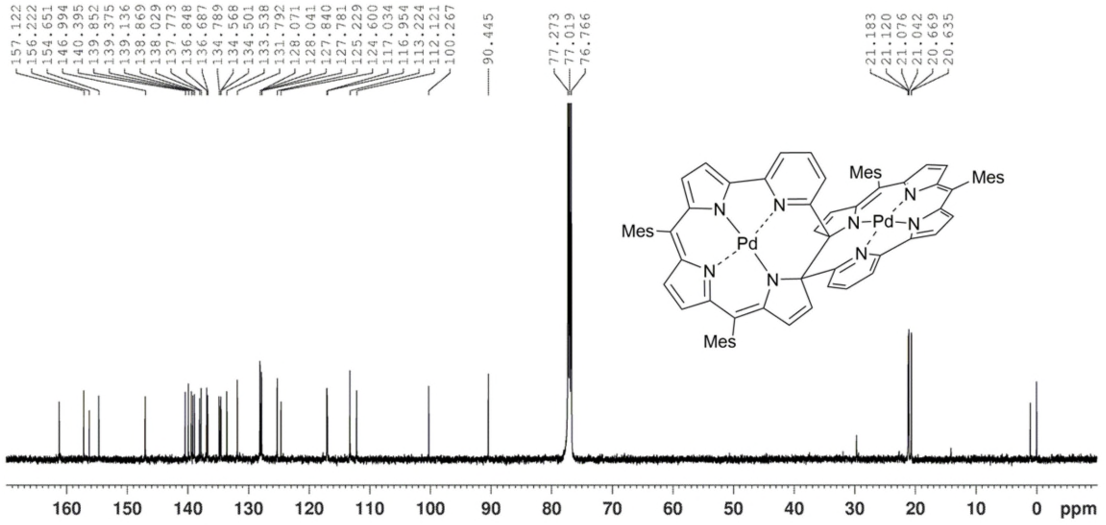

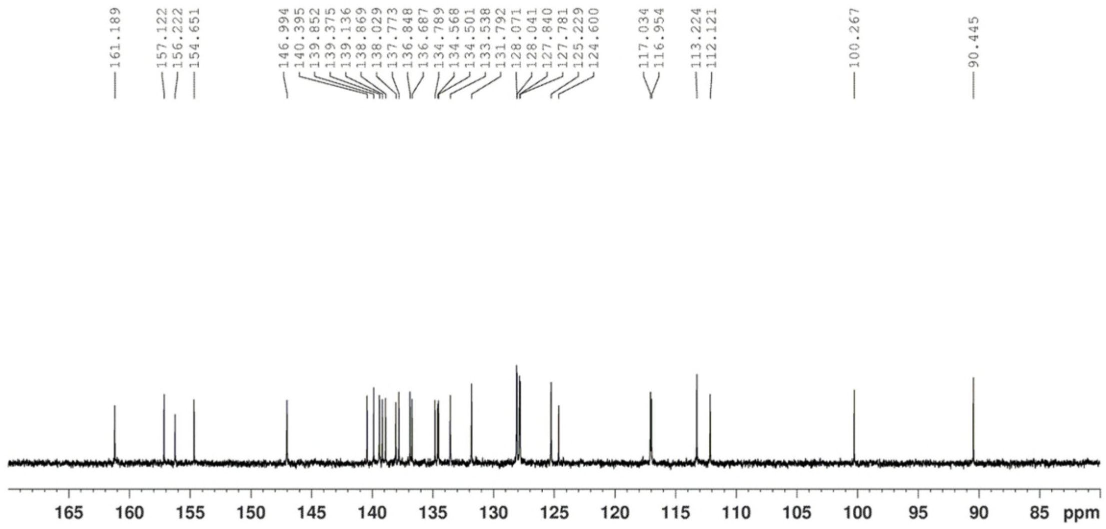

Supplementary Figure 24.^{13}C NMR spectrum of **17** $CDCl_3$

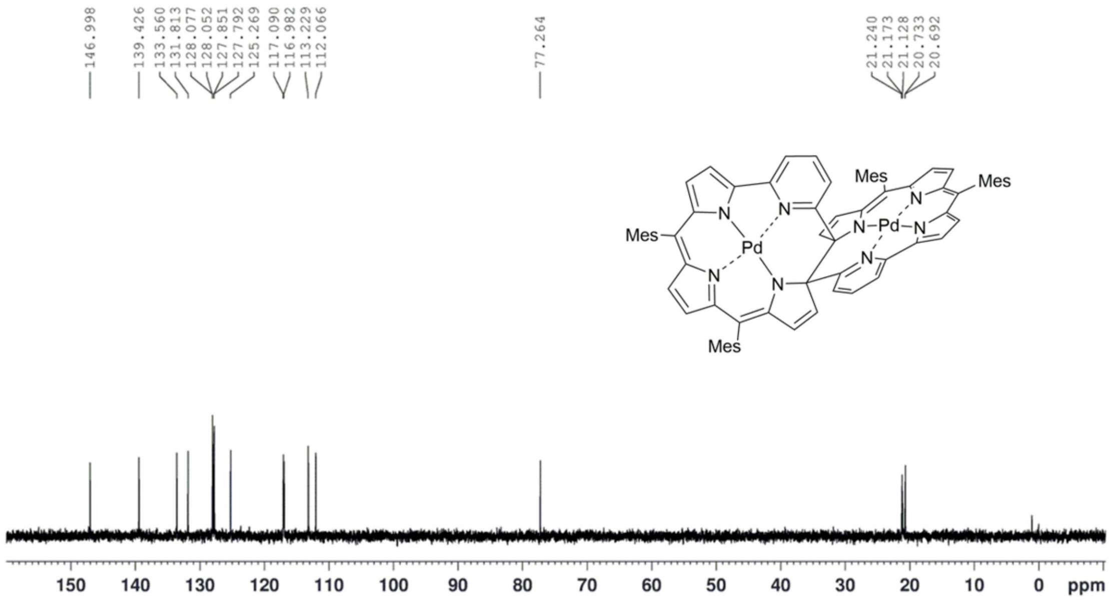

Supplementary Figure 25. ^{13}C NMR Dept135 spectrum of **17** in $CDCl_3$

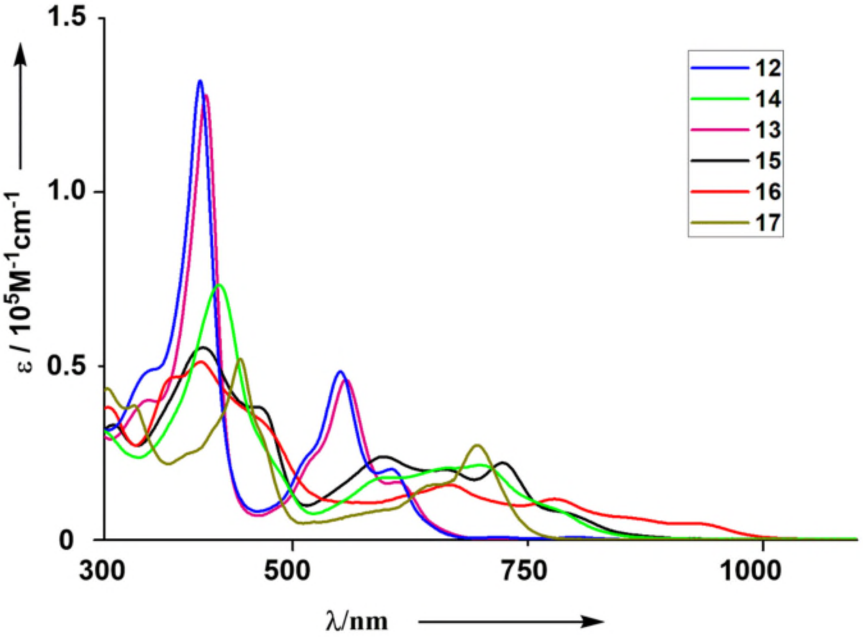

Supplementary Figure 26. UV-Vis-NIR absorption spectra of **12** (blue), 14 (green), **13** (purple), **15** (black), **16** (red), and **17** (khaki). λ, wavelength; ε, Molar extinction coefficient.

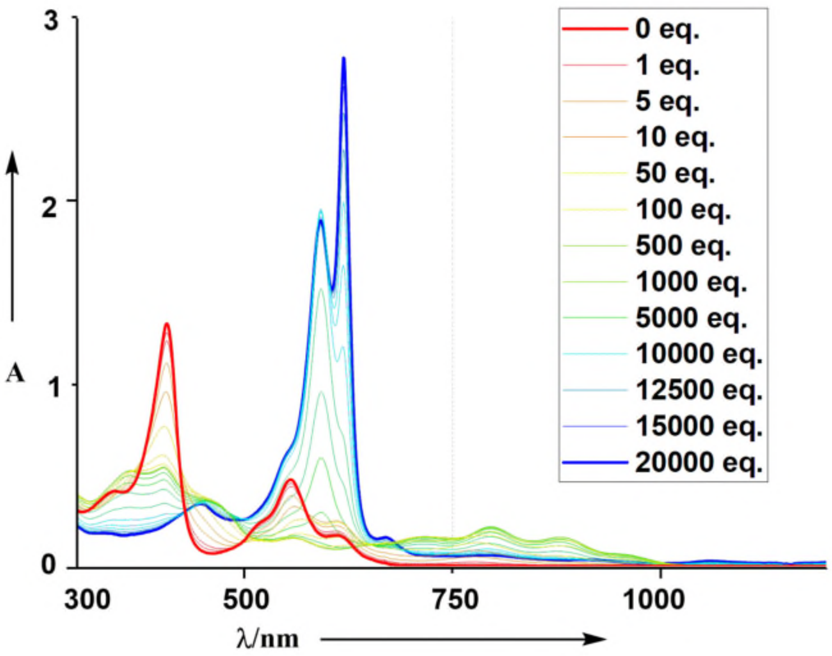

Supplementary Figure 27. UV-Vis-NIR absorption spectra and spectral changes in titration of **13** (red to blue) in CH_2Cl_2 with TFA.

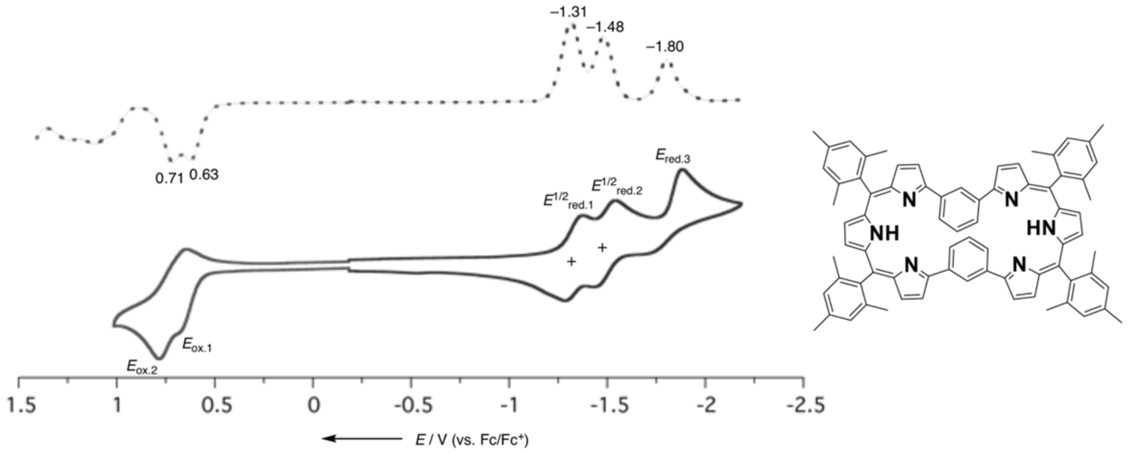

Supplementary Figure 28. Cyclic voltammogram and differential pulse voltammogram of **12**.

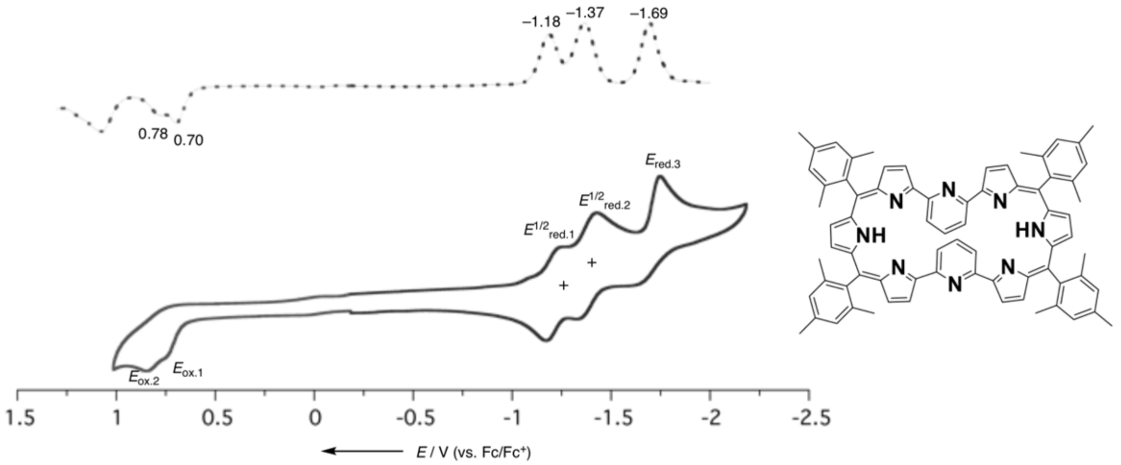

Supplementary Figure 29. Cyclic voltammogram and differential pulse voltammogram of **13**.

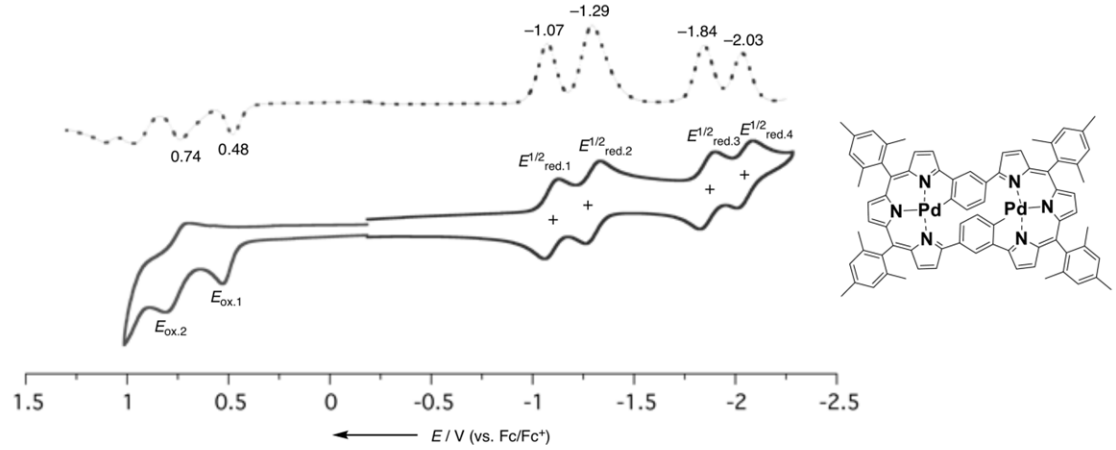

Supplementary Figure 30. Cyclic voltammogram and differential pulse voltammogram of **14**.

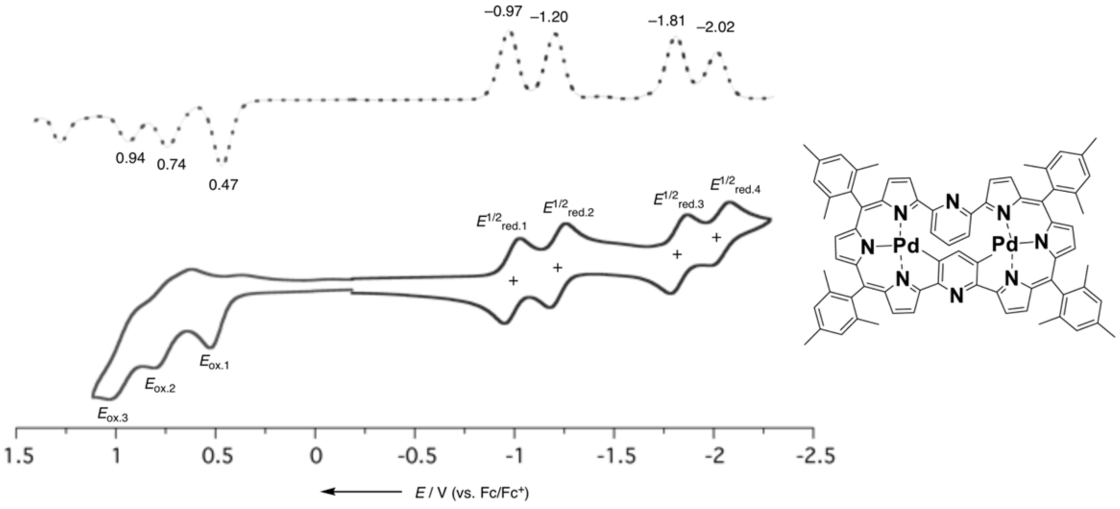

Supplementary Figure 31. Cyclic voltammogram and differential pulse voltammogram of **16**.

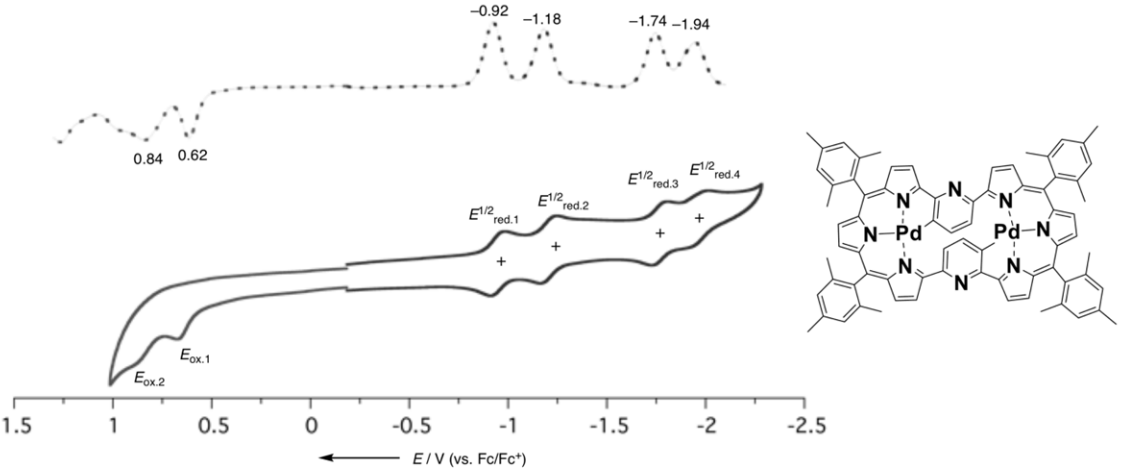

Supplementary Figure 32. Cyclic voltammogram and differential pulse voltammogram of **15**.

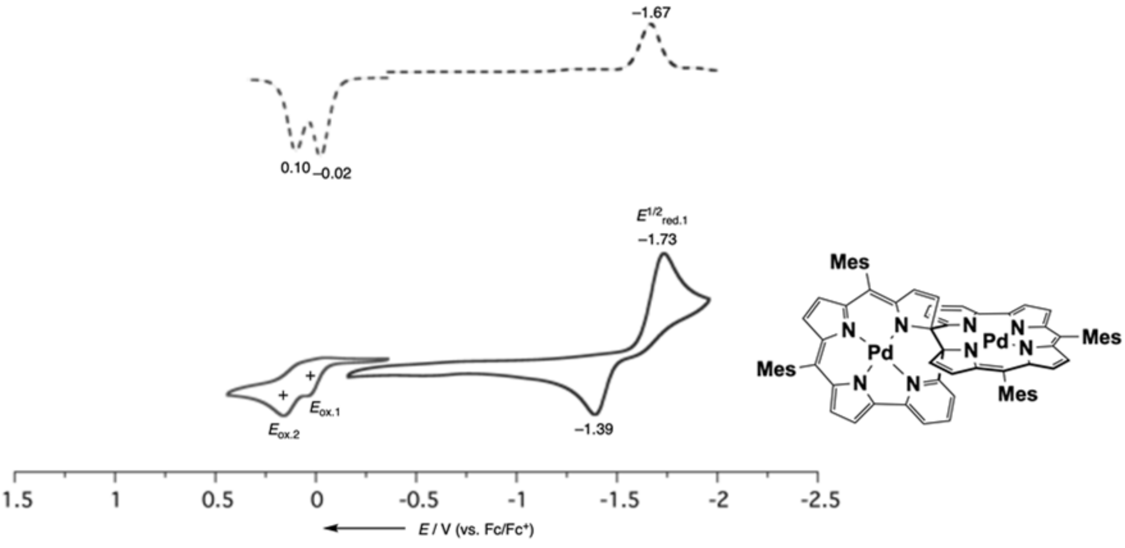

Supplementary Figure 33. Cyclic voltammogram and differential pulse voltammogram of **17**.

a)

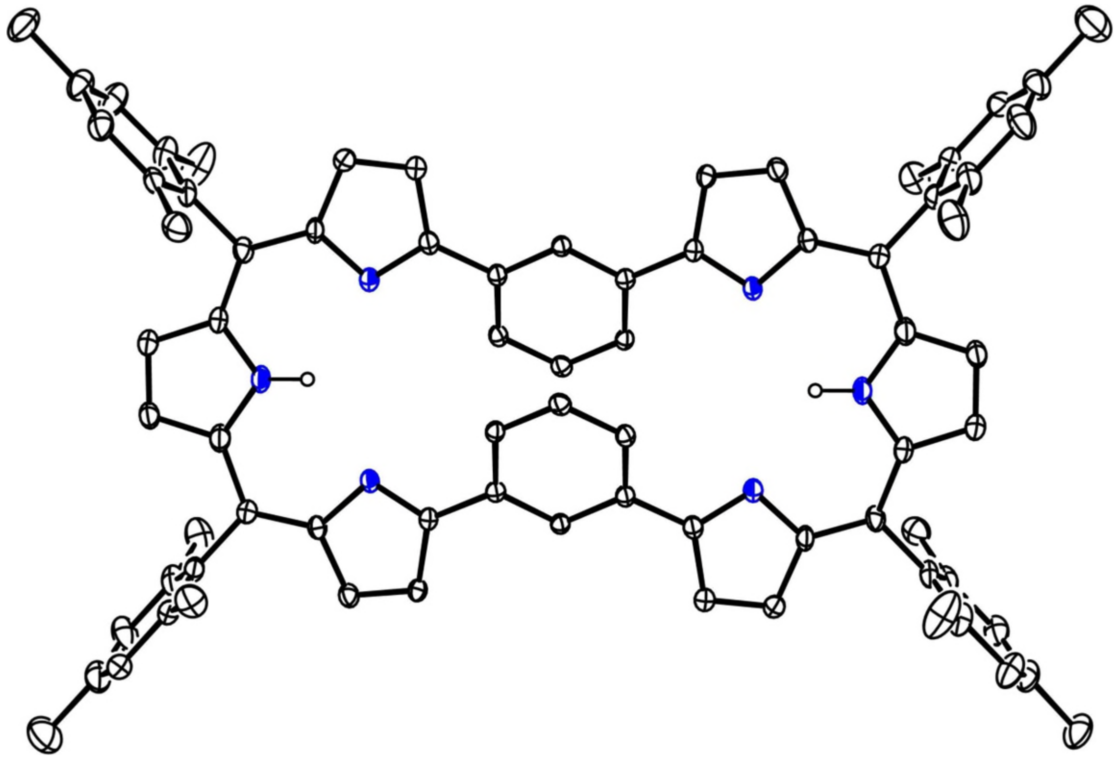

b)

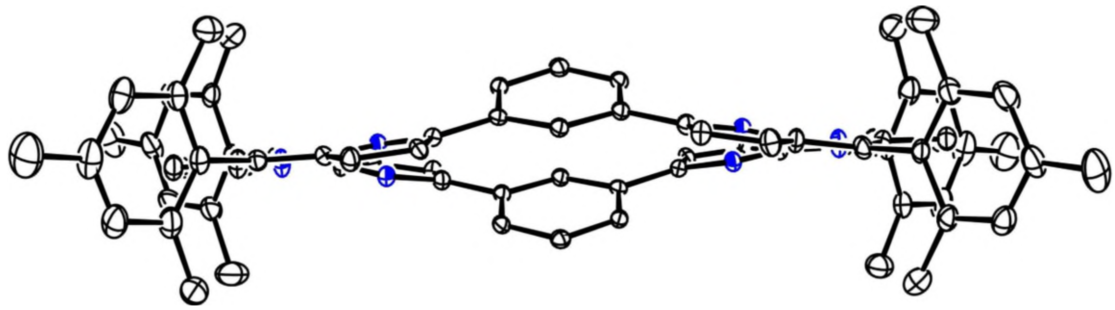

Supplementary Figure 34. X-ray crystal structure of **12**. (a) Top view, (b) side view. The thermal ellipsoids are 30% probability level. Solvent molecules are omitted for clarity. Carbon atom, black ellipsoid; nitrogen atom, blue; hydrogen atom small black ball.

a)

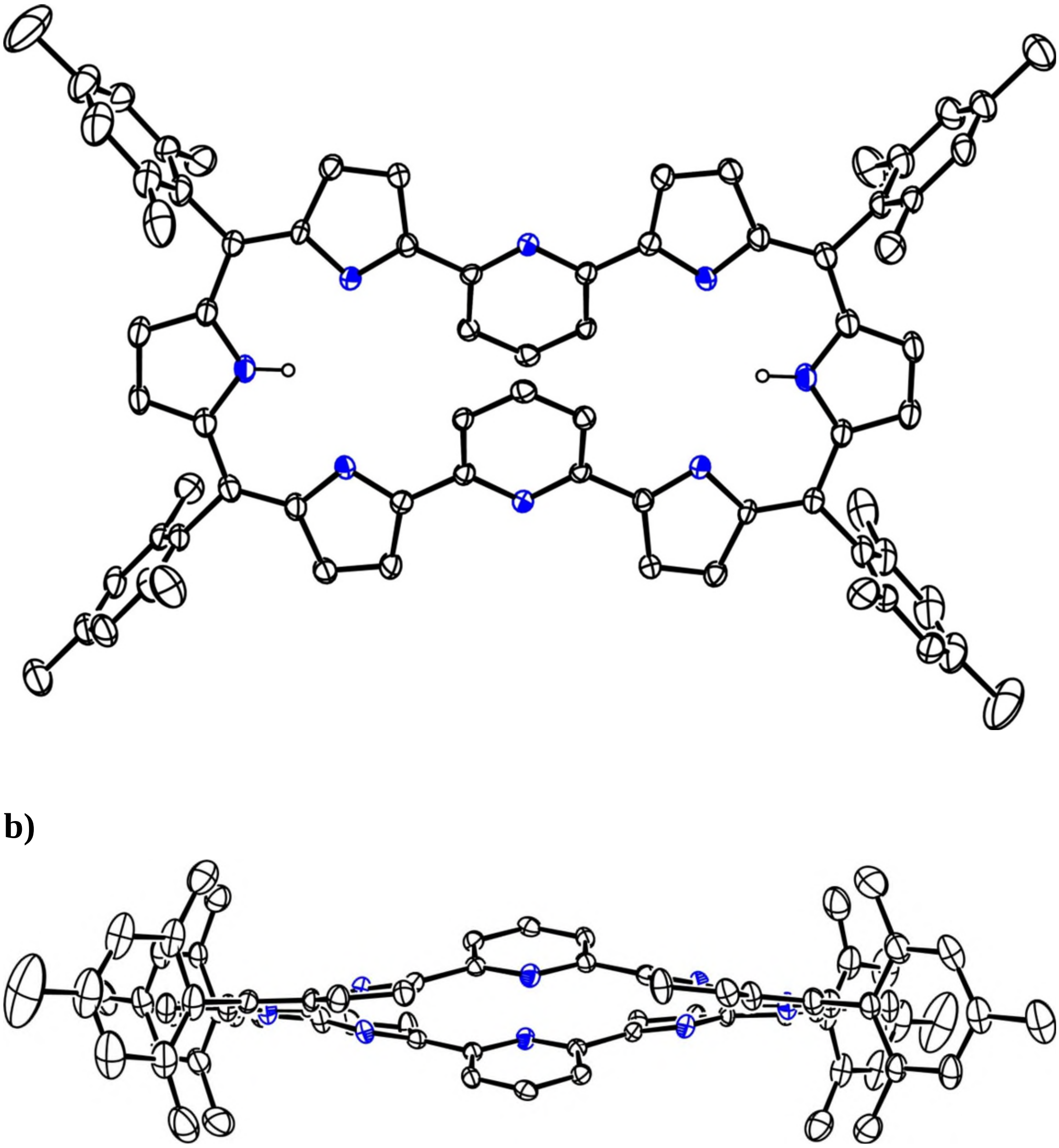

b)

Supplementary Figure 35. X-ray crystal structure of **13**. (a) Top view, (b) side view. The thermal ellipsoids are 30% probability level. Solvent molecules are omitted for clarity. Carbon atom, black ellipsoid; nitrogen atom, blue; hydrogen atom small black ball.

a)

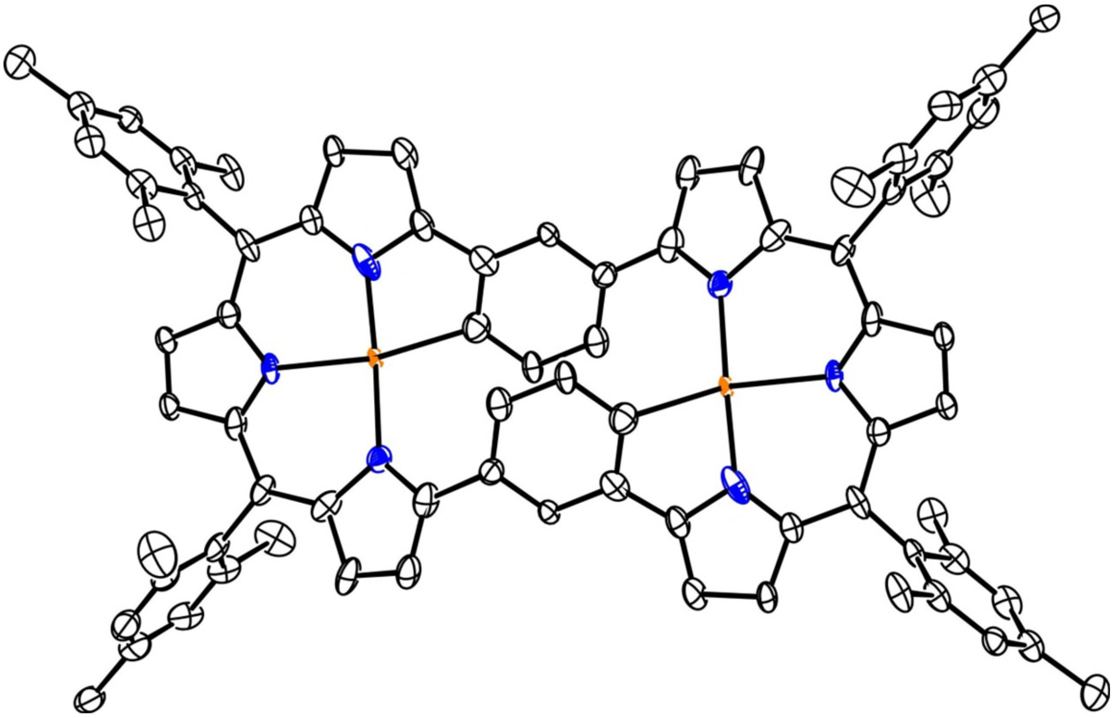

b)

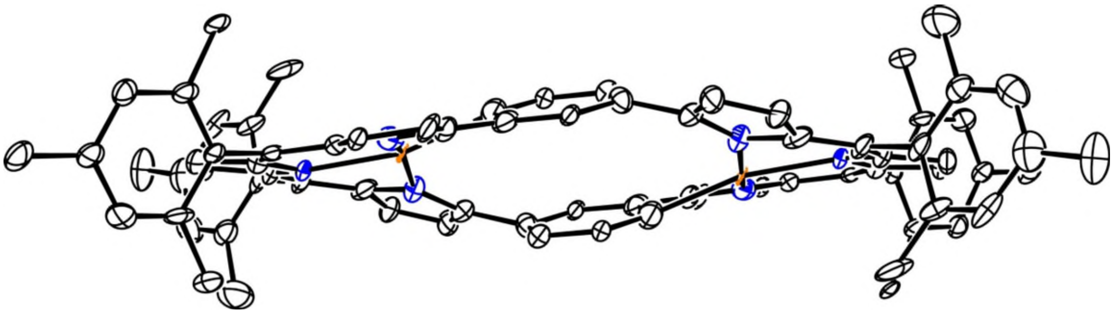

Supplementary Figure 36. X-ray crystal structure of **14**. (a) Top view, (b) side view. The thermal ellipsoids are 30% probability level. Solvent molecules are omitted for clarity. Carbon atom, black ellipsoid; nitrogen atom, blue; palladium atom, orange.

a)

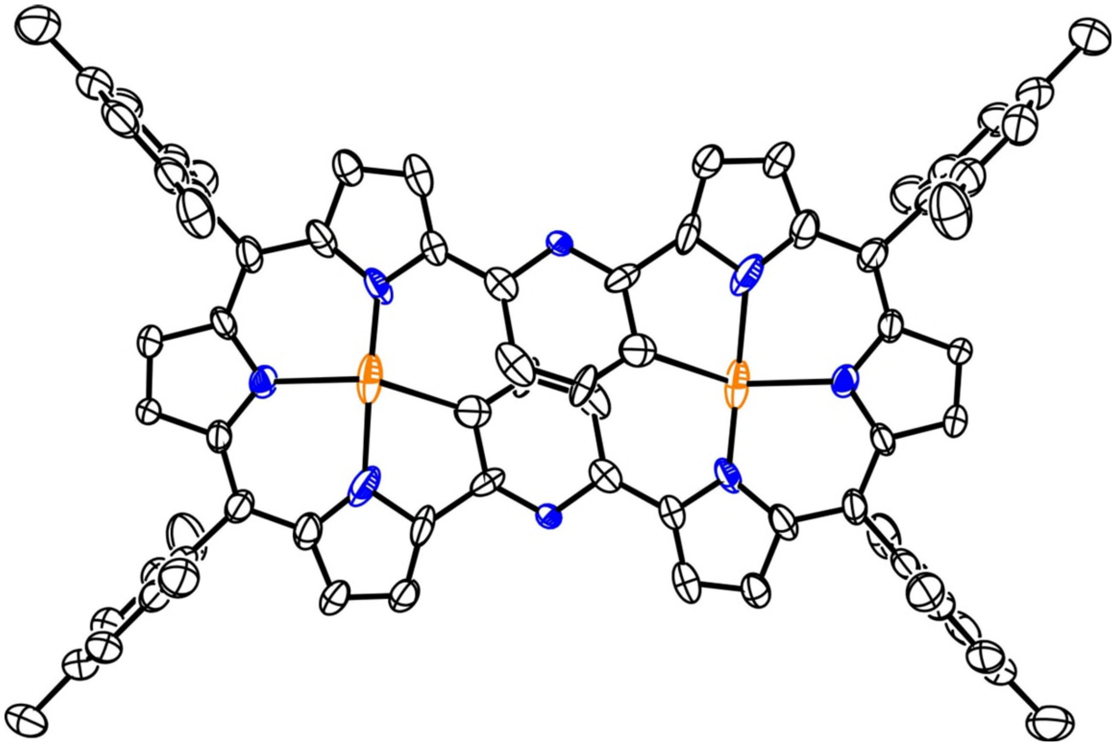

b)

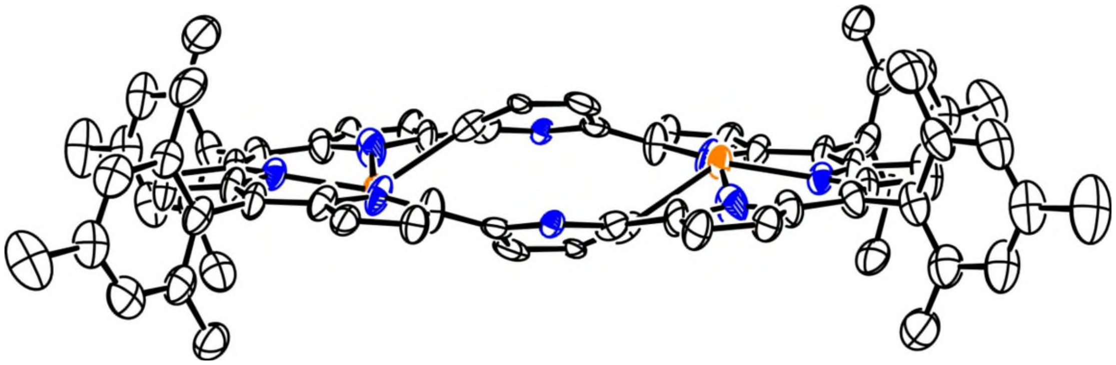

Supplementary Figure 37. X-ray crystal structure of **15**. (a) Top view, (b) side view. The thermal ellipsoids are 30% probability level. Solvent molecules are omitted for clarity. Carbon atom, black ellipsoid; nitrogen atom, blue; palladium atom, orange.

a)

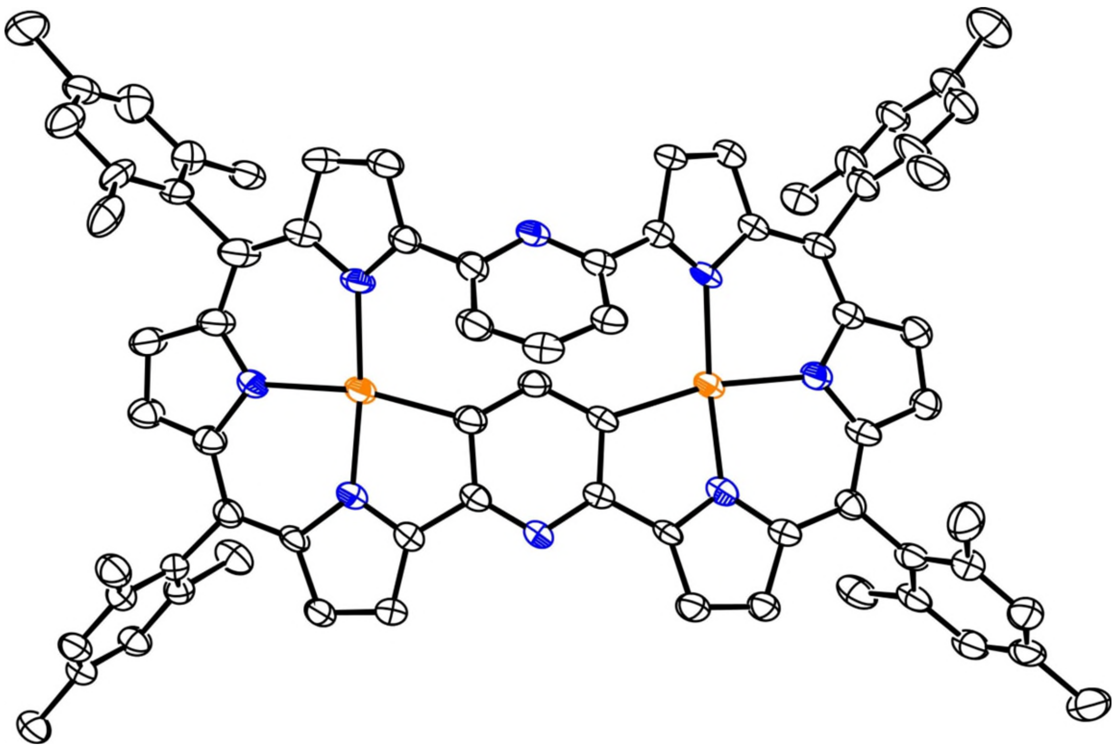

b)

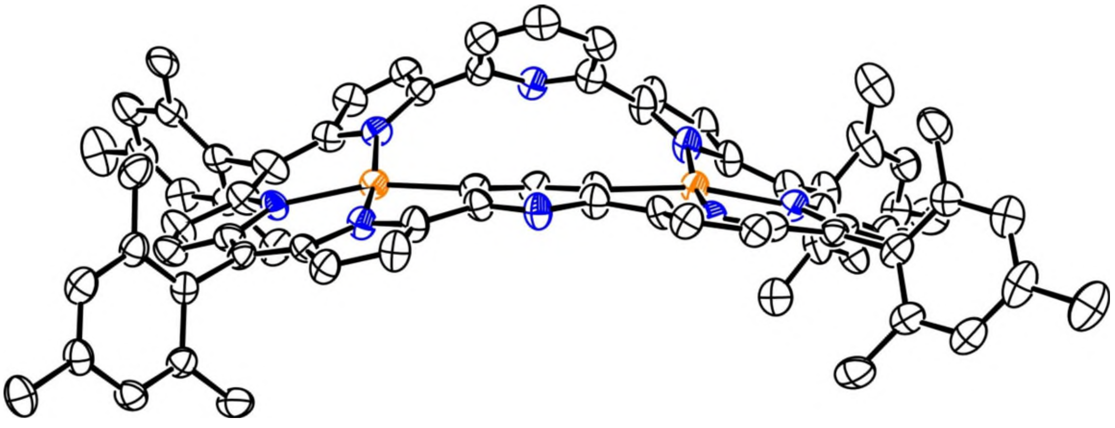

Supplementary Figure 38. X-ray crystal structure of **16**. (a) Top view, (b) side view. The thermal ellipsoids are 30% probability level. Solvent molecules are omitted for clarity. Carbon atom, black ellipsoid; nitrogen atom, blue; palladium atom, orange.

a)

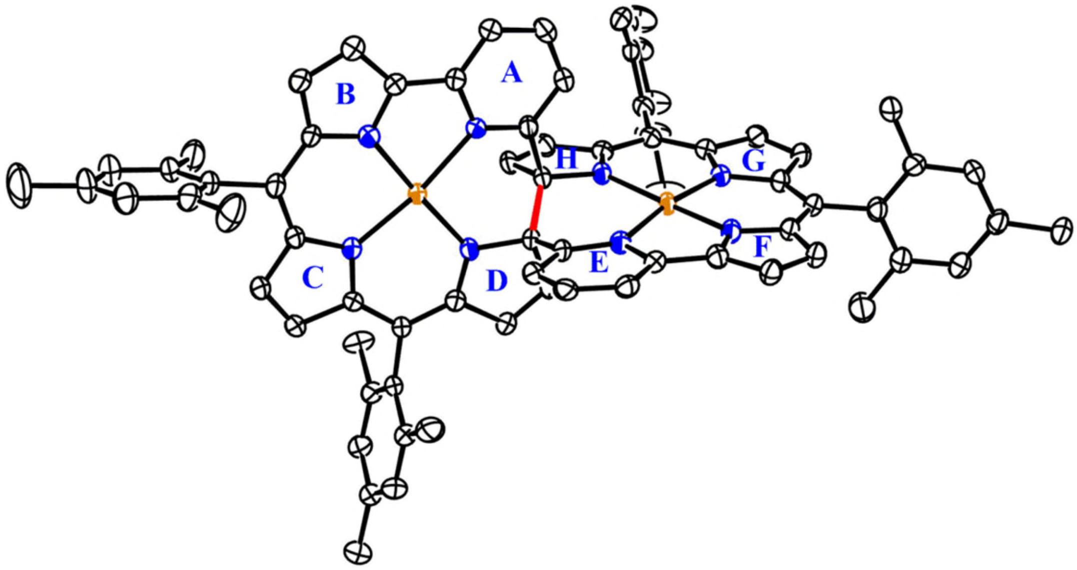

b)

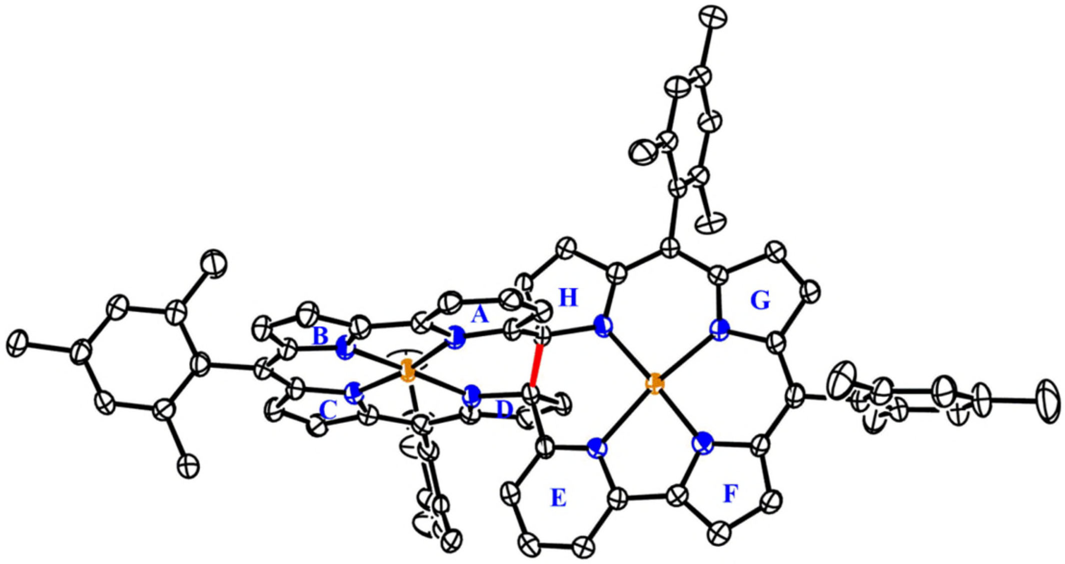

Supplementary Figure 39. X-ray crystal structure of **17**. (a) Top view, (b) side view. The thermal ellipsoids are 30% probability level. Solvent molecules are omitted for clarity. Carbon atom, black ellipsoid; nitrogen atom, blue; palladium atom, orange; C-C bond forming, red.

Supplementary Table 1. Summary of the electrochemical potentials (V) and HOMO–LUMO gaps (eV).

	$E_{ox.3}$	$E_{ox.2}$	$E_{ox.1}$	$E_{red.1}$	$E_{red.2}$	$E_{red.3}$	$E_{red.4}$	ΔE_{HL}
**12**		0.71[a]	0.63[a]	-1.31	-1.48	-1.80[a]		1.94
**13**		0.78[a]	0.70[a]	-1.18	-1.37	-1.69[a]		1.88
**14**		0.74[a]	0.48[a]	-1.07	-1.29	-1.84	-2.03	1.55
**15**		0.84[a]	0.62[a]	-0.92	-1.18	-1.74	-1.94	1.54
**16**	0.94[a]	0.74[a]	0.47[a]	-0.97	-1.20	-1.81	-2.02	1.44
**17**		0.10[a]	-0.02[a]	-1.73				1.71

[a] irreversible peaks [b] $\Delta E_{HL} = e(E_{ox.1}–E_{red.1})$ [eV]

Supplementary Table 2. Crystal data and structure refinement for **12**.

Identification code	p21n-sr_sq	
Empirical formula	C76 H66 N6	
Formula weight	1063.34	
Temperature	100.00(10) K	
Wavelength	1.54178 Å	
Crystal system	Monoclinic	
Space group	P 1 21/n 1	
Unit cell dimensions	a = 14.4114(2) Å	α= 90°.
	b = 17.7669(2) Å	β= 103.1050(10)°.
	c = 14.6531(2) Å	γ = 90°.
Volume	3654.15(8) $Å^3$	
Z	2	
Density (calculated)	0.966 Mg/m^3	
Absorption coefficient	0.433 mm^{-1}	
F(000)	1128	
Crystal size	0.2 x 0.2 x 0.05 mm^3	
Theta range for data collection	3.884 to 66.588°.	
Index ranges	-17<=h<=17, -21<=k<=19, -17<=l<=17	
Reflections collected	50030	
Independent reflections	6445 [R(int) = 0.0349]	
Completeness to theta = 66.588°	99.8 %	
Absorption correction	Semi-empirical from equivalents	
Max. and min. transmission	1.00000 and 0.69482	
Refinement method	Full-matrix least-squares on F^2	
Data / restraints / parameters	6445 / 504 / 710	
Goodness-of-fit on F^2	1.072	
Final R indices [I>2sigma(I)]	R1 = 0.0621, wR2 = 0.1609	
R indices (all data)	R1 = 0.0673, wR2 = 0.1651	
Extinction coefficient	n/a	
Largest diff. peak and hole	0.881 and -0.243 e.$Å^{-3}$	
CCDC number	1959823	

Supplementary Table 3. Crystal data and structure refinement for **13**.

Identification code	exp_655_sq	
Empirical formula	C74 H64 N8	
Formula weight	1065.33	
Temperature	99.99(10) K	
Wavelength	1.54184 Å	
Crystal system	Monoclinic	
Space group	P 1 21/n 1	
Unit cell dimensions	a = 14.2250(8) Å	α= 90°.
	b = 17.6723(8) Å	β= 103.204(6)°.
	c = 14.5241(10) Å	γ = 90°.
Volume	3554.7(4) Å^3	
Z	2	
Density (calculated)	0.995 Mg/m^3	
Absorption coefficient	0.455 mm^{-1}	
F(000)	1128	
Crystal size	0.3 x 0.3 x 0.2 mm^3	
Theta range for data collection	4.004 to 66.599°.	
Index ranges	-16<=h<=16, -16<=k<=21, -17<=l<=17	
Reflections collected	44464	
Independent reflections	6260 [R(int) = 0.0682]	
Completeness to theta = 66.599°	100.0 %	
Absorption correction	Semi-empirical from equivalents	
Max. and min. transmission	1.00000 and 0.42476	
Refinement method	Full-matrix least-squares on F^2	
Data / restraints / parameters	6260 / 809 / 753	
Goodness-of-fit on F^2	1.058	
Final R indices [I>2sigma(I)]	R1 = 0.0771, wR2 = 0.2122	
R indices (all data)	R1 = 0.0944, wR2 = 0.2253	
Extinction coefficient	n/a	
Largest diff. peak and hole	0.345 and -0.264 e.Å^{-3}	
CCDC number	1959827	

Supplementary Table 4. Crystal data and structure refinement for **14**

Identification code	exp_377-sr	
Empirical formula	C76 H62 N6 Pd2	
Formula weight	1272.11	
Temperature	100.01(10) K	
Wavelength	1.54184 Å	
Crystal system	Triclinic	
Space group	P-1	
Unit cell dimensions	a = 13.7108(3) Å	α= 88.6924(12)°.
	b = 14.9407(3) Å	β= 82.8216(13)°.
	c = 20.4052(2) Å	γ = 73.1001(18)°.
Volume	3967.72(13) $Å^3$	
Z	2	
Density (calculated)	1.065 Mg/m^3	
Absorption coefficient	3.947 mm^{-1}	
F(000)	1304	
Crystal size	0.3 x 0.2 x 0.1 mm^3	
Theta range for data collection	3.803 to 66.592°.	
Index ranges	-16<=h<=16, -18<=k<=18, -25<=l<=25	
Reflections collected	52295	
Independent reflections	13998 [R(int) = 0.1049]	
Completeness to theta = 66.592°	99.8 %	
Absorption correction	Semi-empirical from equivalents	
Max. and min. transmission	1.00000 and 0.19774	
Refinement method	Full-matrix least-squares on F^2	
Data / restraints / parameters	13998 / 1269 / 1080	
Goodness-of-fit on F^2	1.262	
Final R indices [I>2sigma(I)]	R1 = 0.1049, wR2 = 0.3002	
R indices (all data)	R1 = 0.1235, wR2 = 0.3184	
Extinction coefficient	n/a	
Largest diff. peak and hole	4.016 and -1.589 e.$Å^{-3}$	
CCDC number	1959825	

Supplementary Table 5. Crystal data and structure refinement for **15**.

Identification code	exp_556_sq	
Empirical formula	C74 H60 N8 Pd2	
Formula weight	1274.10	
Temperature	100.01(10) K	
Wavelength	1.54184 Å	
Crystal system	Monoclinic	
Space group	P 1 21/n 1	
Unit cell dimensions	a = 14.3157(6) Å	α= 90°.
	b = 17.9440(5) Å	β= 105.822(5)°.
	c = 14.3530(7) Å	γ = 90°.
Volume	3547.3(3) Å^3	
Z	2	
Density (calculated)	1.193 Mg/m^3	
Absorption coefficient	4.425 mm^{-1}	
F(000)	1304	
Crystal size	0.2 x 0.2 x 0.2 mm^3	
Theta range for data collection	4.585 to 66.593°.	
Index ranges	-17<=h<=16, -21<=k<=21, -17<=l<=17	
Reflections collected	46327	
Independent reflections	6266 [R(int) = 0.0565]	
Completeness to theta = 66.593°	99.9 %	
Absorption correction	Semi-empirical from equivalents	
Max. and min. transmission	1.00000 and 0.26456	
Refinement method	Full-matrix least-squares on F^2	
Data / restraints / parameters	6266 / 860 / 698	
Goodness-of-fit on F^2	1.022	
Final R indices [I>2sigma(I)]	R1 = 0.1047, wR2 = 0.2680	
R indices (all data)	R1 = 0.1198, wR2 = 0.2818	
Extinction coefficient	n/a	
Largest diff. peak and hole	1.481 and -1.109 e.Å^{-3}	
CCDC number	1959828	

Supplementary Table 6. Crystal data and structure refinement for **16**.

Identification code	exp_442-sr	
Empirical formula	C74 H60 N8 Pd2	
Formula weight	1274.10	
Temperature	100.00(10) K	
Wavelength	1.54184 Å	
Crystal system	Monoclinic	
Space group	C 1 2/c 1	
Unit cell dimensions	a = 24.2234(13) Å	α= 90°.
	b = 13.7751(6) Å	β= 97.136(4)°.
	c = 42.7342(15) Å	γ = 90°.
Volume	14149.1(11) Å^3	
Z	8	
Density (calculated)	1.196 Mg/m^3	
Absorption coefficient	4.437 mm^{-1}	
F(000)	5216	
Crystal size	0.2 x 0.2 x 0.1 mm^3	
Theta range for data collection	3.678 to 66.591°.	
Index ranges	-28<=h<=26, -16<=k<=16, -50<=l<=50	
Reflections collected	71202	
Independent reflections	12490 [R(int) = 0.1193]	
Completeness to theta = 66.591°	99.8 %	
Absorption correction	Semi-empirical from equivalents	
Max. and min. transmission	1.00000 and 0.66443	
Refinement method	Full-matrix least-squares on F^2	
Data / restraints / parameters	12490 / 18 / 880	
Goodness-of-fit on F^2	1.105	
Final R indices [I>2sigma(I)]	R1 = 0.0800, wR2 = 0.2117	
R indices (all data)	R1 = 0.1054, wR2 = 0.2307	
Extinction coefficient	n/a	
Largest diff. peak and hole	1.333 and -1.551 e.Å^{-3}	
CCDC number	1959830	

Supplementary Table 7. Crystal data and structure refinement for **17**.

Identification code	exp_987_sq	
Empirical formula	C74 H62 N8 Pd2	
Formula weight	1276.11	
Temperature	105(2) K	
Wavelength	1.54184 Å	
Crystal system	Triclinic	
Space group	P-1	
Unit cell dimensions	a = 19.9184(5) Å	α= 72.736(4)°.
	b = 20.8947(8) Å	β= 66.193(3)°.
	c = 21.3554(10) Å	γ = 62.326(3)°.
Volume	7135.6(5) $Å^3$	
Z	4	
Density (calculated)	1.188 Mg/m^3	
Absorption coefficient	4.399 mm^{-1}	
F(000)	2616	
Crystal size	0.3 x 0.2 x 0.15 mm^3	
Theta range for data collection	3.537 to 66.600°.	
Index ranges	-23<=h<=18, -24<=k<=24, -25<=l<=25	
Reflections collected	50496	
Independent reflections	25199 [R(int) = 0.0563]	
Completeness to theta = 66.600°	99.9 %	
Absorption correction	Semi-empirical from equivalents	
Max. and min. transmission	1.00000 and 0.58440	
Refinement method	Full-matrix least-squares on F^2	
Data / restraints / parameters	25199 / 0 / 1536	
Goodness-of-fit on F^2	1.039	
Final R indices [I>2sigma(I)]	R1 = 0.0556, wR2 = 0.1477	
R indices (all data)	R1 = 0.0730, wR2 = 0.1623	
Extinction coefficient	n/a	
Largest diff. peak and hole	1.879 and -1.307 e.$Å^{-3}$	
CCDC number	1975890	

Supplementary Note 1. Upon treatment with TFA, **15** and **16** underwent mutual isomerization depending upon the reaction time and the concentration of TFA. To a $CDCl_3$ solution containing **15** (3 mg) or **16** (3 mg) into an NMR tube, 0.05, 0.2, 1.0, or 2.0 equivalents of TFA was added and the ^{1}H NMR spectra of the resultant solution were measured to determine the ratios of **15**/**16** at certain reaction time. The measurements confirmed that the isomerization became saturated after certain reaction time for both cases starting either from **15** and **16** as shown in Supplementary Figure 40-43.

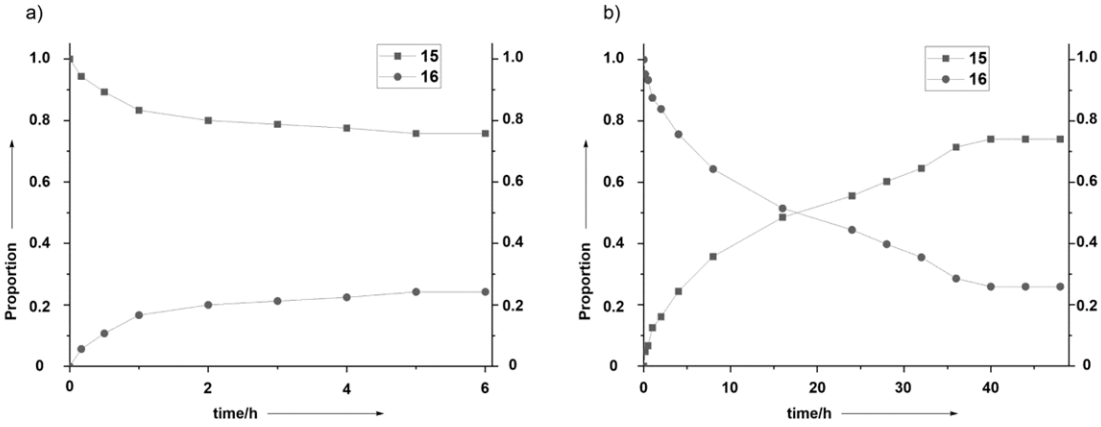

Supplementary Figure 40. (a) Proportion of **15/16** with 0.05 equivalent TFA in $CDCl_3$ (start from **15**), (b) proportion of **15/16** with 0.05 equivalent TFA in $CDCl_3$ (start from **16**)

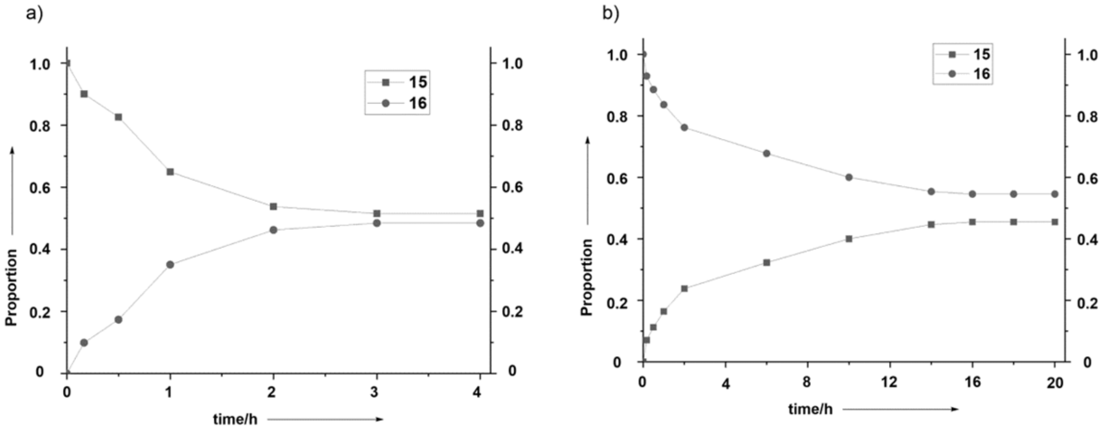

Supplementary Figure 41. (a) Proportion of **15/16** with 0.2 equiv TFA in $CDCl_3$ (start from **15**), (b) proportion of **15/16** with 0.2 equiv TFA in $CDCl_3$ (start from **16**)

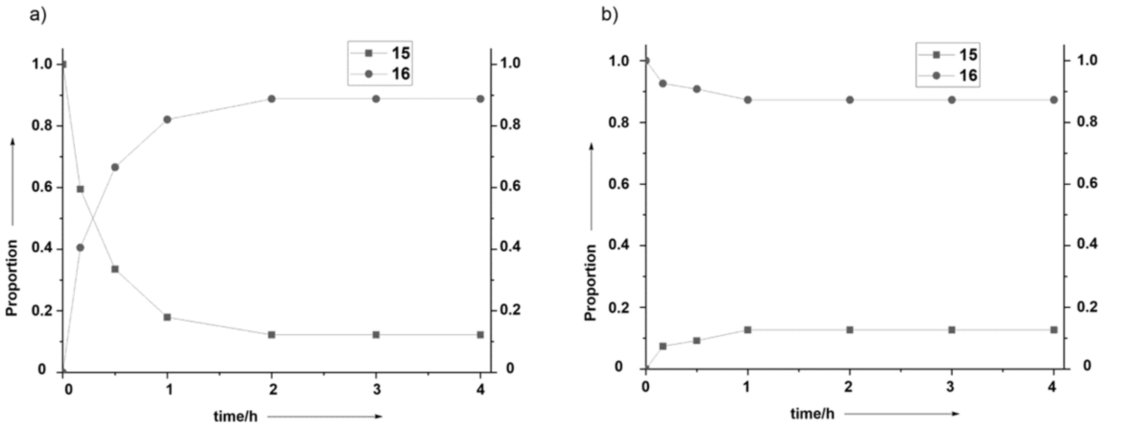

Supplementary Figure 42. (a) Proportion of **15/16** with 1.0 equivalent TFA in $CDCl_3$ (start from **15**), (b) proportion of **15/16** with 1.0 equivalent TFA in $CDCl_3$ (start from **16**)

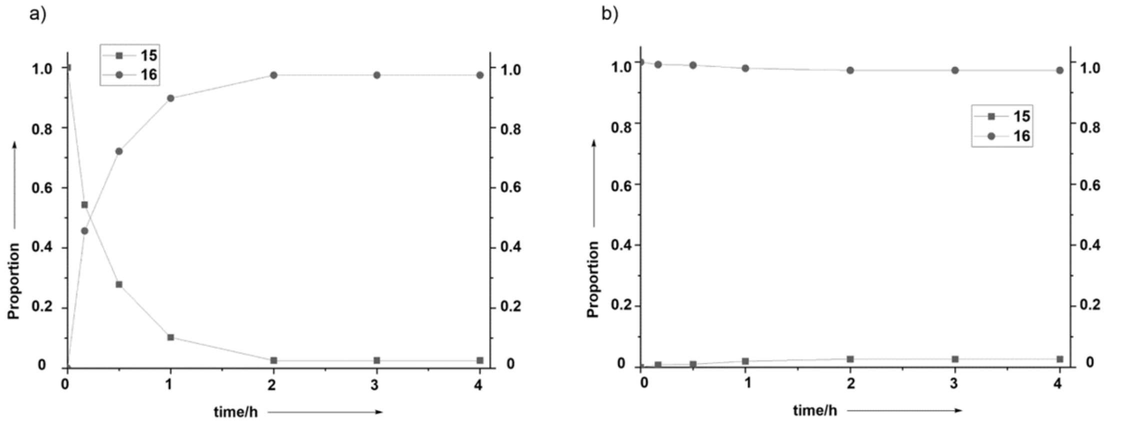

Supplementary Figure 43. (a) Proportion of **15/16** with 2.0 equivalent TFA in $CDCl_3$ (start from **15**), (b) proportion of **15/16** with 2.0 equivalent TFA in $CDCl_3$ (start from **16**)

Supplementary Note 2. These experiments revealed that the equilibrium ratio of **15**/**16** is related to the equivalent of the acid added. Hence 0.05-5 equivalent of TFA were added into a $CHCl_3$ solution of **15** or **16** respectively. After 48 h, the mixture was washed by water and extracted with $CHCl_3$. After removing all of the volatile the residual was dissolved in $CDCl_3$ and the ratio of **15**/**16** was determined by the ^{1}H NMR spectra.

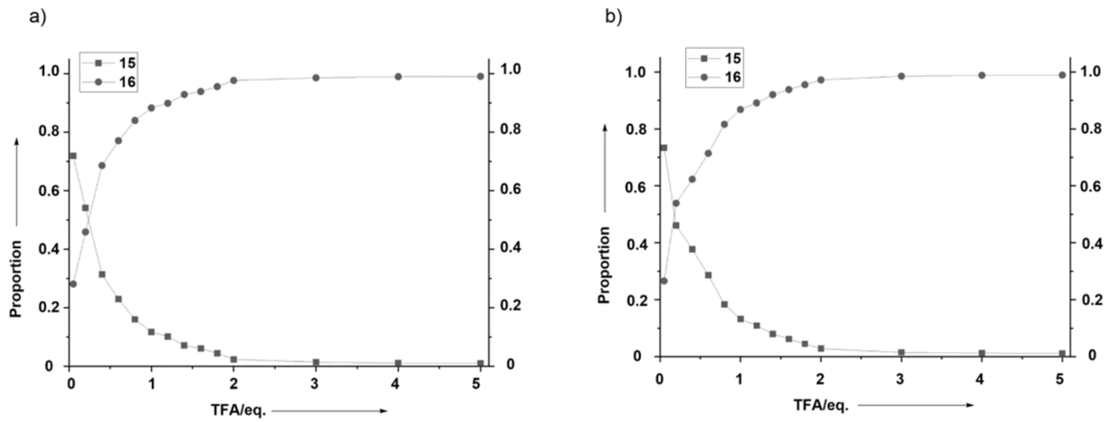

Supplementary Figure 44. (a) Equilibrium proportion of **15** and **16** in various equivalents of TFA in $CDCl_3$ (start from **15**), (b) equilibrium proportion of **15** and **16** in various equivalents of TFA in $CDCl_3$ (start from **16**)

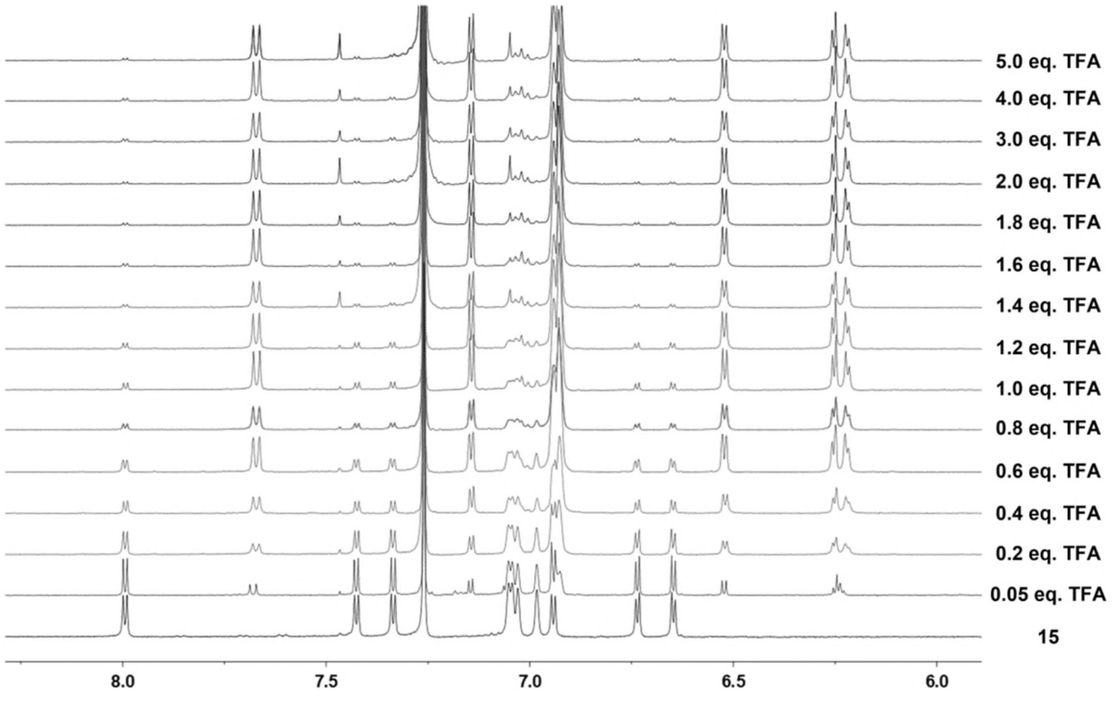

Supplementary Figure 45. ^{1}H NMR spectra of equilibrium mixture of **15/16** in various equivalents of TFA in $CDCl_3$. (start from **15**)

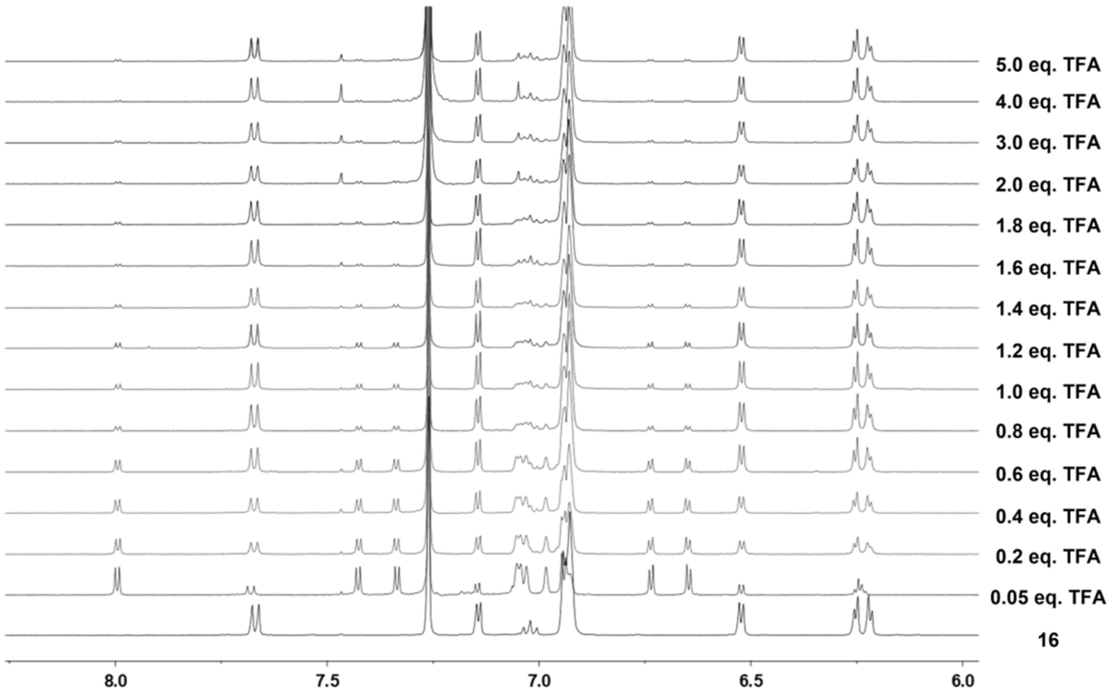

Supplementary Figure 46. ^{1}H NMR spectra of equilibrium mixture of **15/16** in various equivalents of TFA in $CDCl_3$. (start from **16**).

Supplementary Note 3. TFA-d was applied to promote the conversion from **15** to **16**. This procedure was monitored by ^{1}H NMR. In the figure below (a) shows the ^{1}H NMR spectrum of **15** (as S.M.). After the TFA-d was added and washed by water the spectrum (b) was recorded. (c) is the ^{1}H NMR spectrum of pure **16**, which is synthesized using undeuterated TFA. (b) illustrates signals with similar chemical shifts and multiplets to those in (c) except those peaks ascribed to proton c. These spectra indicated that C-H activation and the following C-Pd bonds deuteration underwent in the isomerization process.

Supplementary Figure 56 and Supplementary Figure 57 reveal relative energy of these intermediates with the help DFT calculation. Experimentally we added $CDCl_3$ to dissolve **15** in NMR tube and 0.25 mL TFA-d was then added, the mixture was

monitored by ^{1}H NMR afterward. Unfortunately, we can not identify the intermediate via in-situ ^{1}H NMR. After washing with water, we observed that **16** was generated as the sole product.

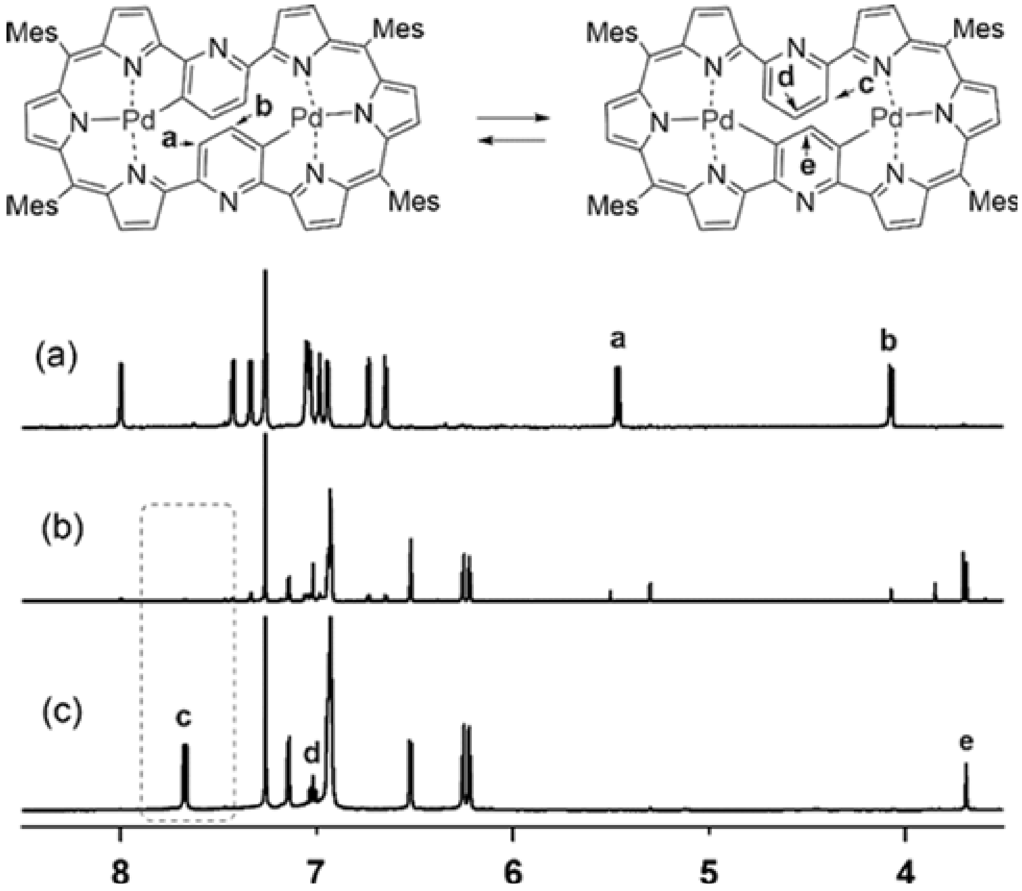

Supplementary Figure 47. TFA-d was applied to promote the conversion from **15** to **16**. (a) shows the ^{1}H NMR spectrum of **15** (as S.M.). After the TFA-d was added and washed by water the spectrum (b) was recorded. (c) is the ^{1}H NMR spectrum of pure **16**.

Supplementary Notes 4. All calculations were carried out using the *Gaussian 09* program.[1] Initial geometries for **12-17** were obtained from X-ray structures. The structures were fully optimized without any symmetry restriction. Geometry optimizations in the ground state (S_0) were performed by the density functional theory (DFT) method with restricted B3LYP (Becke's three-parameter hybrid exchange

functionals and the Lee-Yang-Parr correlation functional)[2] level employing basis sets and pseudopotentials; 6-311G(d,p) for C, H, N[3] and SDD for Pd.[4] NICS(0) values were calculated with GIAO method at the B3LYP level employing the same basis sets and pseudopotentials for geometry optimizations. Calculated chemical shifts were estimated relative to the magnetic shielding of a proton of chloroform (24.95 ppm) calculated at the same level.

Supplementary Table 8. Calculated NICS(0) values (pink point) and magnetic shielding effects of **12**. A top right figure shows correlation between the calculated (GIAO/B3LYP/6-311G(d, p) ^{1}H NMR shifts and the corresponding experimental values (R^2 = 0.991).

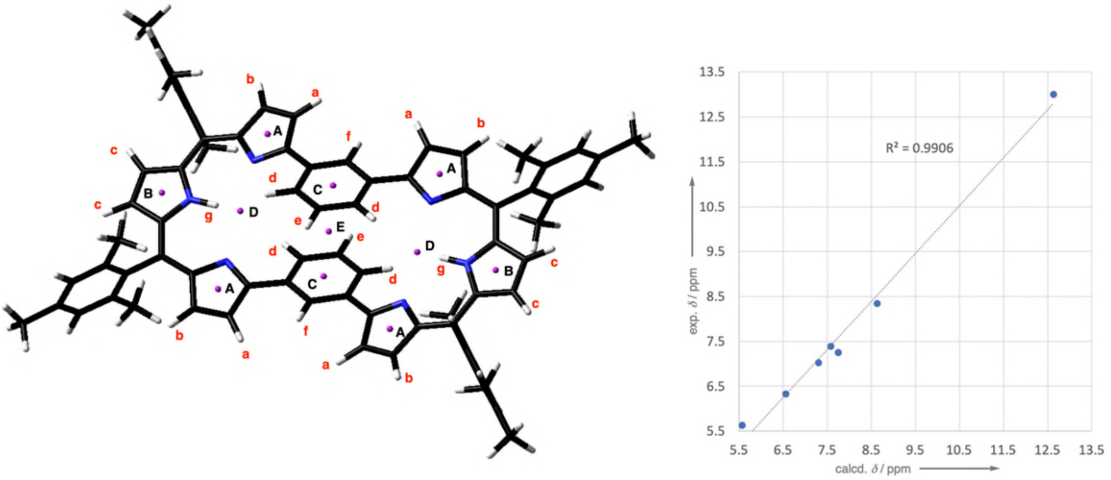

	A	B	C	D	E		
NICS(0)	−0.80	−8.21	−8.52	+0.66	−11.87		
	a	b	c	d	e	f	g
δ_{calc}	7.58	7.30	6.56	7.75	5.57	8.63	12.63
δ_{exp}	7.39	7.03	6.33	7.25	5.63	8.34	13.00

*The point E was defined as the weighted center of 40 carbon and 6 nitrogen atoms (blue) consisting of the octaphyrin ligand. The large negative NICS(0) value at the point E (–11.87 ppm) is ascribed to the magnetic shielding effect of ring C, and not to global aromaticity of the octaphyrin because the NICS(0) value of point D (the midpoint of two nitrogen atoms of ring A) is only 0.66 ppm.

Supplementary Table 9. Calculated NICS(0) values (pink point) and magnetic shielding effects of **13**. A top right figure shows correlation between the calculated (GIAO/B3LYP/6-311G(d, p)) ^{1}H NMR shifts and the corresponding experimental values (R^2 = 0.998).

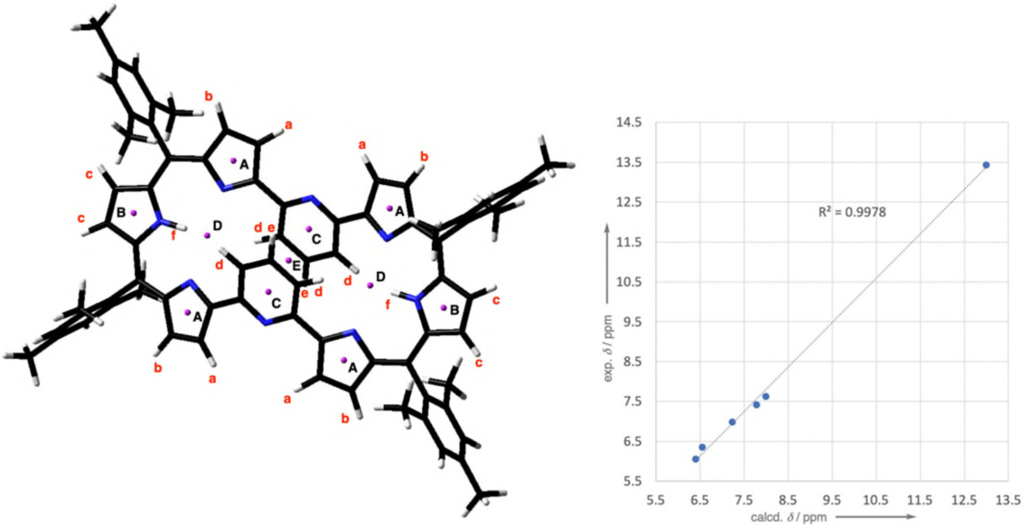

	A	B	C	D	E	
NICS(0)	−0.73	−8.05	−7.07	+0.74	−12.18	
	a	b	c	d	e	f
δ_{calc}	7.99	7.23	6.55	7.78	6.40	12.99
δ_{exp}	7.63	6.98	6.35	7.41	6.05	13.43

*The point E was defined as the weighted center of 38 carbon and 8 nitrogen atoms (blue) consisting of the octaphyrin ligand. The large negative NICS(0) value at the point E (–12.18 ppm) is ascribed to the magnetic shielding effect of ring C, and not to global aromaticity of the octaphyrin because the NICS(0) value of point D (the midpoint of two nitrogen atoms of ring A) is only 0.74 ppm.

Supplementary Table 10. Calculated NICS(0) values (pink point) and magnetic shielding effects of **14**. A top right figure shows correlation between the calculated (GIAO/B3LYP/6-311G(d, p)+SDD (for Pd (indigo))) ^{1}H NMR shifts and the corresponding experimental values (R^2 = 0.993).

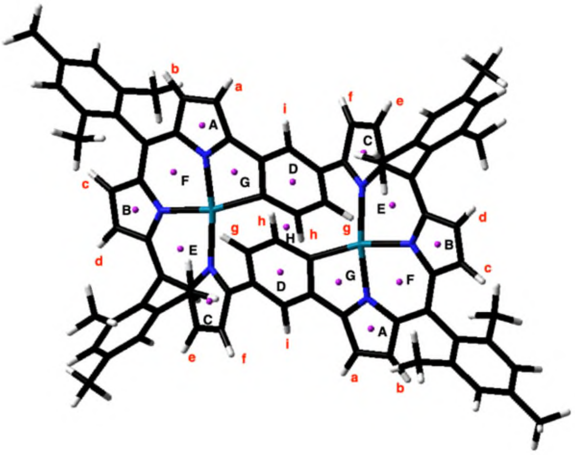

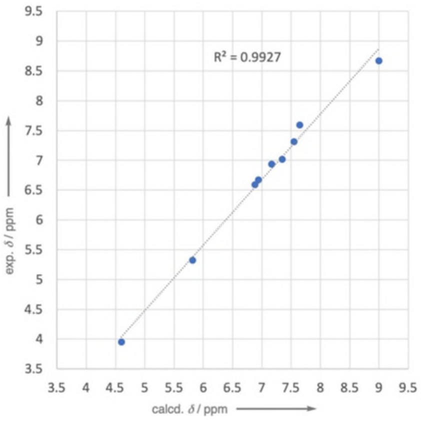

	A	B	C	D	E	F	G	H	
NICS(0)	−1.13	−3.91	−0.67	−8.35	−1.28	+1.12	+1.62	−14.94	
	a	b	c	d	e	f	g	h	i
δ_{calc}	7.35	7.17	6.94	6.88	7.55	7.65	5.82	4.60	9.00
δ_{exp}	7.02	6.94	6.67	6.59	7.31	7.59	5.32	3.95	8.67

*The point H was defined as the weighted center of 40 carbon and 6 nitrogen atoms (blue) consisting of the octaphyrin ligand. The large negative NICS(0) value at the point H (–14.94 ppm) is ascribed to the magnetic shielding effect of ring D, and not to global aromaticity of the octaphyrin.

Supplementary Table 11. Calculated NICS(0) values and magnetic shielding effects of **15**. A top right figure shows correlation between the calculated (GIAO/B3LYP/6-311G(d, p)+SDD (for Pd (indigo))) ^{1}H NMR shifts and the corresponding experimental values (R^2 = 0.996).

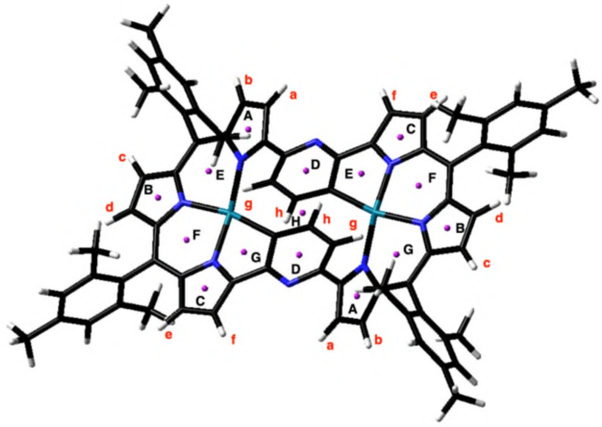

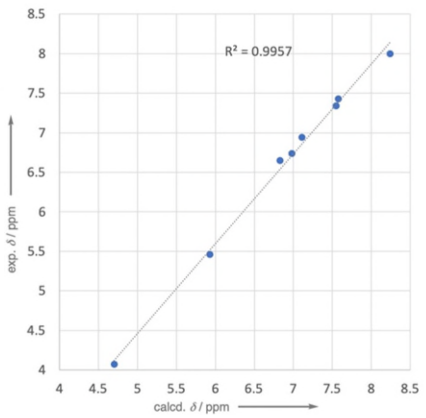

	A	B	C	D	E	F	G	H
NICS(0)	−0.30	−3.88	−1.08	−7.73	−1.08	+1.64	+2.05	−15.18
	a	b	c	d	e	f	g	h
δ_{calc}	8.24	7.55	6.83	6.98	7.11	7.58	5.93	4.70
δ_{exp}	8.00	7.34	6.65	6.74	6.94	7.43	5.46	4.07

*The point H was defined as the weighted center of 38 carbon and 8 nitrogen atoms (blue) consisting of the octaphyrin ligand. The large negative NICS(0) value at the point H (–15.18 ppm) is ascribed to the magnetic shielding effect of ring D, and not to global aromaticity of the octaphyrin.

Supplementary Table 12. Calculated NICS(0) values (pink point) and magnetic shielding effects of **16**. A top right figure shows correlation between the calculated (GIAO/B3LYP/6-311G(d, p)+SDD (for Pd (indigo))) ^{1}H NMR shifts and the corresponding experimental values (R^2 = 0.990).

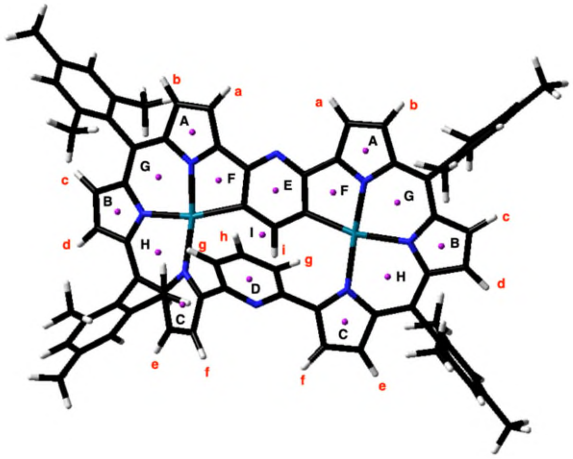

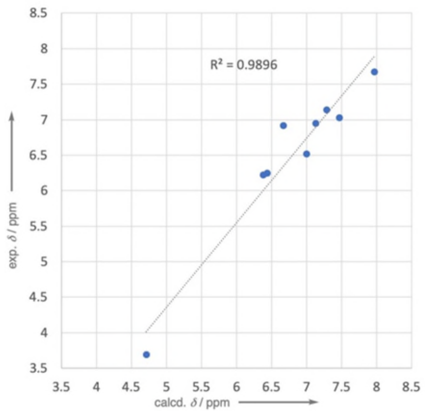

	A	B	C	D	E	F	G	H	I
NICS(0)	−0.01	−4.07	−1.13	−8.31	−2.11	+4.05	+4.21	+1.61	−13.70
	a	b	c	d	e	f	g	h	i
δ_{calc}	7.00	6.67	6.38	6.44	7.13	7.29	7.97	7.47	4.71
δ_{exp}	6.52	6.92	6.22	6.25	6.95	7.14	7.67	7.03	3.69

*The point I was defined as the weighted center of 38 carbon and 8 nitrogen atoms (blue) consisting of the octaphyrin ligand. The large negative NICS(0) value at the point I (–13.70 ppm) is ascribed to the magnetic shielding effect of ring D and E, and not to global aromaticity of the octaphyrin.

Supplementary Table 13. Calculated NICS(0) values (pink point) and magnetic shielding effects of **17**. A top right figure shows correlation between the calculated (GIAO/B3LYP/6-311G(d, p)+SDD (for Pd(indigo))) ^{1}H NMR shifts and the corresponding experimental values (R^2 = 0.953).

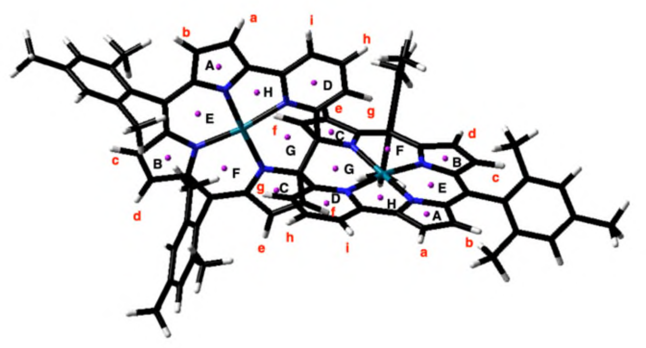

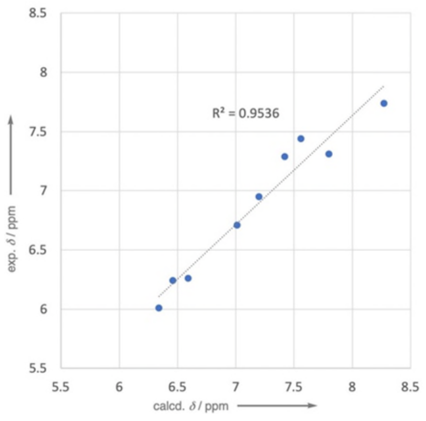

	A	B	C	D	E	F	G	H	
NICS(0)	−9.07	−0.89	+0.57	−2.89	−2.90	–3.50	+3.70	+1.57	
	a	b	c	d	e	f	g	h	i
δ_{calc}	7.20	6.59	7.01	6.46	6.34	8.27	7.80	7.56	7.42
δ_{exp}	6.95	6.26	6.71	6.24	6.01	7.74	7.31	7.44	7.29

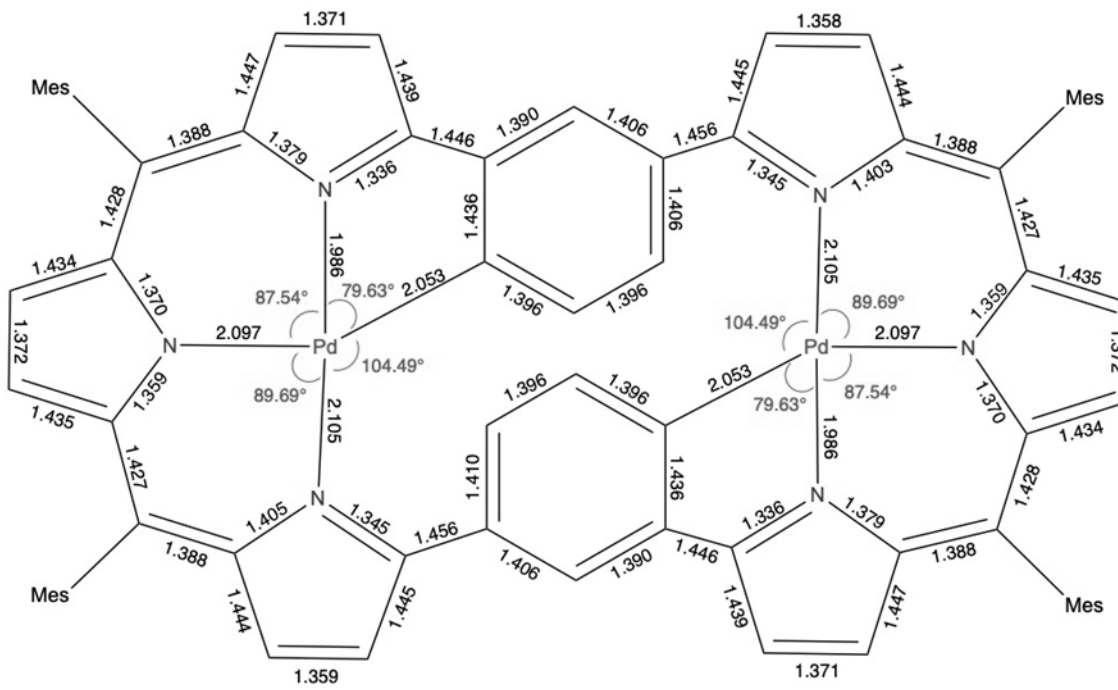

Supplementary Figure 48. Calculated bond lengths (in Å) and selected angles of **14**.

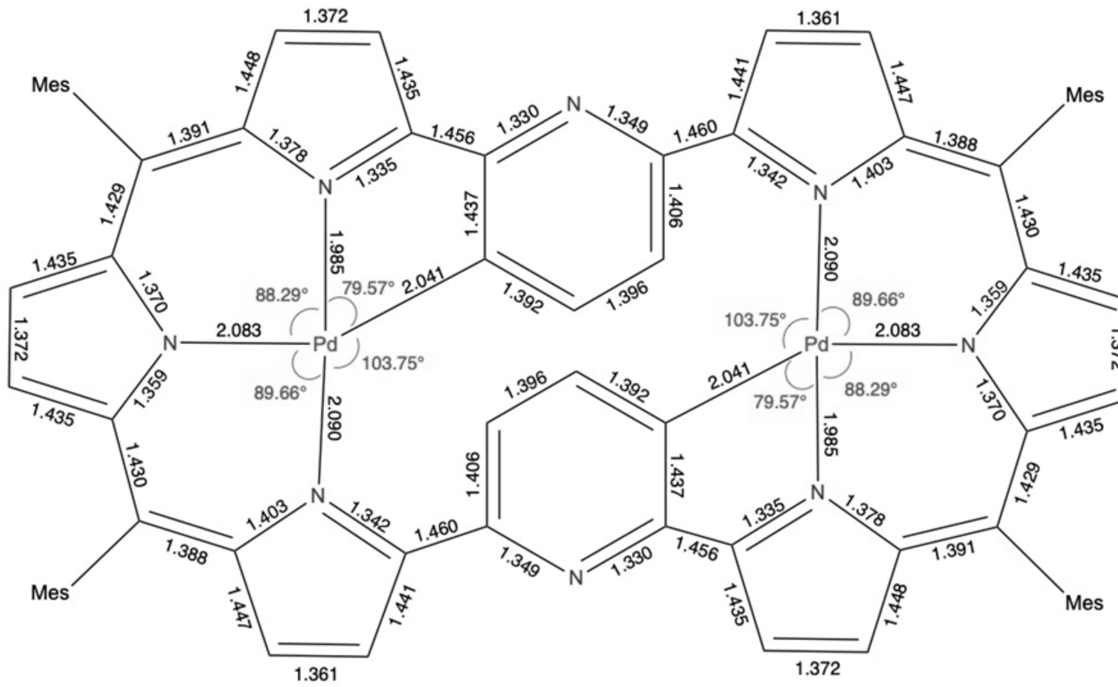

Supplementary Figure 49. Calculated bond lengths (in Å) and selected angles of **15**.

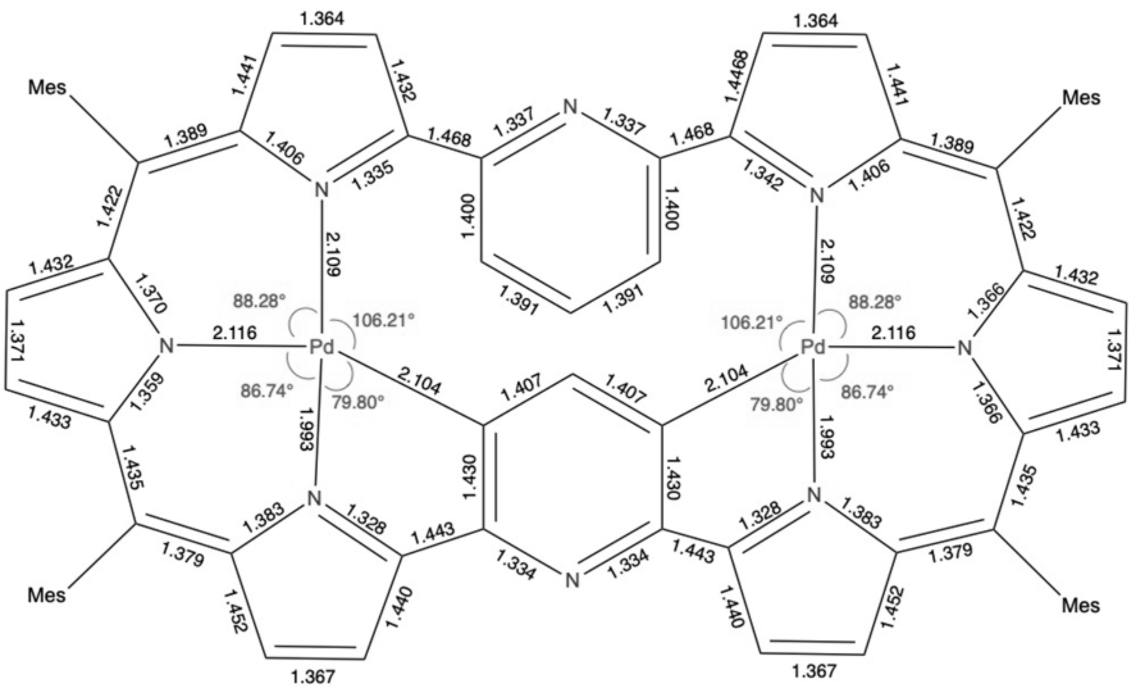

Supplementary Figure 50. Calculated bond lengths (in Å) and selected angles of **16**.

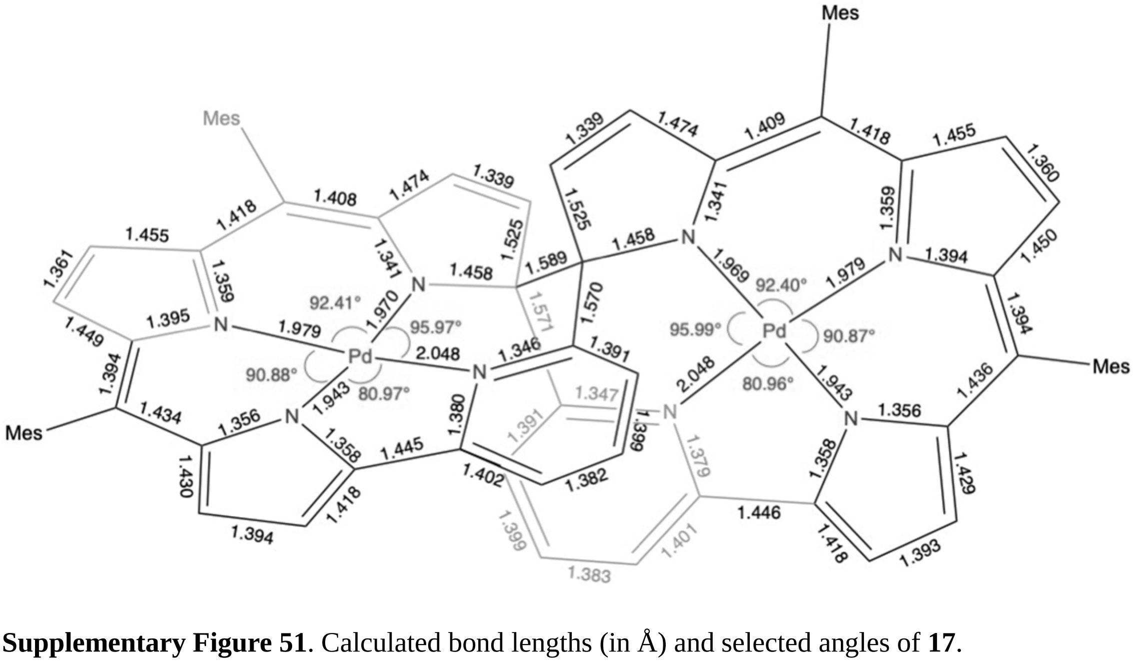

Supplementary Figure 51. Calculated bond lengths (in Å) and selected angles of **17**.

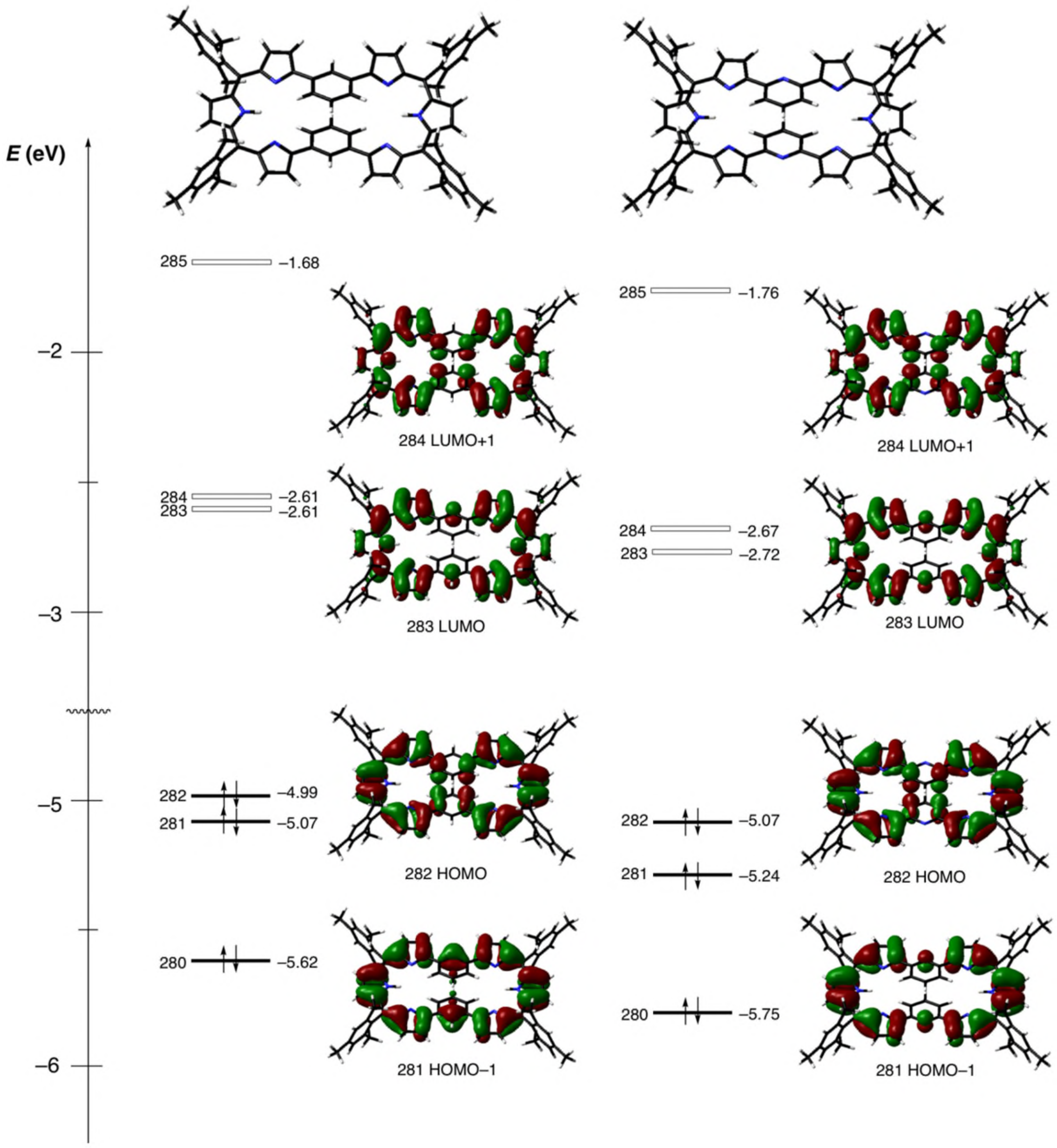

Supplementary Figure 52. Molecular orbital energy diagrams and Kohn-Sham MO representations of **12** and **13**. Red and green indicate different signs of wave functions of molecular orbitals.

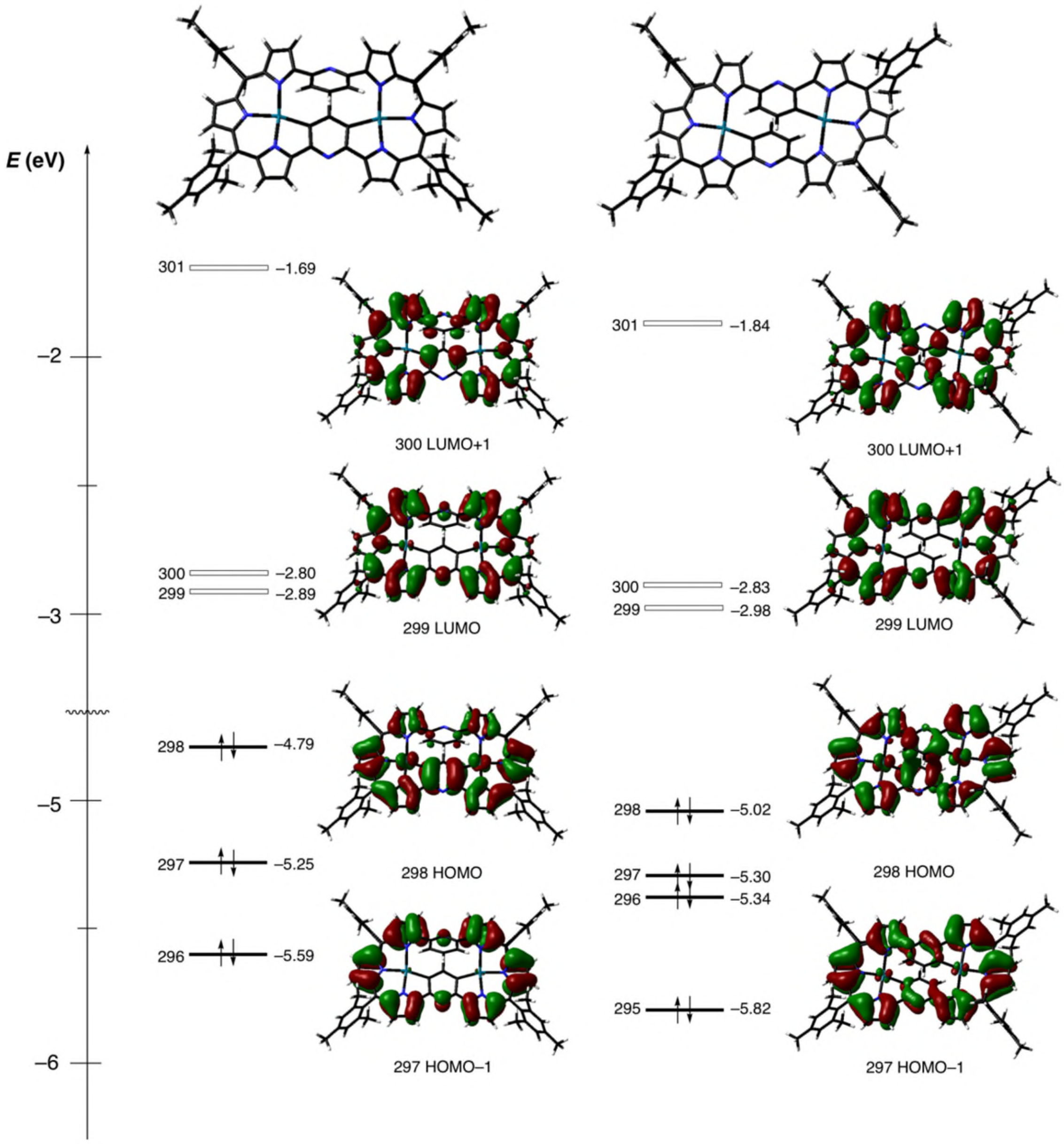

Supplementary Figure 53. Molecular orbital energy diagrams and Kohn-Sham MO representations of **15** and **16**. Red and green indicate different signs of wave functions of molecular orbitals.

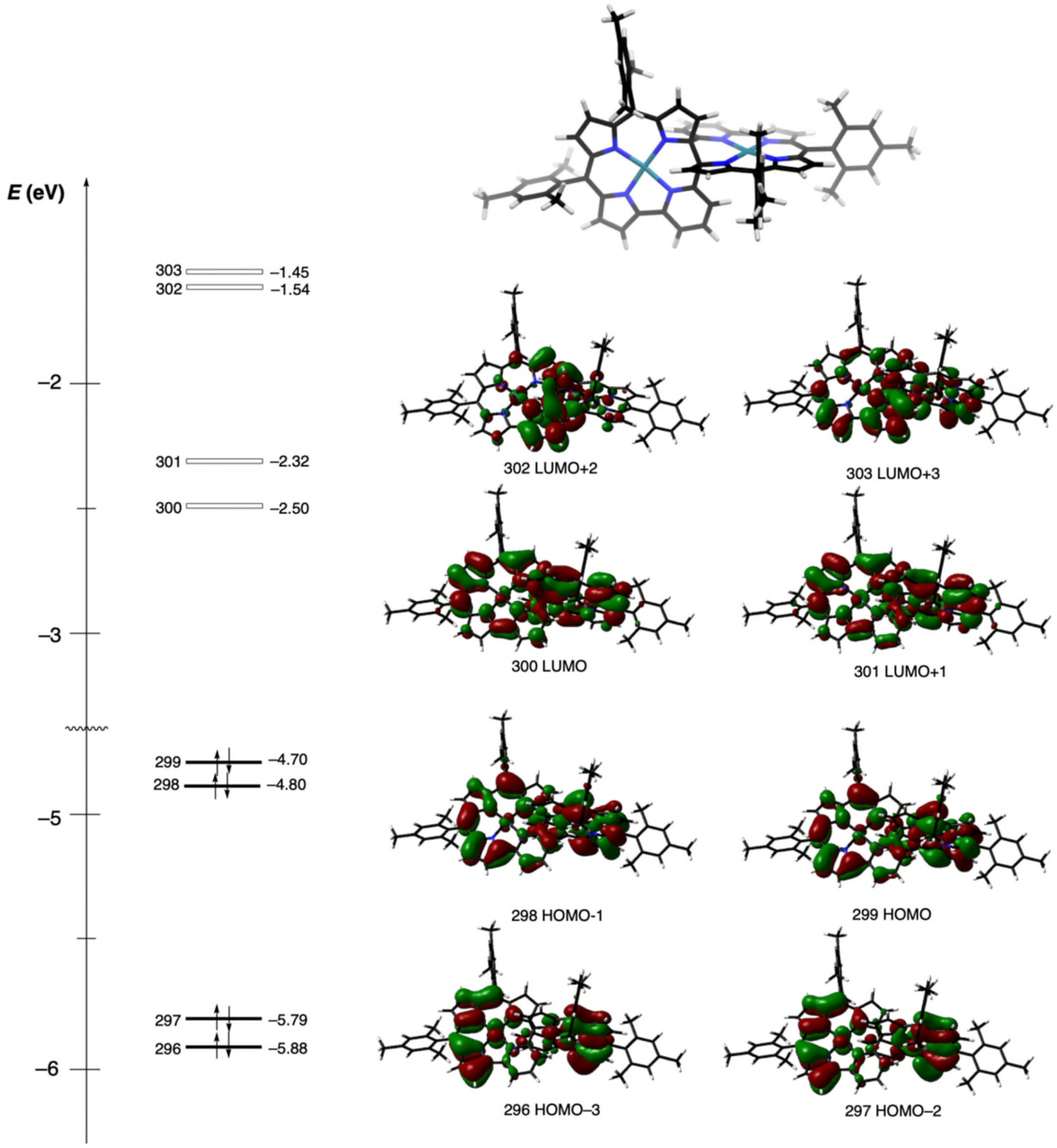

Supplementary Figure 54. Molecular orbital energy diagrams and Kohn-Sham MO representations of **17**. Red and green indicate different signs of wave functions of molecular orbitals.

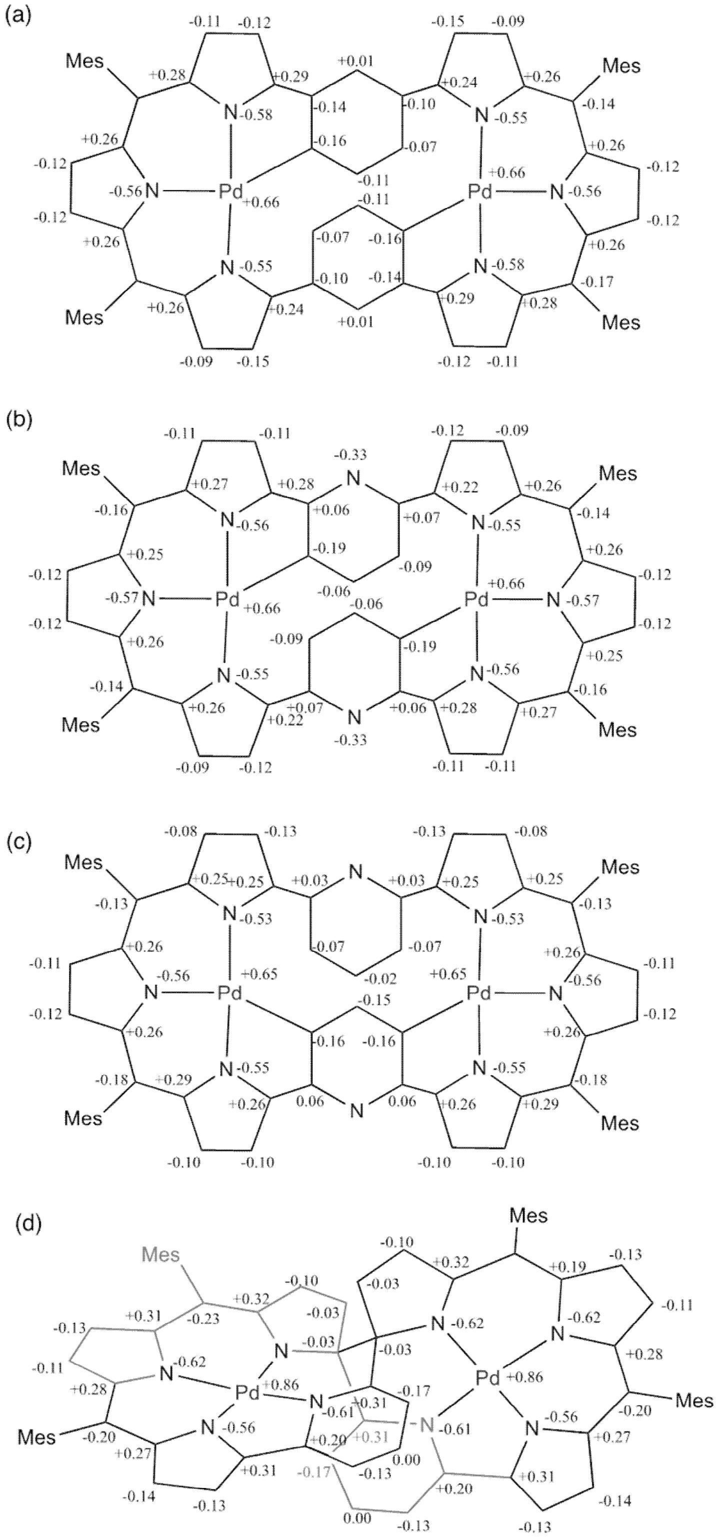

Supplementary Figure 55. Calculated Mulliken atomic charge values of (a) **14**, (a) **15**, (a) **16**, and (a) **17**.

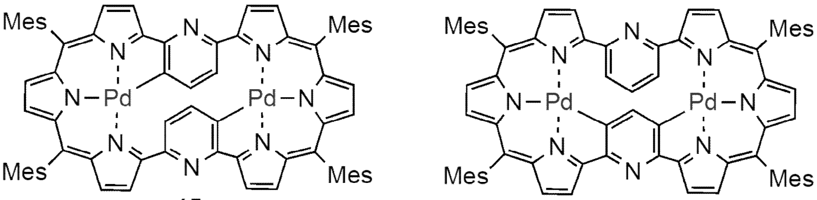

15 **16**

cmpd.	E + ZPE (hartree)	ΔH (kcal/mol)	ΔG (kcal/mol)
**15**	−3549.6293	+0.088	+2.69
**16**	−3549.6302	0	0

Supplementary Figure 56. Summary of electronic energies including zero-point-energy correction (*E* +*ZPE*), relative electronic and thermal enthalpies (*ΔH*), and relative Gibbs free energies (*ΔG*) for **15** and **16**. Calculation was performed by DFT at the level of B3LYP/6-311G(d, p) (for C,H,N) + SDD (for Pd) level.

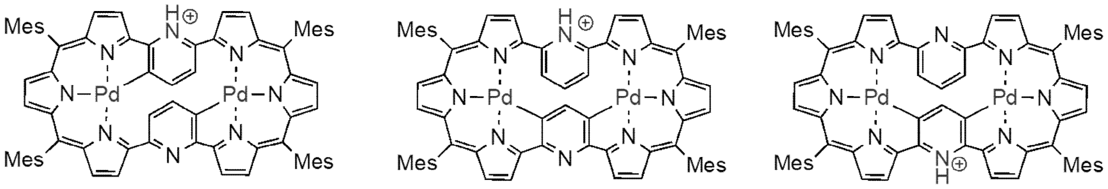

15H **16Ha** **16Hb**

cmpd.	E + ZPE (hartree)	ΔH (kcal/mol)	ΔG (kcal/mol)
**15H**	−3550.0307	+4.74	+8.95
**16Ha**	−3550.0259	+8.21	+9.52
**16Hb**	−3550.0391	0	0

Supplementary Figure 57. Summary of electronic energies including zero-point-energy correction (*E* +*ZPE*), relative electronic and thermal enthalpies (*ΔH*), and relative Gibbs free energies (*ΔG*) for **15H**, **16Ha** and **16Hb**. Calculation was performed by DFT at the level of B3LYP/6-311G(d, p) (for C,H,N) + SDD (for Pd) level.

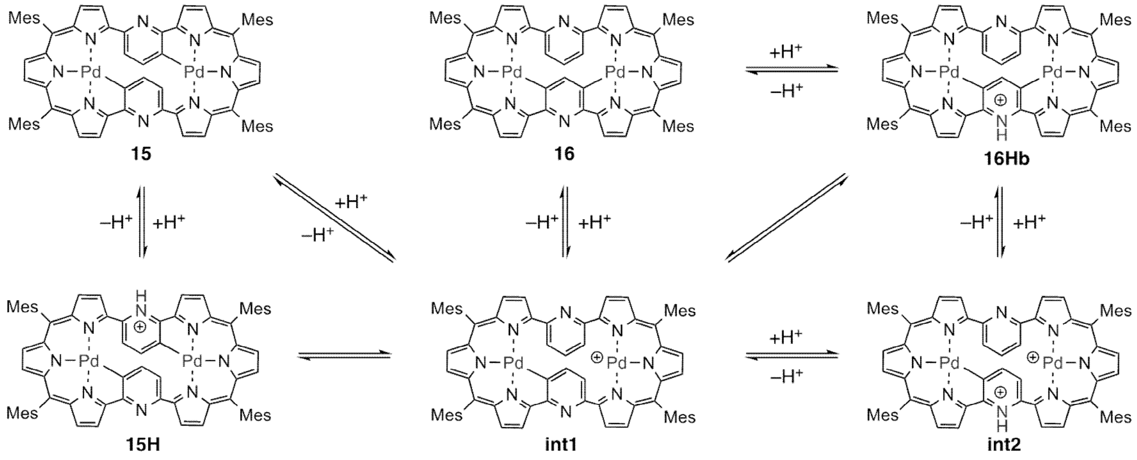

Supplementary Figure 58. Possible interconversion pathway between **15** and **16**.

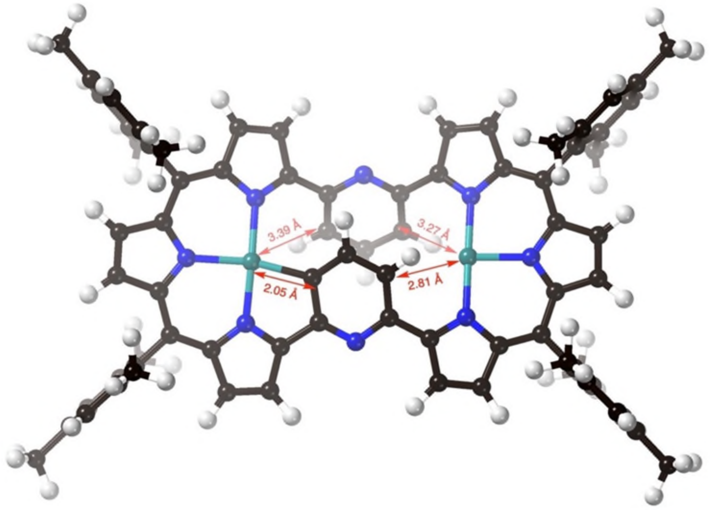

Supplementary Figure 59. Calculated structure and selected bond lengths of **int1**. Carbon atom, black; nitrogen atom, blue; palladium atom, indigo; hydrogen atom, white.

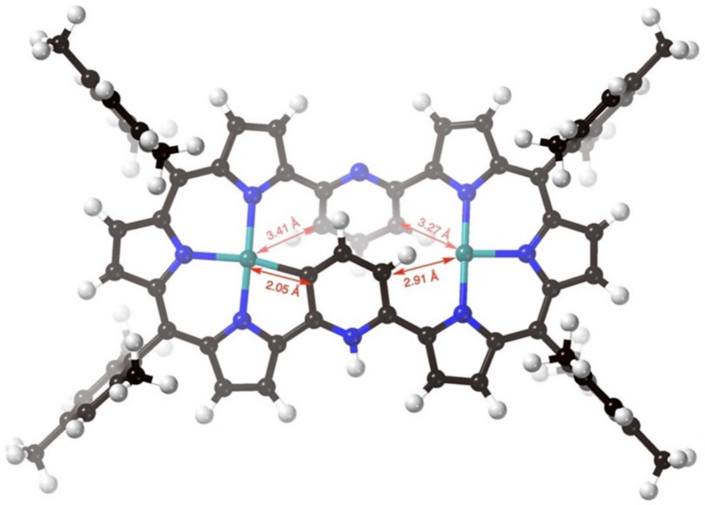

Supplementary Figure 60. Calculated structure and selected bond lengths of **int2**. Carbon atom, black; nitrogen atom, blue; palladium atom, indigo; hydrogen atom, white.

Supplementary References

[1] Gaussian 09, Revision C.01, M. J. Frisch, G. W. Trucks, H. B. Schlegel, G. E. Scuseria, M. A. Robb, J. R. Cheeseman, G. Scalmani, V. Barone, B. Mennucci, G. A. Petersson, H. Nakatsuji, M. Caricato, X. Li, H. P. Hratchian, A. F. Izmaylov, J. Bloino, G. Zheng, J. L. Sonnenberg, M. Hada, M. Ehara, K. Toyota, R. Fukuda, J. Hasegawa, M. Ishida, T. Nakajima, Y. Honda, O. Kitao, H. Nakai, T. Vreven, J. A. Montgomery, Jr., J. E. Peralta, F. Ogliaro, M. Bearpark, J. J. Heyd, E. Brothers, K. N. Kudin, V. N. Staroverov, R. Kobayashi, J. Normand, K. Raghavachari, A. Rendell, J. C. Burant, S. S. Iyengar, J. Tomasi, M. Cossi, N. Rega, J. M. Millam, M. Klene, J. E. Knox, J. B. Cross, V. Bakken, C. Adamo, J. Jaramillo, R. Gomperts, R. E. Stratmann, O. Yazyev, A. J. Austin, R. Cammi, C. Pomelli, J. W. Ochterski, R. L. Martin, K. Morokuma, V. G. Zakrzewski, G. A. Voth, P. Salvador, J. J. Dannenberg, S. Dapprich, A. D. Daniels, Ö. Farkas, J. B. Foresman, J. V. Ortiz, J. Cioslowski, D. J. Fox, Gaussian, Inc., Wallingford CT, **2009**.

[2] Becke, A. D. A new mixing of Hartree–Fock and local density - functional theories. *J. Chem. Phys.* **98**, 1372-1377 (1993). (b) Lee, C., Yang, W. & Parr, R. G. Development of the Colle-Salvetti correlation-energy formula into a functional of the electron density. *Phys. Rev. B* **37**, 785-789 (1998)

[3] Krishnan, R., Binkley, J. S., Seeger, R., Pople, J. A. Self-consistent molecular orbital methods. XX. A basis set for correlated wave functions. *J. Chem. Phys.* **72**, 650-654 (1980)

[4] Dolg, M., Wedig, U., Stoll, H. & Preuss, H. Energy - adjusted ab initio pseudopotentials for the first row transition elements. *J. Chem. Phys.* **86**, 866-872 (1987)
